# NF-κB inducing kinase (NIK) deletion accelerates KRAS-driven pancreatic cancer in association with tumor microenvironment remodeling

**DOI:** 10.1038/s41419-026-08877-w

**Published:** 2026-05-27

**Authors:** Ziwei Du, Ulrike F. G. Büttner, Hannah Lea Wirth, Melanie Gerstenlauer, Uta Manfras, Rikarda Loidl, Doğa Bahçeci, Katja Steiger, Thomas Metzler, Lap Kwan Chan, Miltiadis Tsesmelis, Thomas Wirth

**Affiliations:** 1 Institute of Physiological Chemistry, University of Ulm, Ulm, Baden-Württemberg Germany; 2 Institute of Pathology, Technical University Munich, Munich, Bayern Germany; 3 Comparative Experimental Pathology (CEP), School of Medicine and Health, Technical University Munich, Munich, Bayern Germany; 4 Department of Pathology and Molecular Pathology, University Hospital of Zurich, Zurich, Switzerland; 5 Single-Cell Sequencing Unit, University of Ulm, Ulm, Baden-Württemberg Germany

**Keywords:** Pancreatic cancer, Cancer microenvironment, Stress signalling, Mechanisms of disease, Kinases

## Abstract

Pancreatic ductal adenocarcinoma (PDAC) is a highly lethal cancer marked by dense stroma, immune suppression, and therapy resistance. While canonical NF-κB signaling has been extensively studied in PDAC, the non-canonical pathway and its key kinase, NF-κB–inducing kinase (NIK), remain less characterized. Here, we employed genetically engineered mouse models, including Pdx1-Cre; KRAS^G12D^ (KC), Pdx1-Cre; KRAS^G12D^; p53^fl/fl^ (KPC), and their NIK-deficient counterparts (KNiC and KPNiC). To mimic inflammation-driven tumorigenesis observed in human PDAC, we administered cerulein in the KC and KNiC mice to induce pancreatitis and promote lesion progression. We found that NIK deletion accelerated lesion formation, progression to high-grade PanINs and invasive carcinoma in both KC and KPC backgrounds. Despite similar tumor grade and burden at endpoint, KPNiC mice exhibited shortened survival compared to controls, indicating that NIK acts as a tumor-suppressor and limits early-stage tumor progression. Mechanistically, NIK loss was associated with elevated ERK signaling that increased lesion cell proliferation, and also reduced acinar cell death following pancreatitis. In the tumor microenvironment, NIK deletion promoted myofibroblast activation and enrichment of myCAF-associated gene expression, likely through secondary activation of canonical NF-κB and pro-fibrotic signaling pathways such as TGF-β, Wnt, and FGF. Finally, increased IL6–STAT3 signaling and neutrophil infiltration were observed, suggesting broader immunostromal remodeling in the absence of NIK. Consistent with these findings, analysis of TCGA-PAAD data showed that low NIK expression correlates with poor overall survival in human PDAC. These findings reveal a tumor-suppressive role of NIK in pancreatic cancer and underscore the need for caution in targeting NF-κB signaling, as balanced pathway activity appears critical for regulating early tumor progression and microenvironmental interactions in PDAC.

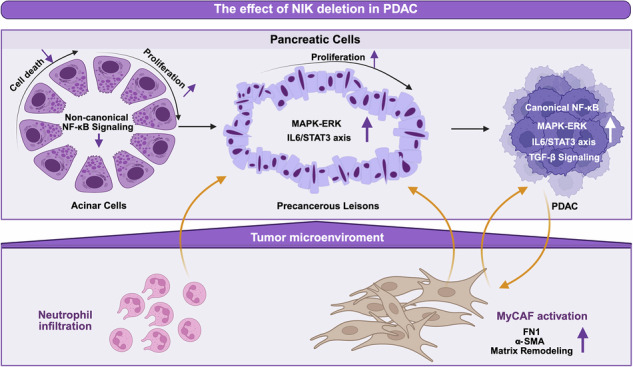

## Introduction

Pancreatic cancer remains one of the most lethal malignancies, with a five-year survival rate below 10%, largely due to its dense desmoplastic stroma, immune evasion mechanisms, and high metastatic potential, all of which contribute to pronounced chemoresistance [1]. Pancreatic ductal adenocarcinoma (PDAC), the predominant histological subtype, is thought to arise from acinar-ductal metaplasia (ADM), progressing sequentially through low-grade and high-grade pancreatic intraepithelial neoplasia (PanIN) before developing into invasive carcinoma [2, 3].

Oncogenic KRAS mutations are a hallmark of PDAC, present in over 95% of human cases. In genetically engineered mouse models, mutant KRAS alone is sufficient to initiate PanIN lesions formation, but additional events, such as TP53 inactivation or chronic pancreatitis, can accelerate their progression to invasive PDAC [4, 5]. Experimentally, cerulein-induced pancreatitis mimics human disease by creating a pro-inflammatory microenvironment that promotes tumorigenesis [6].

While the canonical NF-κB pathway has been extensively studied in PDAC pathogenesis, the non-canonical NF-κB pathway remains poorly understood. The NF-κB family consists of dimeric transcription factors formed by NFKB1/p105, NFKB2/p100, RelA/p65, RelB, and c-Rel [7]. In the non-canonical pathway, the p100:RelB complex remains sequestered in the cytoplasm due to continuous degradation of NF-κB-inducing kinase (NIK) by the TRAF2-TRAF3-cIAP complex. Upon receptor activation, TRAF3 is degraded, leading to NIK stabilization. NIK then phosphorylates and activates IKKα, which in turn mediates p100 processing into p52. The p52:RelB heterodimer translocates to the nucleus, where it regulates gene transcription [8].

In this study, we investigated the role of NIK in pancreatic tumorigenesis using the well-established Pdx1-Cre; KRAS^G12D^ (KC) and Pdx1-Cre; KRAS^G12D^; p53^fl/fl^ (KPC) mouse models [9, 10]. Our findings indicate that NIK deletion accelerates the formation of low-grade PanIN lesions, their progression to higher-grade neoplasias, and the development of PDAC while supporting a pro-myCAF microenvironment.

## Results

### NIK deletion accelerates progression to pancreatic carcinoma

**Fig. 1:**
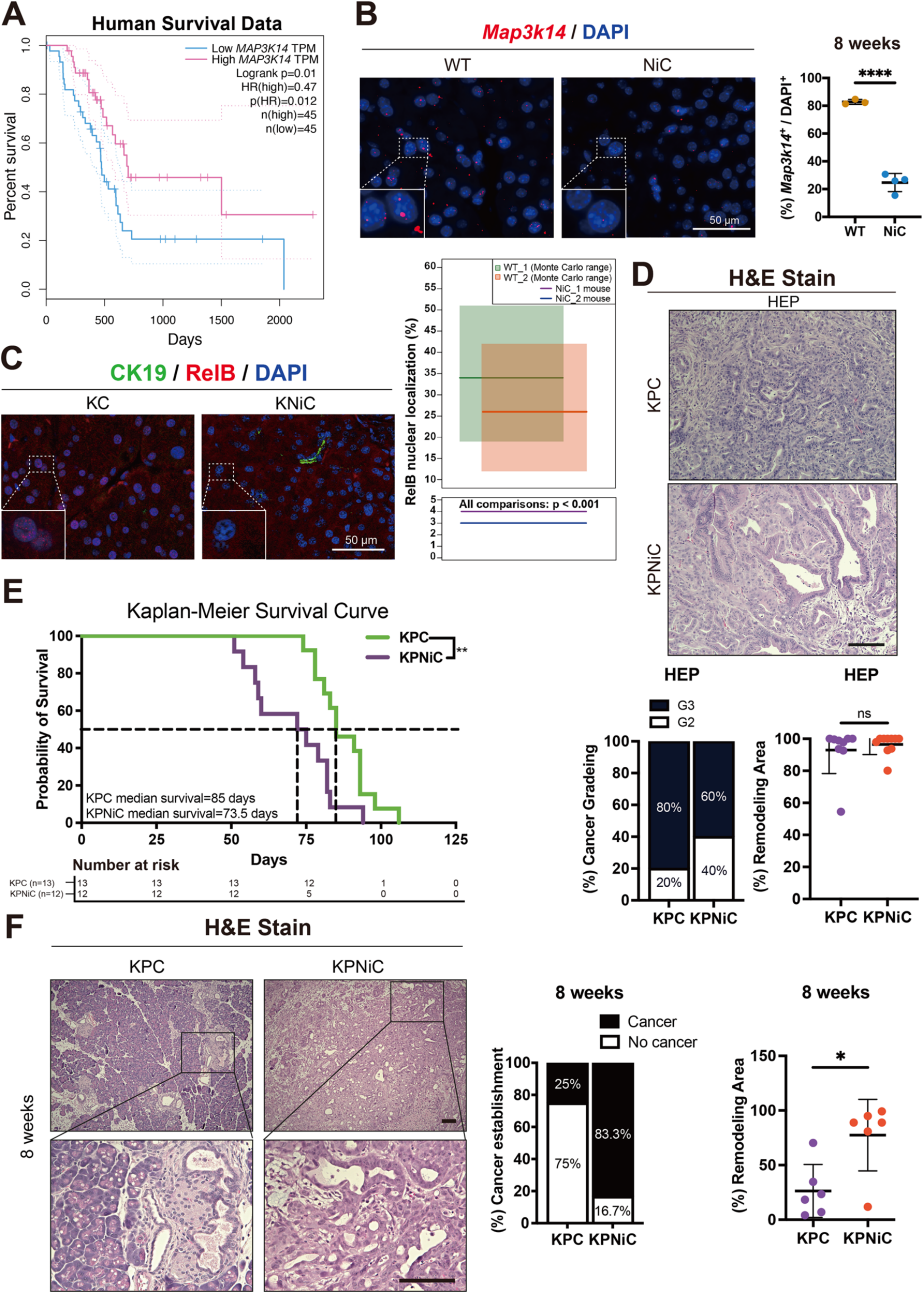
NIK deletion accelerates progression to pancreatic carcinoma. **A** Kaplan–Meier survival analysis of TCGA-PAAD patients with high (pink line) vs. low (blue line) expression of *MAP3K14* (NIK). The graph was generated with GEPIA. *p* = 0.01, log-rank test. **B** Left: RNAscope in situ hybridization of *Map3k14* in WT and NiC mice. Red: *Map3k14*, blue: DAPI. Scale bar: 50 μm. Right: Quantification of *Map3k14*^+^/DAPI⁺ signals (%). *N* ≥ 3 mice/group. *t* = 8 weeks. Unpaired Student’s *t* test. **C** Left: Pancreatic sections were stained for CK19 (green; ductal marker), and RelB (red; non-canonical NF-κB subunit). Scale bar: 100 μm. Right: Quantification of RelB⁺ nuclei with Monte Carlo resampling (1,000 iterations per WT mouse); *N* ≥ 2 mice/group. *t* = 8 weeks. Each KNiC mouse comparison was significant in all four WT–KO pairwise comparisons. **D** Upper: Representative H&E staining of pancreatic tumors in KPC and KPNiC at HEP. Scale bar: 100 μm. Lower left: Cancer grading distribution (G2 vs. G3) in KPC and KPNiC tumors. Lower right: Quantification of remodeling area (%) of KPC and KPNiC pancreata. *N* ≥ 8 mice/group. t = HEP. Unpaired Student’s *t* test. **E** Kaplan–Meier survival analysis comparing overall survival between KPC and KPNiC mice. Median survival was 85 days for KPC and 73.5 days for KPNiC mice. Log-rank test. **F** Left: Representative H&E staining of pancreatic tissue from KPC and KPNiC at 8 weeks. Lower panels show magnified views of boxed areas. Scale bar: 100 μm. Middle: Percentage of mice with established cancer. KPC: 25% cancer (*N* = 8), KPNiC: 83.3% cancer (*N* = 6). Right: Quantification of remodeling area (%) in KPC and KPNiC mice. *N* = 6 mice/group. *t* = 8 weeks. Unpaired Student’s *t* test. Dot plots: Dots represent individual animals. Data are presented as mean ± SD. n.s.: *p* > 0.05; *p < 0.05; **p < 0.01; ****p < 0.0001.

To explore if NIK (encoded by *MAP3K14*) is deregulated in human pancreatic cancer, we analyzed patient survival data from TCGA and GTEx databases. Patients with low *MAP3K14* transcript levels exhibited significantly reduced overall survival compared to those with higher expression (Fig. 1A). This correlation prompted us to investigate its role in PDAC development and progression.

We then utilized pancreas-specific NIK-proficient mice (WT) and NIK-deficient mice carrying the Pdx1-Cre; NIK^fl/fl^ genotype (NiC). Genotyping confirmed efficient *Map3k14* recombination, as evidenced by the absence of the wild-type band (~4 kb) in NiC pancreata (Fig. S1A). RNAscope analysis further showed markedly reduced *Map3k14*^*+*^ signal in NiC pancreata (Fig. 1B). Although NIK is primarily regulated at the post-translational level through constitutive ubiquitination and proteasomal degradation, the reduction in *Map3k14* mRNA in our model reflects deletion of the gene and is therefore expected to result in loss of NIK protein expression [11].

Having confirmed efficient NIK ablation in the pancreas, we next tested whether loss of NIK dampens non-canonical NF-κB signaling in the context of oncogenic KRAS, by examining RelB localization. Nuclear translocation of RelB occurred frequently in NIK-proficient KRAS^G12D^ pancreata, whereas it remained largely cytoplasmic in the absence of NIK, indicating that the pathway was effectively suppressed (Fig. 1C). Consistently, nuclear fractionation of KPC and KPNiC cancer cells followed by Western blot analysis confirmed a significant reduction in nuclear RelB levels in NIK-deficient cells (Fig. S1B).

To assess the functional consequences of NIK loss on tumor progression, we generated KPC (Pdx1-Cre; KRAS^G12D^; p53^fl/fl^) and KPNiC (Pdx1-Cre; KRAS^G12D^; NIK^fl/fl^; p53^fl/fl^) mice, thereby combining oncogenic KRAS activation, p53 loss and NIK deletion (Fig. S1C). Cohorts were analyzed at 8 weeks of age and at the humane endpoint (HEP). Starting our analysis by examining mice at their HEP, pancreas-to-body weight ratios were comparable between groups, indicating that NIK deletion did not substantially affect overall tumor mass (Fig. S1D). Histological evaluation showed that tumors in both KPC and KPNiC mice were predominantly poorly differentiated (G3), with a minority displaying moderate differentiation (G2) (Fig. 1D).

Despite similar tumor burden and histological grade at HEP, Kaplan–Meier survival analysis revealed a significant reduction in survival in KPNiC mice (median 73.5 days for KPNiC vs. 85 days for KPC; Fig. 1E). These in vivo findings align with the human survival data, supporting a role for NIK in modulating PDAC prognosis.

To determine whether the reduced survival observed in KPNiC mice reflected earlier disease onset or increased aggressiveness, we analyzed tumor development at 8 weeks of age, a time point before the HEP.

At this stage, invasive PDAC was detected in 83.3% of KPNiC mice compared to only 25% of KPC mice (Fig. 1F), indicating a markedly accelerated progression in the absence of NIK. Consistently, the pancreas-to-body weight ratio was significantly elevated in KPNiC mice (Fig. S1E), reflecting greater tumor burden at this early time point.

Further histological analysis revealed expanded tumor-associated remodeling areas in KPNiC pancreata, characterized by dense desmoplastic stroma, inflammatory infiltrates (Fig. 1F), and proliferative epithelial cells. Ki67 staining of CK19^+^ ductal cells showed a significantly higher proliferation rate in KPNiC lesions compared to KPC controls (Fig. S1F), indicative of more aggressive neoplastic growth. Sirius Red staining further confirmed enhanced fibrosis in the KPNiC pancreas (Fig. S1G). CD45 immunohistochemistry demonstrated a trend toward increased immune cell infiltration in KPNiC mice at 8 weeks, although the difference did not reach statistical significance (Fig. S1H).

These results suggest that NIK deletion accelerates the transition from precancerous lesions to invasive PDAC, in part through increased cellular proliferation and enhanced stromal remodeling.

### NIK loss fosters a myCAF-enriched microenvironment

To evaluate whether NIK deletion contributes to increased invasiveness or metastatic spread, we examined KPC and KPNiC mice at the HEP for local invasion, distant metastases (liver and lung), and ascites. Although a slightly higher number of metastatic cases was observed in the livers of KPC mice, as well as increased ascites formation and invasion, these differences did not reach statistical significance (Fig. S2A). We hypothesize that since KPC mice exhibited a longer survival, this time window allowed a small increase (non-significant) in the number of metastatic cases. All these results suggest that NIK loss does not promote metastatic dissemination within the observation window of these models.

**Fig. 2:**
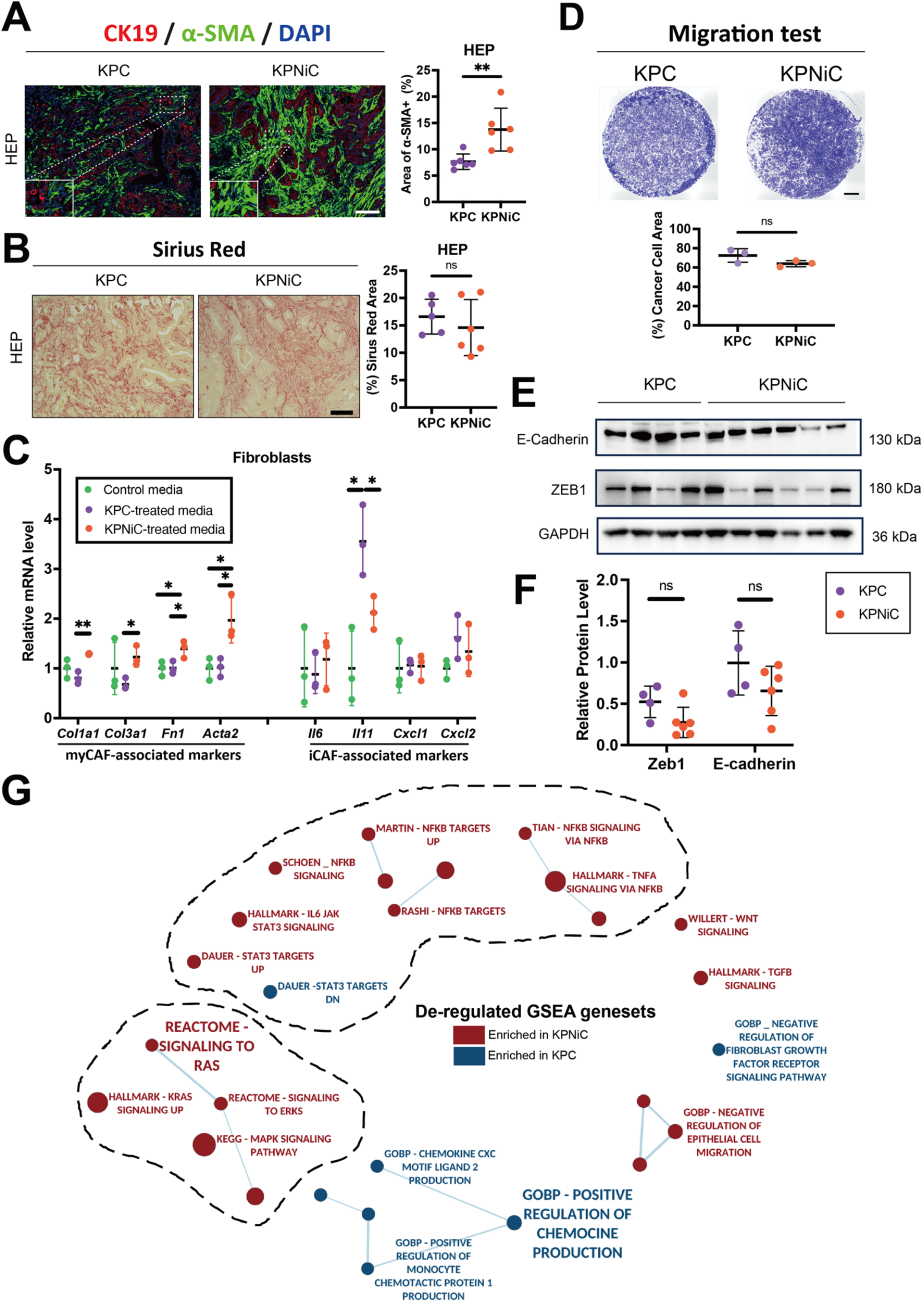
NIK loss fosters a myCAF-enriched microenvironment. **A** Left: Immunofluorescence staining for CK19 (red), α-SMA (green), and DAPI (blue) in KPC and KPNiC pancreata sections. Right: Quantification of α-SMA⁺ area (%). N = 6 mice/group. t = HEP. Unpaired Student’s *t* test. Scale bar: 100 μm. **B** Left: Representative Sirius Red staining for collagen deposition from KPC and KPNiC pancreas. Scale bar: 100 μm. Right: Quantification of Sirius Red⁺ area (%). *N* = 6 mice/group. t=HEP. Unpaired Student’s *t* test. **C** Expression levels of the indicated CAF-related genes in primary fibroblasts exposed to KPC- or KPNiC-derived conditioned media were quantified by qRT-PCR, with transcripts first normalized to the *Rpl13* reference gene and then expressed relative to the control-medium group. *N* = 3 biological replicates. One-way ANOVA with Bonferroni post hoc. **D** Top: Representative images of transwell migration assay using primary cancer cells from KPC and KPNiC mice. Scale bar: 1000 μm. Bottom: Quantification of area covered by migrated cells. *N* ≥ 3 biological replicates. Unpaired Student’s *t* test. **E** Western blot analysis of E-cadherin and ZEB1 in primary tumor cells from KPC and KPNiC mice. GAPDH serves as a loading control. **F** Quantification of Western blot band intensity for E-cadherin and ZEB1, normalized to GAPDH. *N* ≥ 4 biological replicates. Unpaired Student’s *t* test. **G** Enrichment map of GSEA gene sets differentially regulated in isolated KPC vs KPNiC cancer cells. Red: gene sets enriched in KPNiC; blue: gene sets enriched in KPC. Node size reflects gene set size; edge thickness reflects overlap. Dot plots: Dots represent individual animals. Data are presented as mean ± SD. ns: *p* > 0.05, *p < 0.05, **p < 0.01.

We next investigated whether NIK deletion altered the tumor microenvironment. Although Sirius Red staining at endpoint did not reveal significant differences in overall collagen deposition, α-smooth muscle actin (α-SMA) staining was significantly elevated in KPNiC tumors (Fig. 2A, B), suggesting increased presence of activated myofibroblastic cancer-associated fibroblasts (myCAFs). This prompted us to further characterize phenotypic differences of fibroblasts.

Accordingly, we isolated primary tumor cells from KPC and KPNiC tumors, cultured them and used their conditioned media to stimulate pancreatic stellate cells (PSCs) (Fig. S2B). PSCs treated with KPNiC-conditioned media exhibited upregulation of myCAF-associated genes (*Col1a1, Col3a1, Fn1, Acta2*), while expression of inflammatory CAF (iCAF) markers (*Il6, Il11, Cxcl1, Cxcl2*) was not significantly affected (Fig. 2C). These data, together with increased α-SMA staining in vivo, indicate that NIK loss skews the tumor microenvironment toward a myCAF-enriched phenotype.

To determine whether conditioned media derived from KPC or KPNiC tumors affects fibroblast proliferation or induces senescence, we analyzed fibroblast growth kinetics following treatment. Growth curve analysis did not reveal significant differences between KPC- and KPNiC-conditioned media (Fig. S2C). We next assessed the expression of senescence-associated related genes. Although minor variations were observed, no consistent or robust differences were detected (Fig. S2D). Finally, senescence induction was evaluated by SA-β-gal (X-Gal) staining, which showed no significant difference in the frequency of senescent cells between treatment groups (Fig. S2E). Collectively, these results indicate that conditioned media from KPNiC tumors does not significantly alter fibroblast proliferation or induce a senescence phenotype under our experimental conditions.

To assess whether NIK loss intrinsically alters tumor cell behavior, we compared the migratory capacity of KPC- and KPNiC-derived primary tumor cells using transwell assays (Fig. S2B). No significant differences were observed, although KPC cells displayed a trend toward increased migration (Fig. 2D). In addition, Western blot analysis of epithelial–mesenchymal transition (EMT) markers revealed no significant difference in E-cadherin and ZEB1 (Fig. 2E and F). These data suggest that NIK deletion does not enhance the invasiveness of pancreatic cancer cells.

To explore transcriptional changes associated with NIK loss, we performed bulk RNA sequencing on tumor cells isolated from KPC and KPNiC mice. Gene set enrichment analysis (GSEA) revealed a significant upregulation of canonical NF-κB signaling in KPNiC cells (Fig. 2G and S3A). Crosstalk between canonical and non-canonical NF-κB pathways has been reported to be context- and disease-dependent, with evidence supporting both cooperative and competitive interactions [12–14]. Notably, Western blot analysis of nuclear extracts did not show increased nuclear RelA/p65 levels in KPNiC cells (Fig. S3B). This indicates that enhanced canonical target gene expression may occur without a detectable increase in RelA nuclear abundance, potentially reflecting reduced competition with RelB at κB binding sites. Given the established role of canonical NF-κB in promoting inflammation, fibrosis, and disease progression in PDAC, this pathway shift may contribute to the rapid tumor development observed in NIK-deficient mice.

Further analysis of the RNA-seq data revealed enrichment of TGF-β, Wnt, and FGF signaling gene sets in KPNiC cells, which are pathways linked to fibroblast activation and the establishment of a myCAF phenotype (Figs. S3C and S3D). Specifically, TGF-β was shown to drive myCAF differentiation via SMAD signaling in fibroblasts [15], Wnt signaling was shown to contribute to fibroblast reprogramming and suppress iCAF-associated programs [16], and FGF signaling was shown to support fibroblast expansion and extracellular matrix (ECM) production [17], complementing our in vitro data. In addition, enrichment of MAPK and IL6/STAT3 signaling pathways, both associated with tumor progression, was observed, further supporting a pro-tumorigenic signaling landscape in NIK-deficient tumors (Fig. S3E and S3F) [18].

Taken together, these transcriptomic changes, alongside the increased α-SMA staining and the upregulation of myCAF-associated genes in PSCs, strongly support the emergence of a pro-myCAF tumor microenvironment in the absence of NIK. These findings indicate that NIK loss reshapes tumor-stroma interactions not by altering tumor cell invasiveness, but by promoting paracrine signaling programs that activate and polarize cancer-associated fibroblasts toward a fibrotic, myCAF-enriched state.

### NIK deletion accelerates lesion formation and progression in the KC model

KPNiC mice exhibited a median survival of approximately 10 weeks, which limited the opportunity to analyze intermediate stages of tumorigenesis. To facilitate a more gradual and controlled evaluation of lesion development, we utilized NiC, Pdx1-Cre; KRAS^G12D^ (KC), and Pdx1-Cre; KRAS^G12D^; NIK^fl/fl^ (KNiC) mice as complementary models. In contrast to p53-deficient models, KC mice retain functional p53, resulting in slower progression toward high-grade lesions and invasive PDAC.

**Fig. 3:**
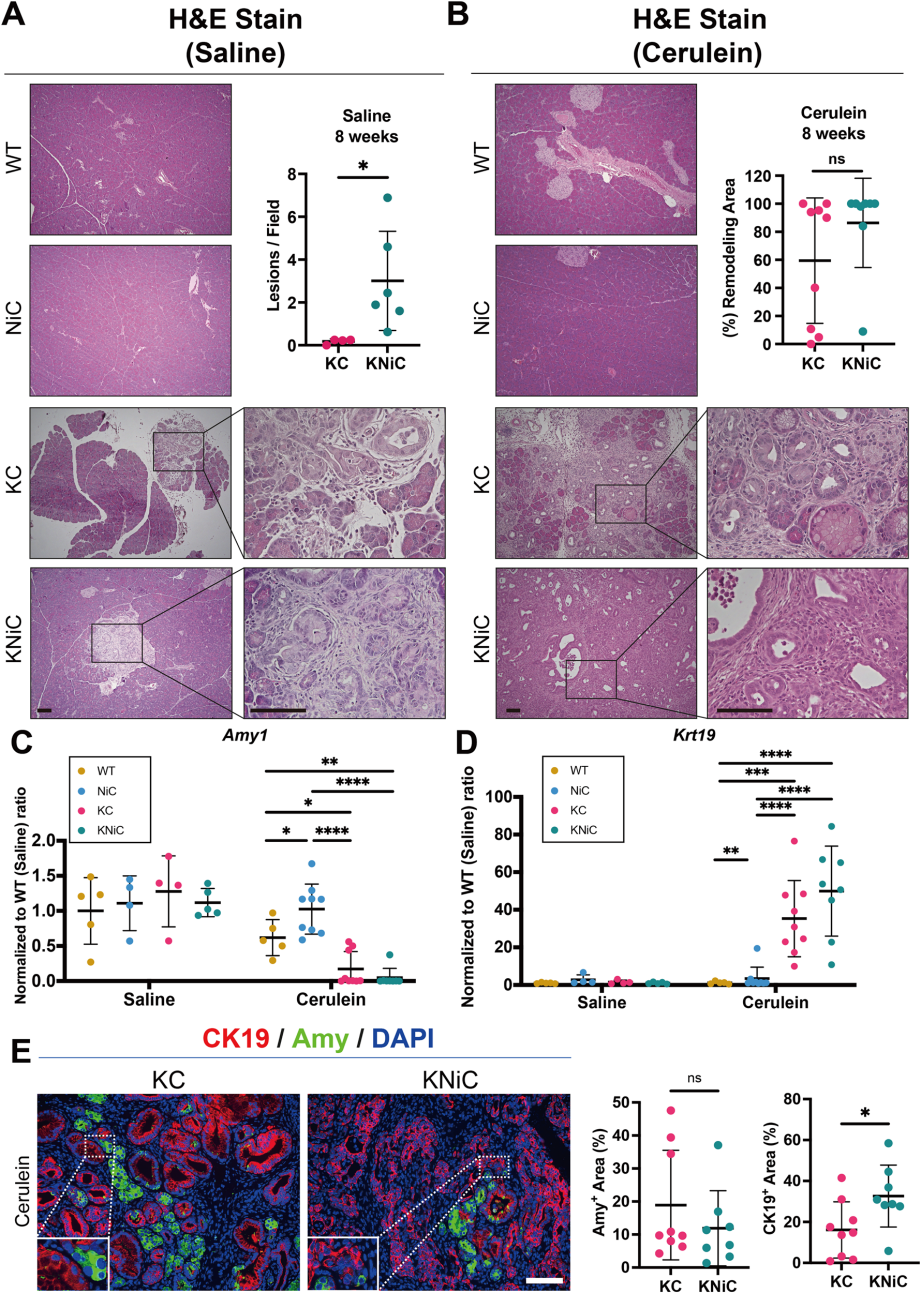
NIK deletion accelerates lesion formation and progression in the KC model. **A** Representative H&E staining of pancreatic tissue from saline-injected WT, NiC, KC, and KNiC mice. Bottom panels show magnified views of boxed regions. Upper Right: Quantification of low-grade PanIN lesions per field. *N* ≥ 4 mice/group. *t* = 8 weeks. Welch’s t test. **B** Representative H&E staining of pancreatic sections from cerulein-injected WT, NiC, KC, and KNiC mice. Bottom panels show magnified views. Upper Right: Quantification of remodeling area (%). *N* ≥ 6 mice/group. *t* = 8 weeks, Mann–Whitney U test. **C** Quantification of ***Amy*** mRNA expression level by qRT-PCR in pancreata from saline- and cerulein-injected mice. Expression was first normalized to the *Rpl13* reference gene and then expressed relative to the WT-saline control. *N* ≥ 4 mice/group. *t* = 8 weeks. Two-way ANOVA with Tukey’s post hoc. **D** Quantification of ***Krt19*** mRNA expression level by qRT-PCR in the same samples as (**C**). Expression was first normalized to the *Rpl13* reference gene and then expressed relative to the WT-saline control. *N* ≥ 4 mice/group. *t* = 8 weeks. Two-way ANOVA with Tukey’s post hoc. **E** Left: Immunofluorescence staining for Amylase and CK19 on pancreata from 8-week-old cerulein-injected KC and KNiC mice. Scale bar: 100 μm. Right: Quantification of the Amy^+^ and CK19^+^ area. *N* ≥ 8 mice/group. *t* = 8 weeks, unpaired Student’s *t* test. Dot plots: Dots represent individual animals. Data are presented as mean ± SD. Scale bars: 100 μm. ns: *p* > 0.05, *p < 0.05, **p < 0.01, ***p < 0.001, ****p < 0.0001.

To mimic inflammatory conditions associated with human pancreatic disease, 6-week-old mice received intraperitoneal injections of cerulein or saline (control), and pancreata were collected at 8 weeks of age (Fig. S4A). In saline-treated cohorts, WT and NiC mice exhibited histologically normal pancreata (Fig. 3A). In contrast, both KC and KNiC mice developed low-grade lesions, with KNiC pancreata displaying a significantly higher lesion burden relative to KC controls. This was accompanied by a modest, though non-significant, increase in the pancreas-to-body weight ratio in KNiC mice (Fig. S4B). These results suggest that NIK deletion enhances early neoplastic transformation in the presence of oncogenic KRAS.

Histological analysis of cerulein-treated mice revealed a similar trend. WT and NiC pancreata remained largely intact, whereas both KC and KNiC mice exhibited extensive tissue remodeling (59.45% for KC vs 86.37% for KNiC, Fig. 3B). Nevertheless, overall remodeling burden did not differ significantly between cohorts, and endocrine function, as primarily indicated by blood glucose levels, remained unchanged (Fig. S4C).

Notably, histopathological grading revealed a shift in lesion severity. KC pancreata were enriched for low-grade PanINs, while KNiC pancreata exhibited a higher proportion of high-grade lesions. Furthermore, invasive carcinoma was identified in 44% of KC mice, compared to 75% in KNiC mice (Fig. S4D). Taken together, these findings indicate that NIK deletion is associated with accelerated lesion formation and a higher prevalence of advanced lesions. While this is consistent with enhanced progression toward malignancy, we cannot exclude that the higher prevalence of advanced lesions is secondary to earlier lesion initiation and accelerated PDAC development. In this scenario, earlier cancer establishment could promote a pro-inflammatory microenvironment, for example through increased cytokine secretion, which may further drive lesion progression.

In support of potential translational relevance, NIK protein expression was analyzed in a human PDAC tissue microarray (*n* = 20) and correlated with overall survival. Higher NIK expression was associated with a trend toward improved survival. While the association did not reach statistical significance, the directionality is consistent with our experimental findings and supports validation in larger independent cohorts (Fig. S4E).

Molecular analyses supported these histological findings. In cerulein-treated mice, KNiC pancreata exhibited elevated expression of *Krt19* (CK19), a ductal marker associated with neoplastic transformation (Fig. 3C). Expression of *Amy*, a marker of acinar differentiation, was reduced following cerulein treatment in both KC and KNiC mice, without a significant difference between the two groups (Fig. 3D). Immunofluorescence staining confirmed these findings at the protein level, demonstrating significantly increased CK19⁺ areas in KNiC pancreata, while Amylase⁺ areas were comparably reduced in both groups (Fig. 3E). This transcriptional shift is consistent with enhanced ductal reprogramming in the absence of NIK. In line with increased tumor burden, KNiC mice also exhibited a significantly elevated pancreas-to-body weight ratio following cerulein treatment (Fig. S4B).

### NIK deletion enhances proliferation and modulates acinar cell survival in the pancreas

**Fig. 4:**
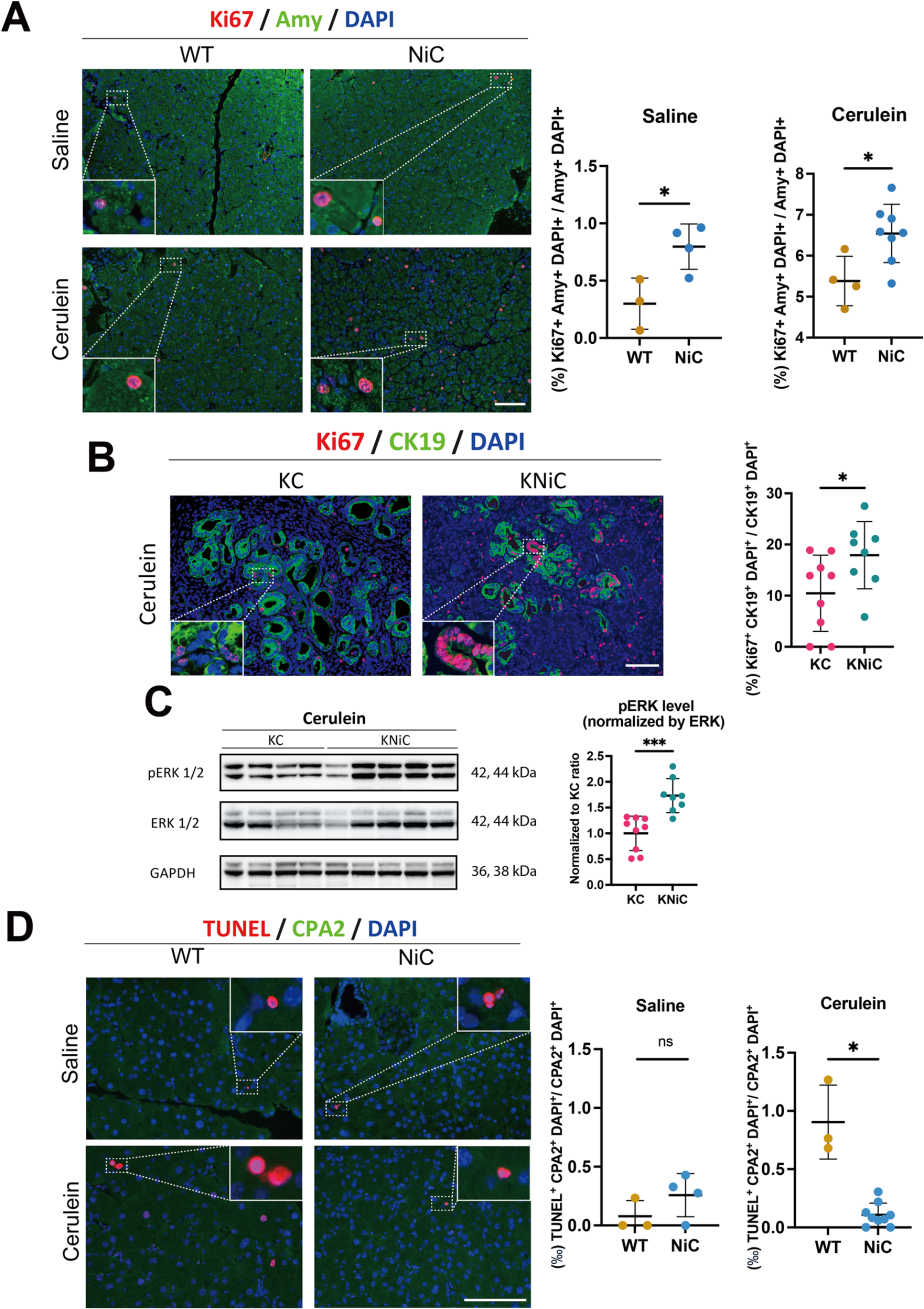
NIK deletion enhances proliferation and modulates acinar cell survival in the pancreas. **A** Left: Representative Ki67 (red) and Amy (green) staining in WT and NiC mice under saline or cerulein conditions. Right: Quantification of the proportion of Ki67⁺ cells among total amylase (Amy)⁺ cells in saline or cerulein groups. *N* ≥ 3 mice/group. *t* = 8 weeks. **B** Left: Immunofluorescence staining of pancreatic sections from cerulein-injected KC and KNiC mice for Ki67 (red), CK19 (green), and DAPI (blue). Right: Quantification of Ki67⁺/CK19⁺/DAPI⁺ cells as a percentage of total CK19⁺/DAPI⁺ cells. *N* ≥ 8 mice/group. *t* = 8 weeks. **C** Left: Western blot analysis of phosphorylated ERK1/2 (pERK1/2) and total ERK1/2 in pancreata from cerulein-injected KC and KNiC mice. GAPDH serves as a loading control. Right: Quantification of pERK normalized to total ERK. *N* ≥ 8 mice/group. *t* = 8 weeks. **D** Left: Representative images of TUNEL (red) and CPA2 (green) co-staining in WT and NiC mice under saline or cerulein treatment. Right: Quantification of the proportion (‰) of TUNEL⁺ cells among total CPA2⁺ acinar cells in saline and cerulein groups. *N* ≥ 3 mice/group. *t* = 8 weeks. Dot plots: Each dot represents an individual animal. Data are presented as mean ± SD. Scale bar: 100 μm. *p < 0.05, ***p < 0.001, unpaired Student’s *t* test.

The non-canonical NF-κB pathway has been implicated in promoting proliferation across various cell types [19–22]. To determine whether NIK deletion affects proliferative capacity in the pancreas, we analyzed saline-injected WT and NiC pancreata using Ki67 and α-amylase co-staining. Notably, NiC mice exhibited a significant increase in Ki67-positive acinar cells, indicating enhanced proliferation in the absence of NIK (Fig. 4A).

This proliferative phenotype was sustained following cerulein-induced pancreatitis, likely reflecting a persistent effect of NIK loss and regenerative signals induced by injury (Fig. 4A). Within cerulein-injected KC and KNiC mice, proliferation was specifically elevated within CK19^+^ ductal cells upon NIK deletion (Fig. 4B). This pattern aligns with the histological observations of more advanced PanIN lesions in KNiC mice, given that high-grade PanINs are known to exhibit elevated proliferation relative to low-grade lesions.

Given the central role of the MAPK signaling cascade in PDAC progression and cell proliferation [23], we next evaluated pathway activation by assessing ERK phosphorylation. WB analysis revealed increased levels of phosphorylated ERK in KNiC pancreata compared to KC controls, indicating enhanced MAPK pathway activation in the context of NIK loss and oncogenic KRAS (Fig. 4C).

To further examine pathway regulation due to NIK loss and oncogenic KRAS, we isolated pancreata from saline-injected KC and KNiC mice and performed bulk RNA-seq. GSEA revealed depletion of gene sets associated with non-canonical NF-κB pathway in NIK-deficient tissue, fitting our mouse model and hypothesis (Fig. S5A). Importantly, we observed significant enrichment of the KRAS-MAPK signaling gene set in KNiC samples (NES = 1.593, FDR = 0.0123; Fig. S5B), further supporting our WB results about augmented MAPK signaling following NIK deletion.

Since cerulein-induced pancreatitis is associated with widespread acinar cell death, we assessed whether NIK influences acinar cell survival. TUNEL staining showed extensive acinar (CPA2⁺) cell loss in cerulein-treated WT pancreata, whereas NiC mice displayed fewer TUNEL-positive acinar cells (Fig. 4D). In contrast, no significant differences in TUNEL-positive cells were observed between cerulein-treated KC and KNiC mice (Fig. S5C).

Cleaved caspase-3 staining revealed only rare positive cells and did not differ between WT/NiC or KC/KNiC groups (Fig. S5D and S5E). The lower frequency of cleaved caspase-3⁺ cells compared to TUNEL positivity is consistent with the transient nature of caspase-dependent apoptosis and the broader detection of DNA fragmentation by TUNEL. Together, these findings indicate that NIK deletion confers partial protection from injury-induced cell death in the non-neoplastic setting, an effect that is not evident in oncogenic KRAS-driven pancreata.

To assess whether NIK deletion influences oncogene-induced senescence (OIS), we evaluated senescence markers in vivo and in vitro. X-Gal staining and RNA-seq-based GSEA analysis revealed no significant differences between KC and KNiC pancreata (Fig. S6A, S6B). While qPCR showed minor variations in selected transcripts, these are likely attributable to stromal or inflammatory components (Fig. S6C).

Similarly, analysis of KPC and KPNiC tumor cells under untreated conditions and following etoposide treatment showed comparable senescence induction in both groups (Fig. S6D). qPCR analysis of senescence-associated markers in untreated tumor cells likewise revealed no significant differences between genotypes (Fig. S6E). Collectively, these findings indicate that NIK deletion does not significantly alter senescence in pre-neoplastic or tumor settings.

### NIK deletion enhances stromal remodeling and immune cell infiltration in KRAS-driven PanINs

**Fig. 5:**
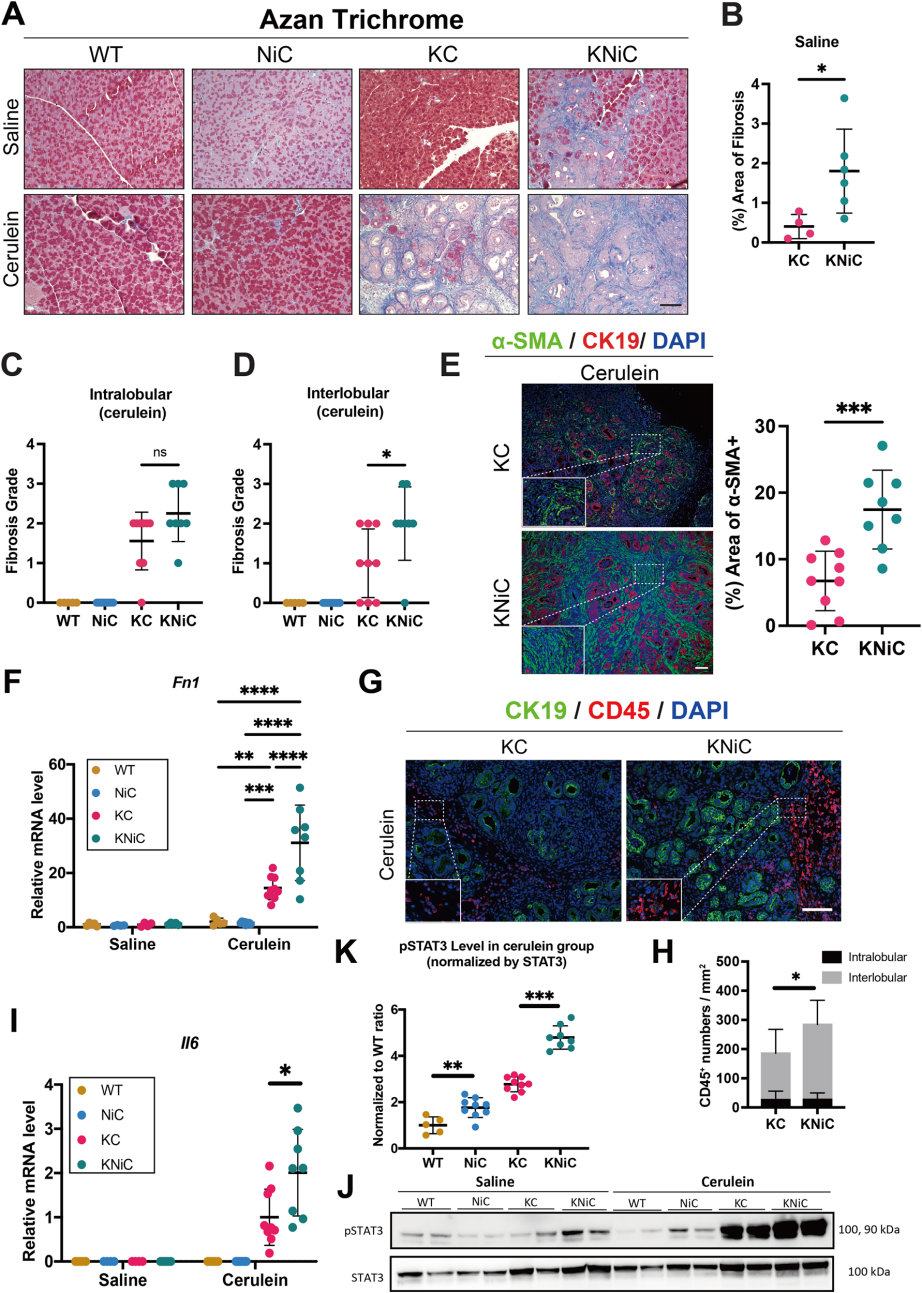
NIK deletion enhances stromal remodeling and immune cell infiltration in KRAS-driven PanINs. **A** Representative Azan Trichrome staining of WT, NiC, KC and KNiC mice treated with saline/cerulein (8 weeks). **B** Azan-positive area (%) in saline KC versus KNiC. *N* ≥ 4 mice/group. *t* = 8 weeks. Fibrosis grade in (**C**) intralobular and (**D**) interlobular regions in cerulein-treated WT, NiC, KC, and KNiC. *N* ≥ 5 mice/group. *t* = 8 weeks. Mann-Whitney U test. **E** Left: IF staining for CK19 (red)/α-SMA (green)/DAPI (blue) of cerulein-treated KC and KNiC. Right: Quantification of α-SMA-positive area (%). *N* ≥ 8 mice/group. *t* = 8 weeks. **F** mRNA expression level of ***Fn1*** in pancreata from WT, NiC, KC, and KNiC mice with saline or cerulein treatment, normalized to WT-saline. *N* ≥ 4 mice/group. *t* = 8 weeks. Two-way ANOVA with Tukey’s post hoc. **G** IF staining for CK19 (green)/CD45 (red)/DAPI (blue) of cerulein-treated KC and KNiC. **H** CD45⁺ immune cell density (cells/mm²) in intralobular and interlobular regions; only the interlobular compartment is elevated in KNiC. *N* ≥ 7 mice/group. *t* = 8 weeks. **I** mRNA expression level of ***Il6*** in pancreata from WT, NiC, KC, and KNiC mice treated with saline or cerulein. Data normalized to cerulein-treated KC group. *N* ≥ 4 mice/group. *t* = 8 weeks. Unpaired Student’s t test (KC cerulein vs KNiC cerulein). **J** Western blot analysis for phosphorylated STAT3 (pSTAT3) and total STAT3 proteins in representative pancreata from WT, NiC, KC, and KNiC saline or cerulein group. **K** Quantification of pSTAT3/STAT3 ratio, presented as ratio relative to WT cerulein group. *N* ≥ 5 mice/group. *t* = 8 weeks. Unpaired Student’s *t* test between groups (WT vs NiC, KC vs KNiC). Dot plots: Each dot represents an individual animal. Data are presented as mean ± SD. Gene expression level was first normalized to the *Rpl13* reference gene. Unpaired Student’s *t* test unless noted. n.s.: *p* > 0.05; *p < 0.05; **p < 0.01; ***p < 0.001; ****p < 0.0001. Scale bar: 100 μm.

Our findings so far indicate that NIK deletion, in the context of oncogenic KRAS, accelerates low-grade PanINs formation. Building on our earlier observation of pronounced stromal remodeling in KPNiC cancer, we asked whether a similar response already accompanies these earliest lesions. Accordingly, we performed Azan Trichrome and α-SMA staining on pancreata of saline-injected mice. KNiC mice exhibited more intense collagen deposition and a larger α-SMA-positive area relative to KC controls, suggesting that even early-stage PanIN formation is supported by an enhanced fibrotic response in the absence of NIK (Figs. 5A, B and S7A). GSEA revealed that both collagen synthesis and degradation programs were up-regulated, suggesting that enhanced ECM remodeling activity may contribute to the fibrotic phenotype (Fig. S7B).

To further characterize this effect under conditions of cerulein-induced inflammation, we quantified collagen deposition in cerulein-injected KC and KNiC mice. Fibrosis was stratified into two distinct compartments: interlobular fibrosis (between acinar lobes/lesions areas) and intralobular fibrosis (within acinar lobes/lesion areas). While intralobular fibrosis did not differ significantly between groups, interlobular fibrosis was markedly elevated in KNiC mice, suggesting enhanced activation of pancreatic stromal cells in the interlobular region (Fig. 5A, C, D).

To determine whether this fibrotic expansion was associated with activation of pancreatic stellate cells (PSCs) and CAFs, we assessed transcriptional levels of *Fn1* and protein expression of α-SMA, a marker of myCAFs, as well as Vimentin, a mesenchymal stromal marker (Figs. 5E, F, S7C) [24, 25]. Consistent with our findings in the p53-deficient cohort, NIK deletion led to a significant upregulation of *Fn1*, α-SMA and Vimentin, indicating increased stromal cell activation in the tumor microenvironment.

The spatial expansion of activated PSCs/CAFs can impede immune cell infiltration. Therefore, we next investigated immune cell density. Quantification of CD45^+^ cells revealed a higher total number of infiltrating immune cells in KNiC mice compared to KC controls (Figs. 5G, H and S7D). Spatial analysis of immune cell localization showed a significant increase in CD45^+^ cells in the interlobular region, while the intralobular compartment remained unchanged. These findings suggest that, despite enhanced immune infiltration overall, only a subset of immune cells successfully penetrates the lesion core in KNiC mice.

To explore chemoattracting mechanism driving increased immune infiltration, we examined the IL6–STAT3 signaling axis, which lies downstream of canonical NF-κB and has been associated with immunomodulation in pancreatic cancer [26, 27]. In KNiC pancreata, we observed elevated *Il6* mRNA transcription and increased STAT3 activation compared to KC controls, indicating that NIK deletion indirectly activates the IL6–STAT3 pathway (Fig. 5I, J and K). This observation mirrors the pathway activation seen in the p53-deficient background (Fig. S3F).

Finally, we profiled specific immune cell subtypes to determine whether NIK deletion selectively affects immune composition. Immunohistochemical analysis revealed a significant increase in Ly6G-positive neutrophils in KNiC mice compared to KC controls, while no statistically significant differences were observed in B cells (B220^+^), T cells (CD3^+^), or macrophages (F4/80^+^) (Fig. S7E). These findings suggest that NIK deletion specifically enhances neutrophil infiltration, which may contribute to the remodeling of the tumor microenvironment observed in KNiC mice.

## Discussion

The NF-κB signaling pathway is a key regulator of inflammation, fibrosis, and immune responses in pancreatic cancer [28]. While the canonical pathway, regulated via RelA/p65, has been extensively investigated in PDAC progression, the role of the non-canonical NF-κB pathway remains less well understood. Studies have suggested tumor-promoting functions for this pathway; for example, loss of NFKB2 delayed PanIN development and reduced early tumor formation [29], and pancreas-specific deletion of *Relb* suppressed tumor formation in the KC model [30]. Consistent with a tumor-promoting role in certain contexts, NIK has been shown to drive melanoma growth and invasion, supporting a pro-tumorigenic function of non-canonical NF-κB signaling [31]. However, the role of NIK appears to be highly context- and tissue-dependent. In lung cancer, NIK was reported to have no significant impact on tumor progression [32], indicating cancer-type-specific differences. Finally, a tumor-suppressive role for this pathway has been reported in other malignancies including colorectal cancer and AML, where disruption of NIK–RelB signaling impairs differentiation and accelerates tumorigenesis [33, 34].

In our study, we show that deletion of NIK, a central kinase of the non-canonical NF-κB pathway, accelerates PDAC progression and reduces survival, thereby indicating a tumor-suppressive function for NIK in KRAS-driven pancreatic cancer. This finding challenges the prevailing assumption that NF-κB signaling promotes pancreatic tumorigenesis and identifies NIK as a regulator of early disease progression and tumor–stroma dynamics.

Publicly available patient datasets suggest that *MAP3K14* expression levels may carry prognostic value in PDAC, with lower expression linked to worse outcomes. To explore this in vivo, we deleted *Map3k14* in the pancreas of KPC mice (named KPNiC mice), which resulted in significantly shorter survival despite similar tumor grade and metastasis development compared to KPC. However, given the significantly earlier mortality of KPNiC mice, it remains possible that macro-metastatic spread and ascites were not observed because there was insufficient time for these later-stage phenotypes to manifest. These findings suggest that NIK loss drives early tumor progression without necessarily altering the final histological state at endpoint, a concept supported by the increased tumor burden observed at 8 weeks.

NIK deletion significantly altered the tumor microenvironment. Conditioned media from KPNiC tumor cells induced myofibroblast-associated gene expression in pancreatic stellate cells, and α-SMA staining was elevated in vivo, indicating a shift toward a myCAF-enriched stroma. Although total fibrosis was not markedly different, RNA sequencing of tumor cells revealed activation of canonical NF-κB signaling and enrichment of TGF-β, Wnt, and FGF pathway signatures, all associated with fibroblast activation. In mice lacking NIK, nuclear RelB was markedly reduced, and GSEA confirmed the down-regulation of the non-canonical NF-κB program. With fewer RelB molecules occupying κB sites, RelA:p50 meets less competition, clearing the way for an amplified canonical NF-κB response [12, 35]. Therefore, our data suggest that disruption of non-canonical NF-κB signaling contributes to a fibrotic tumor microenvironment, potentially through activation of canonical NF-κB–associated signaling.

Our results also indicate that NIK deletion affected early neoplastic transformation. In the oncogenic-KRAS p53-proficient model, KNiC mice developed more lesions and progressed faster to high-grade PanINs and invasive cancer, especially following cerulein-induced pancreatitis. These findings indicate that NIK loss accelerates both lesion formation and overall PDAC progression in the context of oncogenic KRAS. Associated molecular changes included elevated ERK activation, increased CK19-positive ductal proliferation, and reduced acinar cell numbers. Ki67 staining further revealed increased proliferative activity in the acinar compartment of KNiC mice compared to KC controls. Prior work has also shown that NIK suppresses epithelial turnover in the liver [20] and hematopoietic stem cell proliferation [21]. Notably, we also observed that NIK loss protected acinar cells from cerulein-induced cell death, suggesting that in the absence of NIK, surviving acinar cells may serve as a larger reservoir for ADM and PanIN initiation. This protective effect was no longer detectable once PanINs had formed, suggesting that it is specific to early-stage injury responses and not maintained during later neoplastic transformation.

NIK deletion also affected the immune landscape and contributed to changes in the tumor microenvironment in KNiC mice. Although overall fibrosis was comparable between genotypes, collagen deposition was significantly elevated in the interlobular regions of KNiC pancreata. These fibrotic areas were also enriched for CD45-positive immune cells, suggesting a spatial coupling of stromal remodeling and immune infiltration. Increased *Il6* transcription and elevated STAT3 activation in KNiC mice point to enhanced activity of the IL6–STAT3 signaling cascade, which is frequently associated with tumor-promoting inflammation and immune exclusion in PDAC [18]. These findings support the idea that NIK deletion promotes enhanced pro-inflammatory signaling via IL6 production.

In summary, our data reveal that NIK plays a tumor-suppressive role in KRAS-driven pancreatic cancer by restraining early lesion formation and modulating the fibrotic and immune microenvironment. In the absence of NIK, canonical NF-κB signaling is upregulated, leading to enhanced IL6–STAT3 activation, neutrophil recruitment, fibroblast polarization, and acinar cell survival, all of which contribute to a tumor-permissive niche. These observations are consistent with evidence that components of the NF-κB pathway, including NEMO [36] and RelA [37], can function in dual roles depending on disease stage and cellular context.

Conclusively, our findings indicate that NIK primarily regulates early tumor initiation and progression rather than late-stage disease behavior, as NIK deletion accelerated lesion formation and reduced survival without significantly affecting invasion or metastasis. These observations position MAP3K14 expression level as a potential prognostic marker in early PDAC and support further validation in larger independent patient cohorts.

## Methods

### Mice

*Pdx1-Cre* mice [38] were crossed with mice carrying a single *LSL-Kras*^*G12D*^ allele [39] and/or floxed *Map3k14* (NIK) alleles [11] to generate the experimental cohorts. Both sexes were used for the study. At 6 weeks, mice received intraperitoneal cerulein (50 μg/kg in 0.9% NaCl, Bachem #4030451, Bubendorf, Switzerland) following the schedule described by Tsesmelis et al. [36]. Control mice received 0.9% NaCl (Fresenius Kabi #PZN06605514, Bad Homburg, Germany) solution. Mice were analyzed at 8 weeks of age.

For the p53-deficient PDAC model, *Pdx1-Cre* transgenic mice were bred with mice harboring one *LSL-Kras*^*G12D*^ allele, *p53-LoxP* alleles [40] and/or floxed *Map3k14* alleles to obtain experimental mice, which were analyzed at 8 weeks or upon reaching humane endpoint (HEP) criteria identical to those described in our previous study [36]. Littermates lacking Cre expression served as wild-type (WT) controls. All mice were C57BL/6 background under standard housing conditions at the animal facility of the University of Ulm.

### Tissue processing and histology

Blood, pancreata, livers and lungs were collected. Organs were snap-frozen or fixed in 4% PFA. Pancreas-to-body weight ratio was recorded. Formalin-fixed paraffin-embedded (FFPE) sections (3 μm) were stained with hematoxylin and eosin (H&E), Azan Trichrome, and Sirius Red using established protocols [36]. For remodeling-area quantification, whole H&E sections were imaged and analyzed. For Azan Trichrome and Sirius Red staining, ≥5 randomly selected non-overlapping fields per sample were analyzed (Supplementary Table S1).

### Histopathological grading of pancreatic lesions

The remodeling area was defined as the region containing inflammation, fibrosis, precancerous and cancerous lesions based on H&E-stained sections. Histopathological evaluation was performed by a board-certified veterinary pathologist and her team (Dr. Katja Steiger and Thomas Metzler, Technical University of Munich). Precancerous lesions were classified, following the grading system of Basturk et al., as low-grade mouse PanIN (mPanIN-1/2) and high-grade mPanIN (mPanIN-3). ADM—duct-like structures composed of cuboidal to low-columnar, non-mucinous epithelial cells that replace acinar clusters—was analysed together with the other low-grade lesions. mPanIN-1 exhibited flat (1A) or papillary (1B) epithelial cells with supranuclear mucin and minimal atypia. mPanIN-2 showed nuclear stratification, crowding, partial loss of polarity, and mild to moderate atypia. mPanIN-3 displayed complex glandular structures with marked atypia, loss of polarity, frequent mitotic figures, and features consistent with carcinoma in situ. Additionally, atypical flat lesions (AFLs)—flat epithelium with pronounced nuclear atypia and fibrotic stroma—were identified as alternative high-risk precursors [41, 42].

For mice with invasive PDAC, tumors were graded based on the Consensus Report on Pathology of Genetically Engineered Mouse Models of Pancreatic Exocrine Cancer [43]: well-differentiated (G1) tumors form well-defined glands lined by cuboidal-to-columnar cells with basally oriented, uniform nuclei, minimal pleomorphism and rare, non-atypical mitoses; moderately differentiated (G2) tumors have a disorganized architecture with less distinct glands, larger irregular nucleoli, greater nuclear pleomorphism and more—occasionally atypical—mitoses; poorly differentiated (G3) tumors show small ill-defined glands, infiltrating single cells or solid sheets with scant mucin, pronounced pleomorphism, large irregular nucleoli and frequent atypical mitoses. All slides were coded and scored blind to genotype and treatment.

### Immunofluorescence (IF) and Immunohistochemistry (IHC)

FFPE sections were deparaffinized, subjected to antigen retrieval in citrate buffer (100 °C, 10 min), and blocked with 5% BSA containing Fc and Fab blockers for 2 h at room temperature. Sections were incubated with primary antibodies overnight at 4 °C. After PBS washes, fluorescent secondary antibodies and DAPI were applied for 1 h at room temperature, followed by mounting. A list of antibodies is presented in Supplementary Table S2. For IHC, antigen retrieval was followed by quenching of endogenous peroxidase activity with 3% hydrogen peroxide. Sections were then blocked, incubated with primary antibodies, followed by biotinylated secondary antibodies and streptavidin. Signal detection was performed using AEC^+^ chromogen. Apoptotic cells were detected using In Situ Cell Death Detection Kit (Roche #11684795910, Basel, Switzerland), and subsequently co-stained for CK19 and CPA2.

### Definition of interlobular and intralobular areas in the pancreas

Intralobular and interlobular stromal regions were manually defined based on histological architecture (Fig. S5C). The intralobular region lies within pancreatic lobules and contains primarily acinar cells, intercalated ducts, and limited stroma, where PanIN lesions are typically located. In contrast, the interlobular region is situated between lobules and comprises broader fibrotic areas with interlobular ducts, blood vessels, and nerve fibers. Intercalated ducts are lined by cuboidal epithelium, while interlobular ducts are lined by columnar epithelium and connect to the main pancreatic duct [36, 44]. Azan Trichrome staining was used to distinguish these regions, and structural features were validated using CK19 (ductal marker) and α-SMA (fibroblast marker).

### RNAscope in situ hybridization

RNAscope™ v2 (ACD Bio # 323110, Newark, USA) was performed on FFPE sections using a probe targeting *Map3k14* mRNA (ACD #1557721-C1). Slides underwent pretreatment, hybridization, amplification, and fluorescent detection following the manufacturer’s protocol. TSA Vivid 570 (1:1500) visualization was followed by DAPI counterstaining.

### Western Blot and qRT-PCR

Protein was extracted from snap-frozen tissue using SDS-based lysis buffer. Lysates were sonicated (40% amplitude; 3 × 7 s on/7 s off), resolved by SDS-PAGE, transferred (wet or semi-dry), and antibody detection was performed using enhanced chemiluminescence (ChemiDoc MP Imaging System, Bio-Rad, Hercules, USA). The list of antibodies used is provided in Supplementary Table S2. Total RNA was extracted from cells or tissues using RNeasy Mini Kit (Qiagen #74104, Düsseldorf, Germany), and cDNA was synthesized using Roche Transcriptor Kit (#5081955001). qRT-PCR was performed with SYBR Green detection on a LightCycler 480 II (Roche #05015278001). Relative expression was calculated according to *Rpl13*. The list of primers is provided in Supplementary Table S3.

### Fibroblast with conditioned media culture and transwell migration assay

Primary pancreatic cancer cells and pancreatic stellate cells (PSCs) were isolated following previously established protocols—cancer cells isolation according to Tsesmelis et al. [36] and PSCs isolation according to Chan et al. [45]. KPC and KPNiC cells were cultured 24 h in 10% FBS DMEM (Gibco #41965062, Grand Island, USA) until approximately 40% confluence, after which the supernatant was collected and passed through a 0.20 µm filter to yield conditioned media (CM). Fibroblasts were seeded at 500 cells/well in 24-well plates and cultured in CM diluted 2 : 3 with fresh 10% FBS DMEM. Every 24 h up to 96 h, wells were methanol-fixed, crystal-violet-stained, imaged, and the cell-covered area was quantified in ImageJ. For gene-expression analysis, fibroblasts were plated at 3 × 10⁵ cells/well in 6-well plates, grown overnight in DMEM, then treated with CM for 24 h before RNA extraction and qRT-PCR (primer sequences Table S3). Migration assays were performed by seeding 1 × 10⁵ KPC or KPNiC cells in 200 µL serum-free DMEM into 8 µm-pore Transwell inserts (Corning, #353097, Arizona, USA) and allowing migration toward 10% FBS DMEM (800 µL) for 24 h; membranes were crystal-violet-stained and the entire insert area imaged for quantification.

### Nuclear and cytoplasmic protein extraction

Primary cancer cells were harvested by trypsinization, washed with PBS, and centrifuged to obtain a dry cell pellet. Cytoplasmic and nuclear proteins were extracted using the NE-PER™ Nuclear and Cytoplasmic Extraction Reagents kit (ThermoFisher, #78835) according to the manufacturer’s instructions.

### X-Gal staining

Cryopreserved tissues, CM-treated fibroblasts, and isolated primary cancer cells were processed for X-Gal staining using the Senescence β-Galactosidase Staining Kit (Cell Signaling Technology, #9860S) according to the manufacturer΄s instructions. For fibroblast experiments, 5 × 10⁴ cells were seeded in 24-well plates and cultured overnight in DMEM. Cells were then treated with CM for 24 h prior to X-Gal staining. For induction of DNA damage in primary cancer cells, 3 × 10⁴ cells were seeded in 24-well plates. After 72 h, cells were treated with etoposide (MERCK, #E1383) at a final concentration of 500 nM for 4 h, following the protocol described by Tsesmelis et al [36].

### RNA sequencing and analysis

Total RNA was extracted from isolated pancreatic cancer cells (KPC, KPNiC, *n* = 3/group) and whole pancreatic tissues (saline-treated KC, KNiC, *n* ≥ 3/group) using the RNeasy Mini Kit. Poly-A, strand-specific libraries were sequenced on a DNBSEQ-G400 (paired-end 150 bp), yielding 23–25 million raw read pairs per sample; 92–96% were retained after filtering (Q20 > 95%, Q30 ≈ 88%). Low-quality reads and adapters were removed with SOAPnuke v2.3. Clean reads were aligned to the mouse reference genome GRCm39 (GCF_000001635.27) using HISAT2 v2.0.4, and gene-level counts were obtained with the RSEM module of the Dr. Tom™ pipeline. Differential expression was assessed in DESeq2 (adjusted *P* ≤ 0.05, |log₂FC | > 1). Gene set enrichment analysis (GSEA) was performed with GSEA software v4.4.0. Enrichment map was built using the CytoSCAPE software. Raw FASTQ files and normalized count matrices have been deposited in GEO under accession GSE301904.

### Human survival analysis from public datasets

Kaplan–Meier survival curves for *MAP3K14* expression in pancreatic adenocarcinoma (PAAD) were generated using GEPIA (http://gepia.cancer-pku.cn/), based on The Cancer Genome Atlas (TCGA) and Genotype-Tissue Expression (GTEx) data [46]. Patients were stratified into high- and low-expression groups based on the quartile cutoff, with the upper 25% classified as the high-expression group and the lower 25% as the low-expression group (cutoff-high = 75%, cutoff-low = 25%). Statistical significance was assessed using the log-rank test.

### Human tissue microarray and survival analysis

A human pancreatic adenocarcinoma tissue microarray (BioCat, #PA601) was stained using anti-NIK antibody and anti-CK7 antibody by IF. NIK protein expression in CK7+ lesions was determined. For consistency of paired analysis, 20 patients with ductal adenocarcinoma and matched cancer-adjacent tissues were included. NIK fluorescence intensity was quantified separately in cancer-adjacent and tumor core regions. The mean fluorescence intensity (MFI) of the cancer-adjacent areas was calculated and used as the cut-off value. Tumor core samples with MFI below this mean were classified as NIK-low, whereas those above were classified as NIK-high. Patients were stratified into high and low NIK groups according to tumor core MFI. Kaplan-Meier survival curves were generated based on lifespan data. Statistical significance was assessed using the log-rank test and the Gehan-Breslow-Wilcoxon test.

### Statistics

For the KC and KNiC cohorts, sample size estimation was based on expected effect sizes derived from preliminary observations and comparable studies. For saline-treated groups, an effect size of 0.83 (α = 0.025, power = 0.8) resulted in a target sample size of *n* = 5 animals per group, whereas an effect size of 0.49 yielded *n* = 12 animals per group for cerulein-treated groups. For the KPC and KPNiC cohorts, using the same α and power parameters, effect sizes of 0.563 and 0.508 resulted in target sample sizes of *n* = 10 animals per group (8-week cohort) and n = 12 animals per group (HEP cohort), respectively. The exact numbers of animals used was determined by genotype availability and the downstream experimental application. Blinding was not applicable during animal allocation, as all animals with the desired genotypes were included after genotyping. However, stratified randomization was applied to maintain comparable sex distribution across experimental groups. No inclusion or exclusion criteria were applied, and no animals were excluded. For comparisons between two groups with equal variance and normally distributed data, the unpaired Student’s t-test was used. When variance was unequal, Welch’s t-test was applied. For non-normally distributed data, the Mann–Whitney U test was used. For comparisons involving more than two groups and one independent variable, one-way ANOVA followed by Bonferroni’s post hoc test was used if variances were homogeneous. If variances were unequal, Welch’s ANOVA was applied. For experiments involving two independent variables, two-way ANOVA followed by Tukey’s post hoc test was used. Categorical data were analyzed using the Chi-square test. For the quantification of nuclear RelB⁺ cells, a Monte Carlo resampling approach was applied to account for field-level variability. For each WT mouse, 1000 random resampling iterations were performed to generate a null distribution of nuclear RelB counts across analyzed fields. Nuclear RelB counts from each NIK-deficient mouse were then compared against the corresponding WT-derived distributions to assess statistical significance. Survival curves were generated using the Kaplan–Meier method and compared using the log-rank test. Data are mean ± SD unless stated. All tests were two-tailed. Statistical significance was defined as follows: ns: *p* > 0.05, *p < 0.05, **p < 0.01, ***p < 0.001, ****p < 0.0001.

## Supplementary information

Supplementary Information

uncropped gels and blots

## Data Availability

The RNA-seq data supporting the findings of this study have been deposited in the GEO repository under accession number GSE301904 (https://www.ncbi.nlm.nih.gov/geo/query/acc.cgi?acc=GSE301904). The dataset includes both raw FASTQ files and processed read counts/TPM tables. Data will be made public upon manuscript publication or upon request. All other relevant data are available from the corresponding author upon reasonable request.
