# Supplementary material for: NF-κB inducing kinase (NIK) deletion accelerates KRAS-driven pancreatic cancer in association with tumor microenvironment remodeling
Source: Cell Death Dis. 2026 May 27;17(1):513. doi: 10.1038/s41419-026-08877-w (PMC13216544; doi:10.1038/s41419-026-08877-w)
Supplement: Supplementary file 1 — Supplementary Information [file 41419_2026_8877_MOESM1_ESM.pdf]

SUPPLEMENTARY INFORMATION

NF-κB inducing kinase (NIK) deletion accelerates KRAS-driven pancreatic cancer in association with tumor microenvironment remodeling

Running title: NF-κB inducing kinase deletion in pancreatic cancer

Ziwei Du [1], Ulrike F.G. Büttner [1], Hannah Lea Wirth [1], Melanie Gerstenlauer [1], Uta Manfras [1], Rikarda Loidl [1], Doğa Bahçeci [1], Katja Steiger [2,3], Thomas Metzler [2,3], Lap Kwan Chan [4], Miltiadis Tsesmelis [5 #, *], Thomas Wirth [1, #, *]

[1] Institute of Physiological Chemistry, University of Ulm, 89081, Ulm, Baden-Württemberg, Germany

[2] Institute of Pathology, Technical University Munich, 81675, Munich, Bayern, Germany

[3] Comparative Experimental Pathology (CEP), School of Medicine and Health, Technical University Munich, 81675, Munich, Bayern, Germany

[4] Department of Pathology and Molecular Pathology, University Hospital of Zurich, 8091, Zurich, Switzerland

[5] Single-Cell Sequencing Unit, University of Ulm, 89081, Ulm, Baden-Württemberg, Germany

[#] Authors share senior authorship

* Correspondence: Miltiadis Tsesmelis: miltiadis.tsesmelis@uni-ulm.de; Thomas Wirth: thomas.wirth@uni-ulm.de, tel: +49 73150023271

This PDF file includes:

Figures S1 to S7

Tables S1 to S3

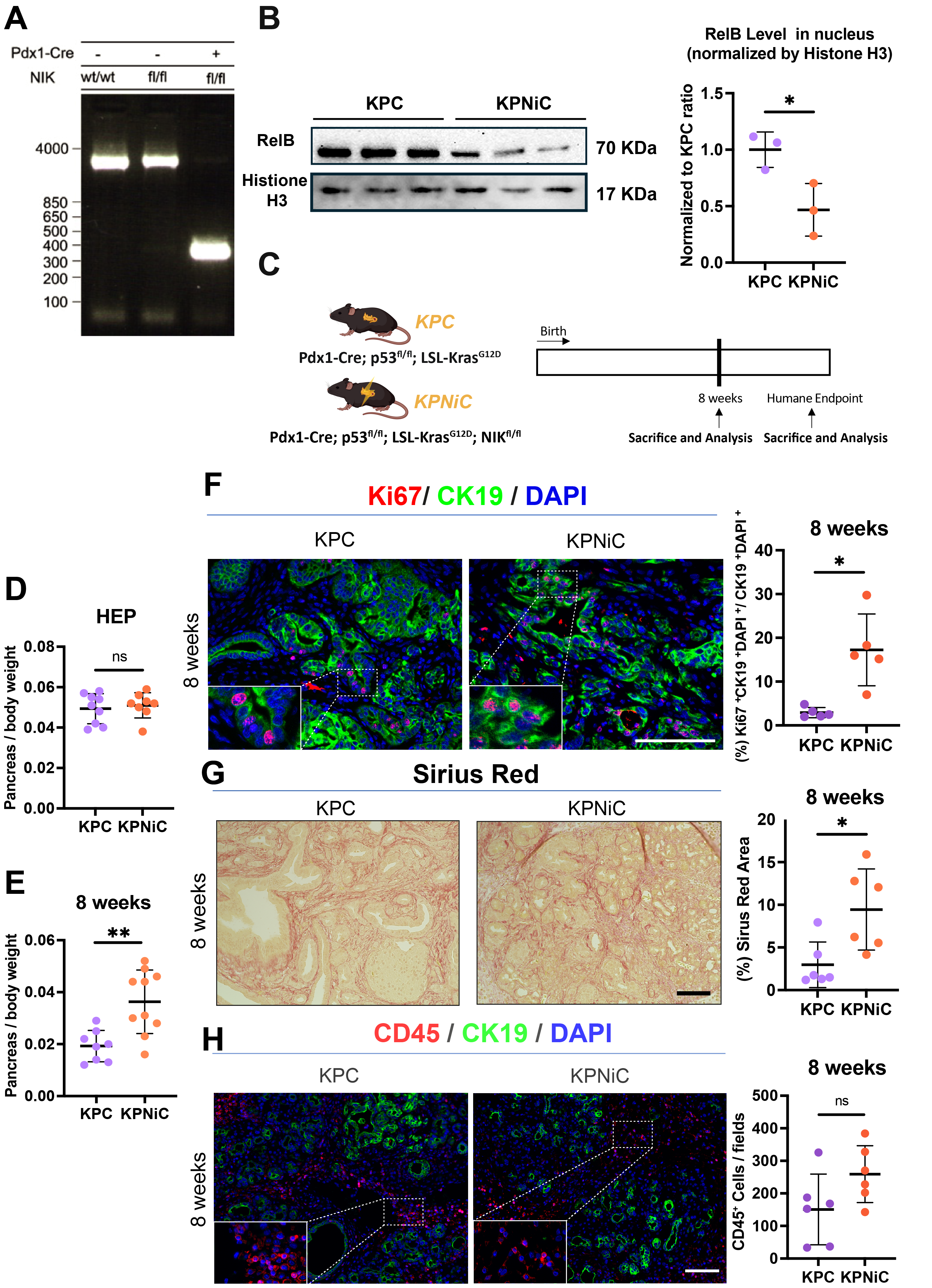

A
Pdx1-Cre - - +
NIK wt/wt fl/fl fl/fl
4000
850
650
500
400
300
200
100
B
KPC KPNiC
RelB
Histione H3
70 KDa
17 KDa
RelB Level in nucleus (normalized by Histone H3)
Normalized to KPC ratio
1.5
1.0
0.5
0.0
*
KPC KPNiC
C
KPC
Pdx1-Cre; p53fl/fl; LSL-KrasG12D
KPNiC
Pdx1-Cre; p53fl/fl; LSL-KrasG12D; NIKfl/fl
Birth
8 weeks
Humane Endpoint
Sacrifice and Analysis
Sacrifice and Analysis
D
HEP
Pancreas / body weight
0.08
0.06
0.04
0.02
0.00
ns
KPC KPNiC
E
8 weeks
Pancreas / body weight
0.06
0.04
0.02
0.00
**
KPC KPNiC
F
Ki67/ CK19 / DAPI
KPC
KPNiC
8 weeks
8 weeks
(%) Ki67 +CK19 +DAPI +/ CK19 +DAPI +
40
30
20
10
0
*
KPC KPNiC
G
Sirius Red
KPC
KPNiC
8 weeks
8 weeks
(%) Sirus Red Area
20
15
10
5
0
*
KPC KPNiC
H
CD45 / CK19 / DAPI
KPC
KPNiC
8 weeks
8 weeks
CD45+ Cells / fields
500
400
300
200
100
0
ns
KPC KPNiC

Supplementary Figure S1: NIK ablation effects in the KPC background. **(A)** PCR genotyping showing Cre-mediated recombination of the floxed NIK allele in Pdx1-Cre; $NIK^{fl/fl}$ mice. The 4 kb band corresponds to the unrecombined allele; the 400 bp band indicates successful recombination. **(B) Left:** Western blot of nuclear RelB levels in KPC and KPNiC cancer cells. **Right**: Quantification of nuclear RelB in KPC and KPNiC cancer cells. N≥3 biological replicates. Unpaired Student's t test. **(C)** Schematic representation of mouse genotypes used: KPC (Pdx1-Cre; $p53^{fl/fl}$; $KRAS^{G12D}$), and KPNiC (Pdx1-Cre; $p53^{fl/fl}$; $KRAS^{G12D}$; $NIK^{fl/fl}$). Mice were sacrificed at 8 weeks or monitored longitudinally and euthanized upon reaching humane endpoints (HEP) for analysis. **(D)** Quantification of pancreas / body weight in KPC and KPNiC mice. N≥8. t=HEP. Unpaired Student's t test. **(E)** Quantification of pancreas / body weight in KPC and KPNiC mice. N≥8. t=8 weeks. Unpaired Student's t test. **(F) Left:** Immunofluorescence staining of Ki67 (red), CK19 (green), and DAPI (blue) in pancreata from KPC and KPNiC mice. Scale bar: 100 μm. **Right:** Quantification of $Ki67^+/CK19^+/DAPI^+$ cells as a percentage of total $CK19^+/DAPI^+$ cells. N=5 mice/group. t=8 weeks. Welch's t test. **(G) Left:** Representative Sirius Red staining showing collagen deposition in pancreata sections in KPC and KPNiC mice. Scale bar: 100 μm. **Right:** Quantification of Sirius Red^+ fibrotic area (% of total tissue area). N=6 mice/group. t=8 weeks. Unpaired Student's t test. **(H) Left:** Representative immunofluorescence staining of tumor sections in KPC and KPNiC mice at 8 weeks for CK19 (green), CD45 (red), and DAPI (blue). Scale bar: 100 μm. **Right:** Quantification of $CD45^+$ immune cells per field in tumors from KPC and KPNiC mice. N=6 mice/group. t=8 weeks. Unpaired Student's t test.

Dot plots represent individual mice. Data presented as mean ± SD. ns: $p > 0.05$, *$p < 0.05$, **$p < 0.01$

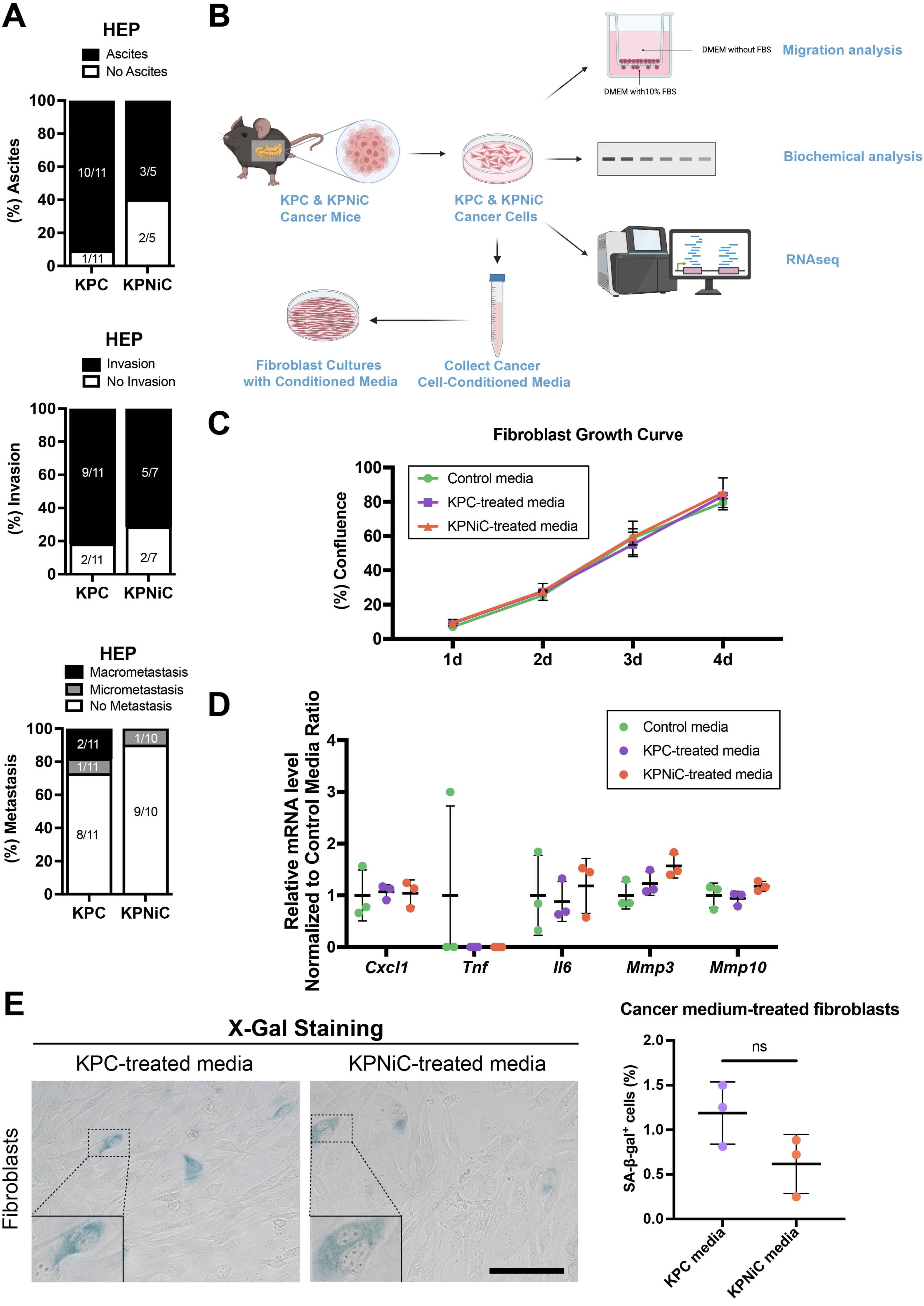
A
HEP
Ascites
No Ascites
(%) Ascites
10/11
3/5
1/11
2/5
KPC
KPNiC
HEP
Invasion
No Invasion
(%) Invasion
9/11
5/7
2/11
2/7
KPC
KPNiC
HEP
Macrometastasis
Micrometastasis
No Metastasis
(%) Metastasis
2/11
1/11
8/11
1/10
9/10
KPC
KPNiC
B
DMEM without FBS
Migration analysis
DMEM with10% FBS
Biochemical analysis
KPC & KPNiC Cancer Mice
KPC & KPNiC Cancer Cells
RNAseq
Fibroblast Cultures with Conditioned Media
Collect Cancer Cell-Conditioned Media
C
Fibroblast Growth Curve
(%) Confluence
Control media
KPC-treated media
KPNiC-treated media
1d
2d
3d
4d
D
Relative mRNA level Normalized to Control Media Ratio
Control media
KPC-treated media
KPNiC-treated media
Cxcl1
Tnf
Il6
Mmp3
Mmp10
E
X-Gal Staining
KPC-treated media
KPNiC-treated media
Fibroblasts
Cancer medium-treated fibroblasts
SA-β-gal+ cells (%)
ns
KPC media
KPNiC media

Supplementary Figure S2: Malignant features of KPC/KPNiC mice, cell experiment scheme and fibroblasts senescence. **(A)** Assessment of malignant features at the humane endpoint in KPC and KPNiC mice. **Top**: Quantification of ascites in KPC and KPNiC mice. **Middle**: Quantification of invasive tumor growth into surrounding tissues. **Bottom**: Frequency of distant metastasis, categorized as macrometastasis, micrometastasis, or no metastasis. Data are presented as percentage of animals in each category, with numbers shown as n/total. Chi-square test. No significant difference in ascites, invasion, or distant metastasis was observed between groups. **(B)** Schematic illustration of experimental design: cancer cells were isolated from KPC or KPNiC tumors and cultured to collect cancer cell–conditioned medium (CM). CM was applied to primary fibroblasts for later experiments. Cancer cells were also used for biochemical analysis, migration assay, and RNA sequencing. **(C)** Growth curve of fibroblasts cultured in DMEM, KPC-conditioned medium, or KPNiC-conditioned medium over 4 days. No significant difference in proliferation was observed between groups. N≥3 biological replicates. One-way ANOVA test. **(D)** mRNA level of senescence-associated markers from fibroblasts treated with DMEM, KPC or KPNiC culture media. N≥3 biological replicates. One-way ANOVA with Bonferroni post hoc for *Cxcl1, Tnf, Il6, mmp10*. Welch ANOVA test for Mmp3. **(E)** **Left**: X-Gal staining on fibroblasts treated with KPC or KPNiC culture media. **Right**: Quantification of the SA-β-gal$^+$ cells. N≥3 biological replicates. Unpaired student's t test. (Scale bar: 100 μm).

Dot plots represent individual mice. Data presented as mean ± SD. ns: $p > 0.05$

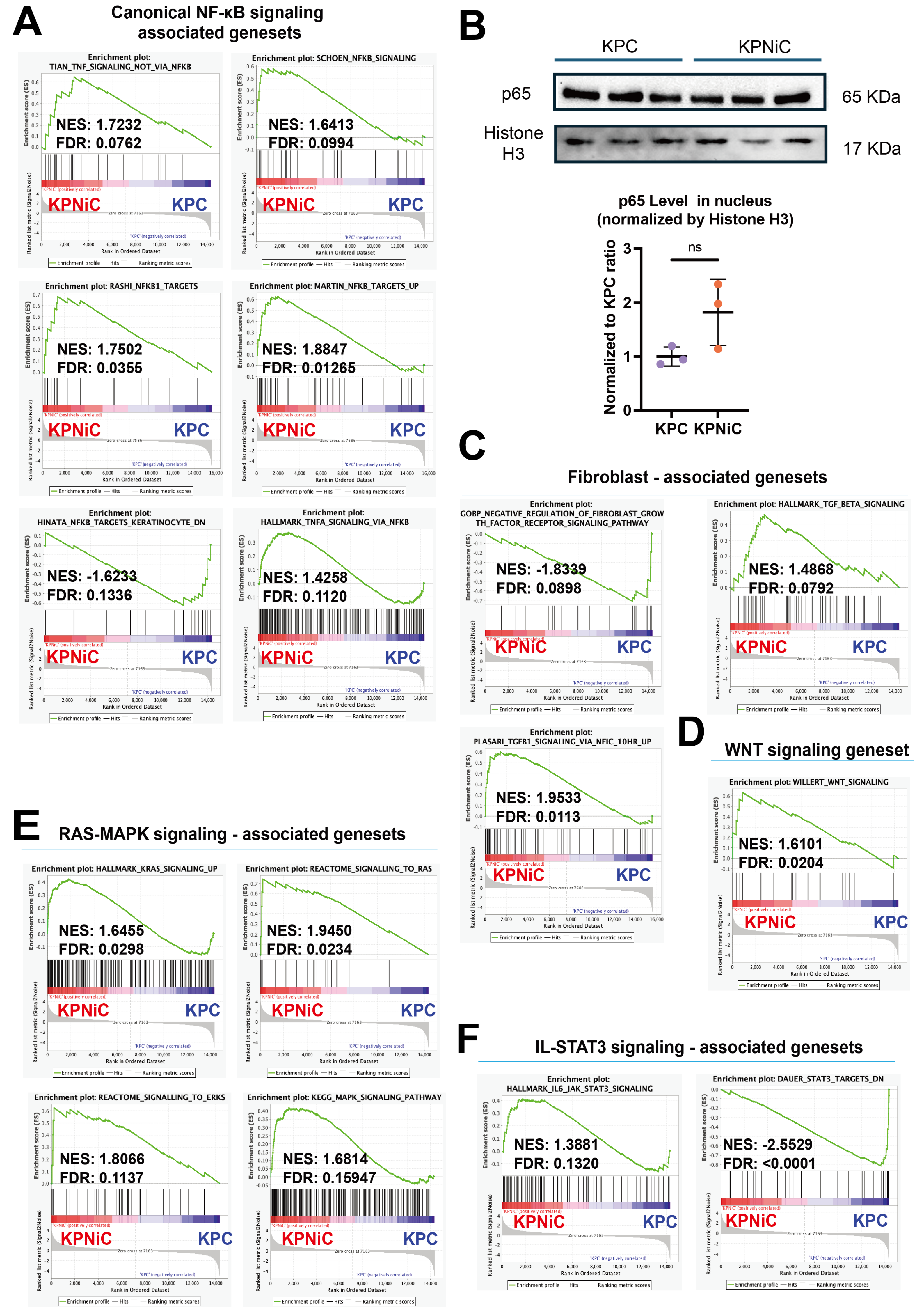
A
Canonical NF-κB signaling associated genesets
Enrichment plot: TIAN_TNF_SIGNALING_NOT_VIA_NFKB
NES: 1.7232
FDR: 0.0762
Enrichment plot: SCHOEN_NFKB_SIGNALING
NES: 1.6413
FDR: 0.0994
Enrichment plot: RASHI_NFKB1_TARGETS
NES: 1.7502
FDR: 0.0355
Enrichment plot: MARTIN_NFKB_TARGETS_UP
NES: 1.8847
FDR: 0.01265
Enrichment plot: HINATA_NFKB_TARGETS_KERATINOCYTE_DN
NES: -1.6233
FDR: 0.1336
Enrichment plot: HALLMARK_TNFA_SIGNALING_VIA_NFKB
NES: 1.4258
FDR: 0.1120
KPNiC
KPC
Enrichment score (ES)
Ranked list metric (Signal2Noise)
Rank in Ordered Dataset
Enrichment profile — Hits Ranking metric scores
B
KPC
KPNiC
p65
65 KDa
Histone H3
17 KDa
p65 Level in nucleus (normalized by Histone H3)
Normalized to KPC ratio
ns
KPC KPNiC
C
Fibroblast - associated genesets
Enrichment plot: GOBP_NEGATIVE_REGULATION_OF_FIBROBLAST_GROWTH_FACTOR_RECEPTOR_SIGNALING_PATHWAY
NES: -1.8339
FDR: 0.0898
Enrichment plot: HALLMARK_TGF_BETA_SIGNALING
NES: 1.4868
FDR: 0.0792
Enrichment plot: PLASARI_TGFB1_SIGNALING_VIA_NFIC_10HR_UP
NES: 1.9533
FDR: 0.0113
D
WNT signaling geneset
Enrichment plot: WILLERT_WNT_SIGNALING
NES: 1.6101
FDR: 0.0204
E
RAS-MAPK signaling - associated genesets
Enrichment plot: HALLMARK_KRAS_SIGNALING_UP
NES: 1.6455
FDR: 0.0298
Enrichment plot: REACTOME_SIGNALLING_TO_RAS
NES: 1.9450
FDR: 0.0234
Enrichment plot: REACTOME_SIGNALLING_TO_ERKS
NES: 1.8066
FDR: 0.1137
Enrichment plot: KEGG_MAPK_SIGNALING_PATHWAY
NES: 1.6814
FDR: 0.15947
F
IL-STAT3 signaling - associated genesets
Enrichment plot: HALLMARK_IL6_JAK_STAT3_SIGNALING
NES: 1.3881
FDR: 0.1320
Enrichment plot: DAUER_STAT3_TARGETS_DN
NES: -2.5529
FDR: <0.0001

Supplementary Figure S3. Gene Set Enrichment Analysis (GSEA) plots comparing transcriptomic signatures between KPNiC and KPC cancer cells. **(A)** Canonical NF-κB signaling–associated gene sets: TIAN TNF SIGNALING NOT VIA NFKB; SCHOEN NFKB SIGNALING; HALLMARK TNFA SIGNALING VIA NFKB; RASHI NFKB1 TARGETS; MARTIN NFKB TARGETS UP; HINATA NFKB TARGETS KERATINOCYTE DN. **(B) Top**: Western blot of nuclear RelA levels in KPC and KPNiC cancer cells. **Bottom**: Quantification of nuclear RelA in KPC and KPNiC cancer cells. N=3 biological replicates. Unpaired student's t test. Data presented as mean ± SD. ns: $p > 0.05$. **(C)** Fibroblast-associated gene sets: GOBP NEGATIVE REGULATION OF FIBROBLAST GROWTH FACTOR RECEPTOR SIGNALING PATHWAY; PLASARI TGFB1 SIGNALING VIA VCAM1 10HR UP; HALLMARK TGF BETA SIGNALING. **(D)** WNT signaling gene set: WILLERT WNT SIGNALING. **(E)** RAS-MAPK signaling–associated gene sets: HALLMARK KRAS SIGNALING UP; REACTOME SIGNALING TO RAS; REACTOME SIGNALING TO ERKS; KEGG MAPK SIGNALING PATHWAY; **(F)** IL-STAT3 signaling–associated gene sets: HALLMARK IL6 JAK STAT3 SIGNALING; DAUER STAT3 TARGETS DN.

NES: Normalized Enrichment Score; FDR: False Discovery Rate. Gene sets enriched in KPNiC cells are indicated in red, and gene sets enriched in KPC cells are indicated in blue.

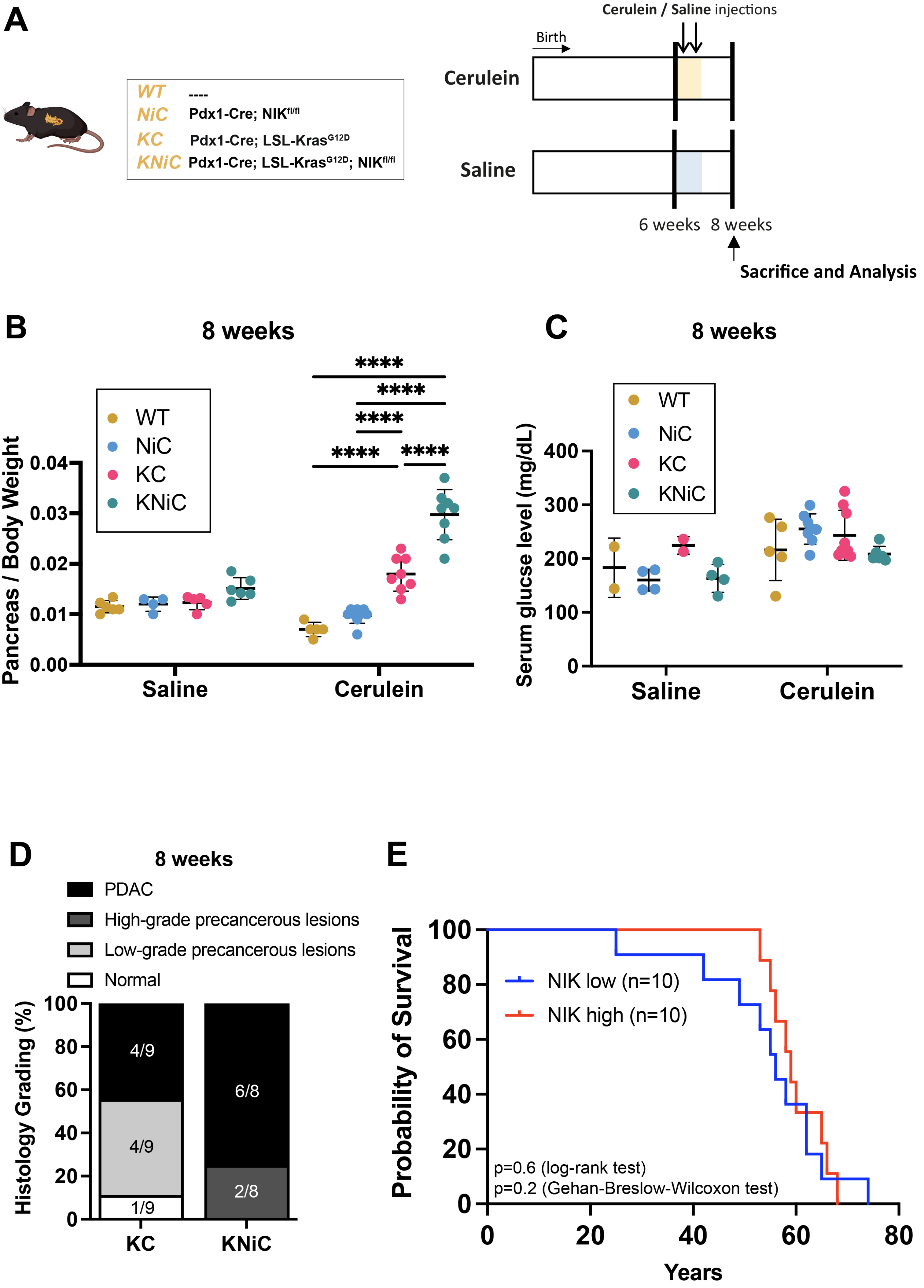

A
WT ----
NiC Pdx1-Cre; NIKfl/fl
KC Pdx1-Cre; LSL-KrasG12D
KNiC Pdx1-Cre; LSL-KrasG12D; NIKfl/fl
Cerulein / Saline injections
Birth
Cerulein
Saline
6 weeks
8 weeks
Sacrifice and Analysis
B
8 weeks
WT
NiC
KC
KNiC
****
Pancreas / Body Weight
0.04
0.03
0.02
0.01
0.00
Saline
Cerulein
C
8 weeks
Serum glucse level (mg/dL)
400
300
200
100
0
D
8 weeks
PDAC
High-grade precancerous lesions
Low-grade precancerous lesions
Normal
Histology Grading (%)
4/9
4/9
1/9
6/8
2/8
KC
KNiC
E
Probability of Survival
NIK low (n=10)
NIK high (n=10)
p=0.6 (log-rank test)
p=0.2 (Gehan-Breslow-Wilcoxon test)
Years
0
20
40
60
80
100

Supplementary Figure S4: Examination of 8-week KC and KNiC mice. **(A)** Schematic of experimental setup: WT, Pdx1-Cre; $NIK^{fl/fl}$ (NiC), Pdx1-Cre; LSL-$Kras^{G12D}$ (KC) and Pdx1-Cre; $NIK^{fl/fl}$; LSL-$Kras^{G12D}$ (KNiC) mice were injected with cerulein or saline at 6 weeks of age and analyzed 2 weeks later. **(B)** Quantification of pancreas-to-body weight ratio in WT, NiC, KC, and KNiC mice treated with saline or cerulein. N≥4 mice/group. t=8 weeks. Two-way ANOVA with Tukey's post hoc. **(C)** Blood glucose levels (mg/dL) in WT, NiC, KC, and KNiC mice under saline or cerulein treatment. N≥4 mice/group. t=8 weeks. $p > 0.05$, two-way ANOVA with Tukey's post hoc. **(D)** Histological grading of pancreata from cerulein-treated KC and KNiC mice, classified as normal, low-grade precancerous lesions, high-grade precancerous lesions, or PDAC. N≥8 mice/group. t=8 weeks. **(E**) Kaplan-Meier survival analysis of PDAC patients with high (red line, n = 10) vs. low (blue line, n=10) expression of NIK. Log-rank test and Gehan-Breslow-Wilcoxon test.

Dot plots represent individual mice. Data presented as mean ± SD. ****$p < 0.0001$

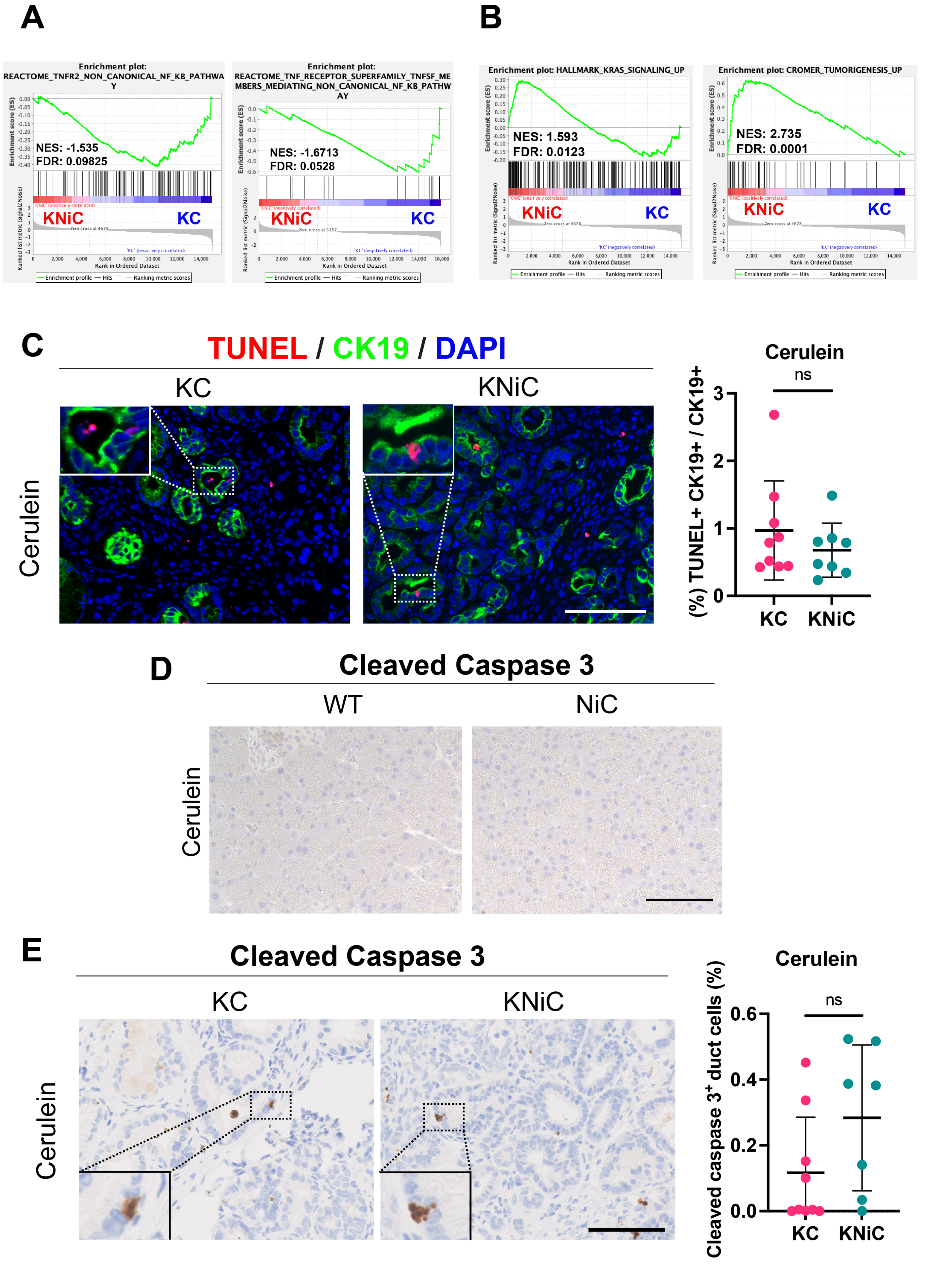
A
B
Enrichment plot: REACTOME_TNFR2_NON_CANONICAL_NF_KB_PATHWAY
NES: -1.535
FDR: 0.09825
Enrichment plot: REACTOME_TNF_RECEPTOR_SUPERFAMILY_TNFSF_MEMBERS_MEDIATING_NON_CANONICAL_NF_KB_PATHWAY
NES: -1.6713
FDR: 0.0528
Enrichment plot: HALLMARK_KRAS_SIGNALING_UP
NES: 1.593
FDR: 0.0123
Enrichment plot: CROMER_TUMORIGENESIS_UP
NES: 2.735
FDR: 0.0001
KNiC
KC
Enrichment score (ES)
Ranked list metric (Signal2Noise)
Rank in Ordered Dataset
Enrichment profile — Hits — Ranking metric scores
C
TUNEL / CK19 / DAPI
KC
KNiC
Cerulein
(%) TUNEL+ CK19+ / CK19+
ns
D
Cleaved Caspase 3
WT
NiC
Cerulein
E
Cleaved Caspase 3
KC
KNiC
Cerulein
Cleaved caspase 3+ duct cells (%)
ns

Supplementary Figure S5: The effect of NIK deletion in the survival and cell death of pancreatic cells. **(A)** GSEA enrichment plots for the gene sets REACTOME TNFR2 NON CANONICAL NF KB PATHWAY and REACTOME TNF RECEPTOR SUPERFAMILY TNFSF MEMBERS MEDIATING NON CANONICAL NF KB PATHWAY comparing KNiC with KC pancreata. Gene sets enriched in KNiC are shown in red; those enriched in KC are shown in blue. NES = normalized enrichment score; FDR = false discovery rate. **(B)** GSEA enrichment plots for the gene sets HALLMARK KRAS SIGNALING UP and CROMER TUMORIGENESIS UP comparing KNiC with KC pancreata. **(C) Left:** Representative images of TUNEL (red) and CK19 (green) co-staining in KC and KNiC mice under cerulein treatment. **Right:** Quantification of the proportion (%) of TUNEL$^+$ CK19$^+$ cells per among CK19$^+$ cells. N≥7 mice/group. t=8 weeks. Unpaired Student's t test. **(D)** Representative images of cleaved caspase 3 in WT and NiC mice under cerulein treatment. **(E) Left**: Representative images of cleaved caspase 3 in KC and KNiC mice under cerulein treatment. **Right:** Quantification of the proportion (%) of cleaved caspase 3$^+$ apoptotic cells among duct cells. N≥7 mice/group. t=8 weeks. Unpaired Student's t test.

Dot plots represent individual mice. Data presented as mean ± SD. ns: $p > 0.05$. Scale bar: 100 µm.

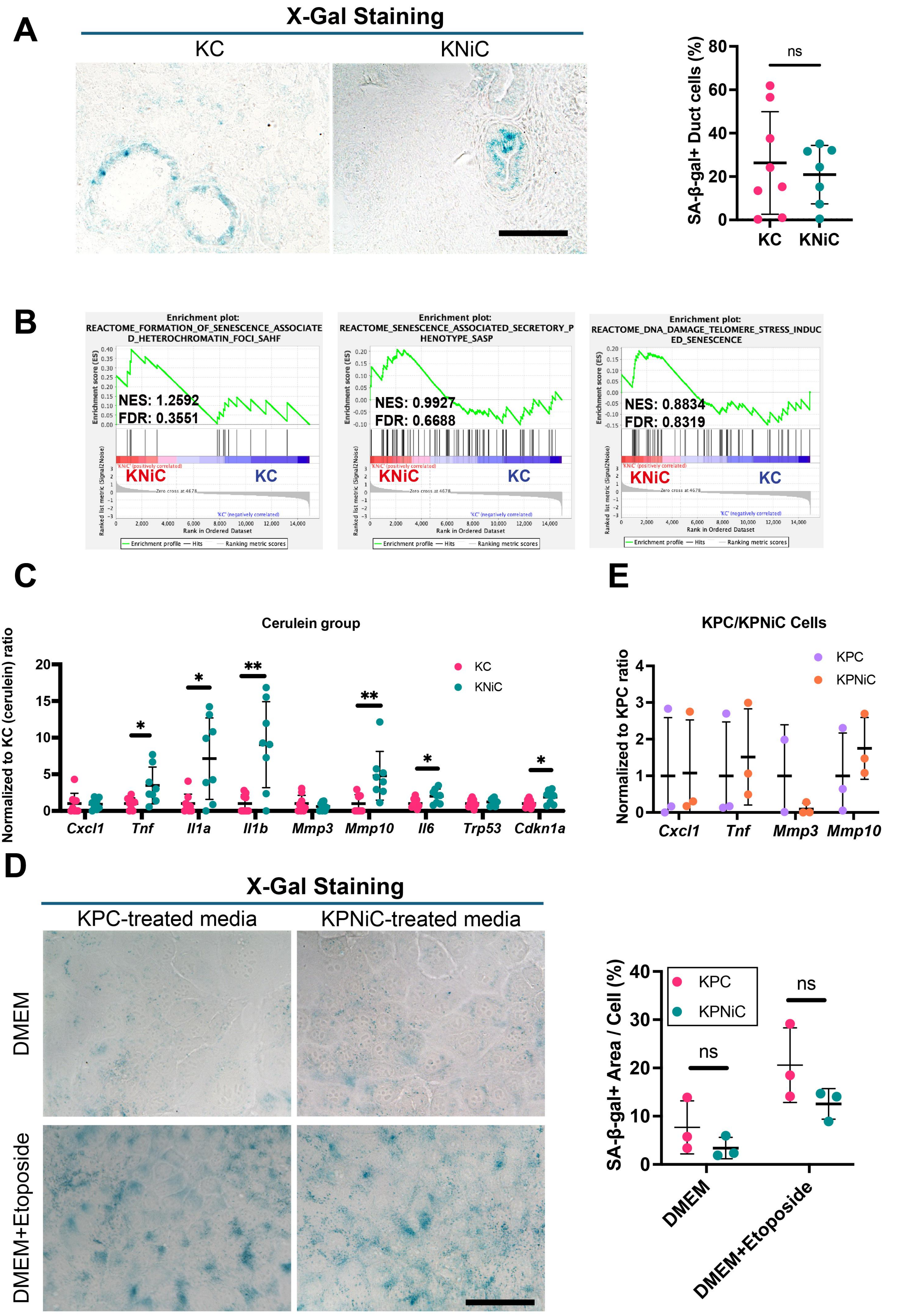
A
X-Gal Staining
KC
KNiC
SA-β-gal+ Duct cells (%)
ns
KC
KNiC
B
Enrichment plot: REACTOME_FORMATION_OF_SENESCENCE_ASSOCIATED_HETEROCHROMATIN_FOCI_SAHF
NES: 1.2592
FDR: 0.3551
Enrichment plot: REACTOME_SENESCENCE_ASSOCIATED_SECRETORY_PHENOTYPE_SASP
NES: 0.9927
FDR: 0.6688
Enrichment plot: REACTOME_DNA_DAMAGE_TELOMERE_STRESS_INDUCED_SENESCENCE
NES: 0.8834
FDR: 0.8319
KNiC
KC
Enrichment score (ES)
Ranked list metric (Signal2Noise)
Rank in Ordered Dataset
Enrichment profile — Hits — Ranking metric scores
C
Cerulein group
Normalized to KC (cerulein) ratio
KC
KNiC
Cxcl1
Tnf
Il1a
Il1b
Mmp3
Mmp10
Il6
Trp53
Cdkn1a
D
X-Gal Staining
KPC-treated media
KPNiC-treated media
DMEM
DMEM+Etoposide
SA-β-gal+ Area / Cell (%)
KPC
KPNiC
ns
ns
DMEM
DMEM+Etoposide
E
KPC/KPNiC Cells
Normalized to KPC ratio
KPC
KPNiC
Cxcl1
Tnf
Mmp3
Mmp10

Supplementary Figure S6: The effect of NIK deletion in senescence of pancreatic tumor cells. **(A) Left**: X-Gal staining on pancreata from 8-week-old cerulein-injected KC and KNiC mice. Scale bar: 100 μm. **Right**: Quantification of X-Gal+ duct cells. N≥7 mice/group. t=8 weeks. Unpaired Student's t test. **(B)** GSEA enrichment plots for the gene sets REACTOME FORMATION OF SENESCENCE ASSOCIATED HETEROCHROMATIN FOCI SAHF, REACTOME SENESCENCE ASSOCIATED SECRETORY PHENOTYPE SASP and REACTOME DNA DAMAGE TELOMERE STRESS INDUCED SENESCENCE comparing KNiC with KC pancreata. Gene sets enriched in KNiC are shown in red; those enriched in KC are shown in blue. NES = normalized enrichment score; FDR = false discovery rate. **(C)** mRNA levels of senescence-associated markers on pancreata from 8-week-old cerulein-injected KC and KNiC mice. N≥7 mice/group. t=8 weeks. Unpaired Student's t test. **(D) Left:** X-Gal staining on untreated and etoposide-treated KPC and KPNiC cancer cells. Scale bar: 100 μm. **Right:** Quantification of SA-β-gal+ area. N=3 biological replicates/group. Unpaired Student's t test. **(E)** mRNA levels of senescence-associated markers on KPC and KPNiC cancer cells. N=3 biological replicates /group. Unpaired Student's t test.

Dot plots represent individual mice. Data presented as mean ± SD. ns: $p > 0.05$, *$p < 0.05$, **$p<0.01$

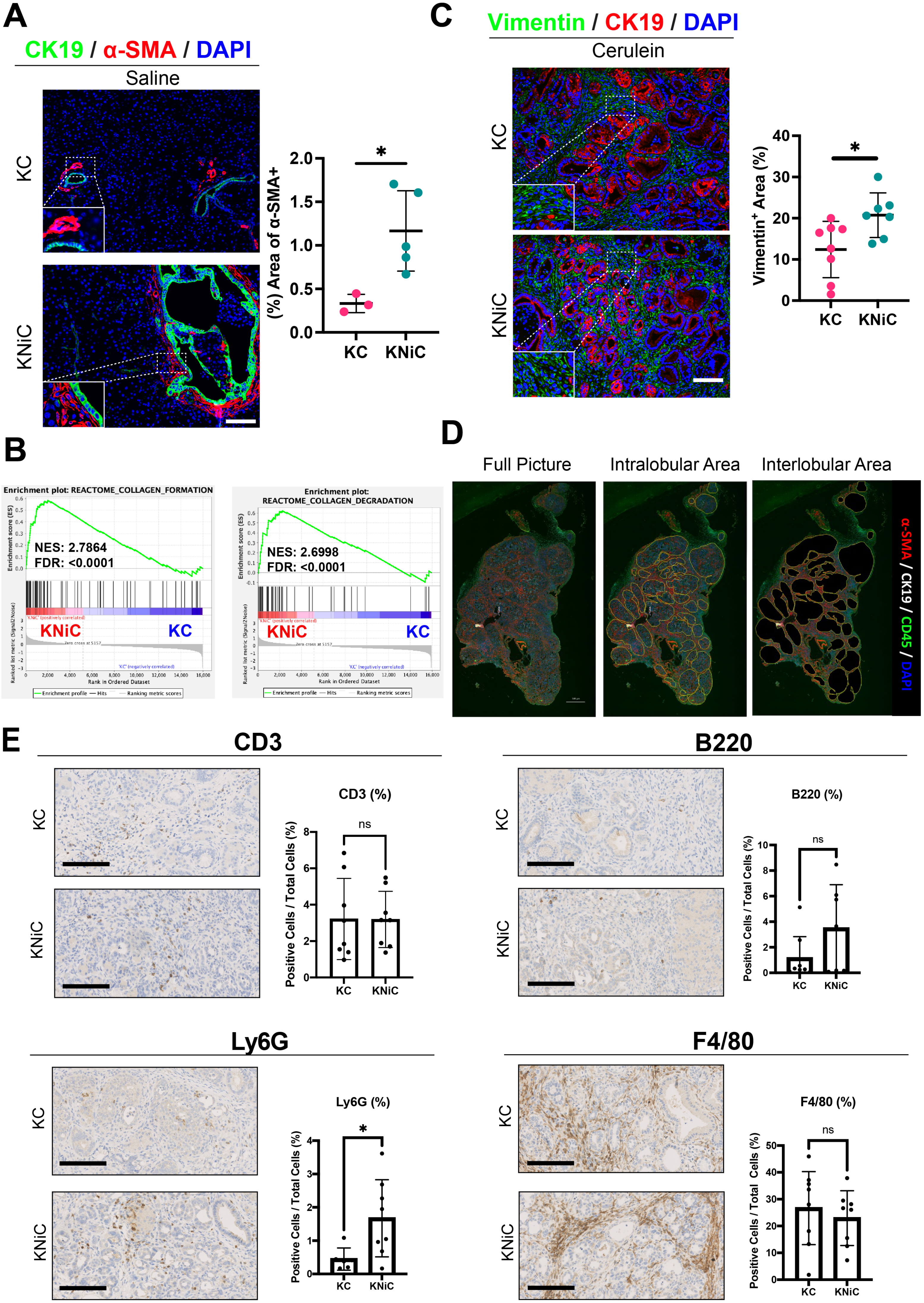
A
CK19 / α-SMA / DAPI
Saline
KC
KNiC
(%) Area of α-SMA+
2.0
1.5
1.0
0.5
0.0
KC
KNiC
*
B
Enrichment plot: REACTOME_COLLAGEN_FORMATION
NES: 2.7864
FDR: <0.0001
Enrichment plot: REACTOME_COLLAGEN_DEGRADATION
NES: 2.6998
FDR: <0.0001
KNiC
KC
Rank in Ordered Dataset
Enrichment profile
Hits
Ranking metric scores
C
Vimentin / CK19 / DAPI
Cerulein
KC
KNiC
Vimentin+ Area (%)
40
30
20
10
0
*
D
Full Picture
Intralobular Area
Interlobular Area
α-SMA / CK19 / CD45 / DAPI
E
CD3
CD3 (%)
ns
Positive Cells / Total Cells (%)
KC
KNiC
B220
B220 (%)
ns
Ly6G
Ly6G (%)
*
F4/80
F4/80 (%)
ns

Supplementary Figure S7: (A) Left: Representative immunofluorescence staining of pancreatic sections from saline-treated KC and KNiC mice showing α-SMA (red), ductal marker CK19 (green), and DAPI (blue). t=8 weeks. Scale bar: 100 µm. Right**:** Quantification of α-SMA positive area (%). N≥3 mice/group. t=8 weeks. Unpaired Student's t test. **(B)** GSEA enrichment plots for the REACTOME COLLAGEN FORMATION and REACTOME COLLAGEN DEGRADATION gene sets comparing KNiC and KC pancreata at 8 weeks. Gene sets enriched in KNiC are shown in red; those enriched in KC are shown in blue. **(C) Left:** Immunofluorescence staining for Vimentin and CK19 on pancreata from 8-week-old cerulein-injected KC and KNiC mice. Scale bar: 100 µm. **Right:** Quantification of the Vimentin^{+} area (%). N≥7 mice/group. t=8 weeks. Unpaired Student's t test. **(D)** Whole-section immunofluorescence template used for region-specific stromal quantification. **Left:** composite image of a pancreas stained for α-SMA (red), CK19 (white), CD45 (green), and DAPI (blue). **Middle:** intralobular region delineated by yellow contours drawn inside CK19-positive lobules. **Right:** mask for interlobular assessment—black regions are the intralobular lobules that have been removed; the remaining coloured tissue constitutes the interlobular stroma to be quantified. Scale bar: 500 µm. **(E)** Representative immunohistochemistry and quantification of immune cell markers in cerulein-treated KC and KNiC pancreata: CD3^{+} T cells, B220^{+} B cells, Ly6G^{+} neutrophils, F4/80^{+} macrophages. Scale bars: 100 µm. Right panels: quantification of positive cells / Total cells (%). N≥7 mice/group. t=8 weeks. Unpaired Student's t test.

Dot plots represent individual mice. Data presented as mean ± SD. ns: $p > 0.05$, *$p < 0.05$,

Supplementary Table S1. Number of captured fields per staining for statistical analysis.

Type of staining	Number of fields
H&E Cancer Grading/Precancerous grading	Whole section
H&E staining for total remodeling area	Whole section
H&E staining for low grade lesions	≥6 random fields
CD45/CK19/DAPI staining	Whole section/≥10 random fields
F4/80 staining	≥8 random fields
CD3 staining	≥8 random fields
Ly6G staining	≥8 random fields
B220 staining	≥8 random fields
α-SMA/CK19/DAPI staining	6 random fields
Azan Trichrome staining	6 random fields
Sirius Red staining	6 random fields
α-amylase/Ki67/DAPI staining	6 random fields
CK19/Ki67/DAPI staining	6 random fields
TUNEL/CPA2 or CK19/DAPI staining	≥6 random fields
DAPI/*Map3k14*	≥5 random fields
CK19/Amy/DAPI staining	≥6 random fields
X-Gal staining	6 random fields
Cleaved Caspase 3 staining	6 random fields
Vimentin/CK19/DAPI staining	6 random fields

Supplementary Table S2: List of immunofluorescence (IF), immunohistochemistry (IHC) and Western Blot (WB) antibodies

Antibody (Dilution)	Catalog Number	Application
Ki67 (1:200)	Cell Signaling, #9449S	IF
α-SMA (1:200)	Millipore, #CBL 171	IF
Amylase (1:200)	Sigma, #A8273	IF
CK7 (Ready to use)	DAKO, #GA619	IF
CK19 (1:200)	Millipore, #MABT913	IF
CD45 (1:100)	BD Biosciences, #550539	IF
CPA2 (1:200)	ThermoFisher, #MA5-29691	IF
Vimentin (1:100)	Abcam, #ab92547	IF
NIK (1:100)	Novus biologicals, NBP1-83413	IF
Donkey anti-mouse 488 (1:500)	Invitrogen, #A-21202	IF
Donkey anti-mouse 555 (1:500)	Invitrogen, #A-32773	IF
Donkey anti-mouse 594 (1:500)	Invitrogen, #A-21203	IF
Donkey anti-mouse 647 (1:500)	Invitrogen, #A-31573	IF
Donkey anti-rabbit 488 (1:500)	Invitrogen, #A-21206	IF
Donkey anti-rabbit 594 (1:500)	Invitrogen, #A-21207	IF
Donkey anti-rabbit 647 (1:500)	Invitrogen, #A-32795	IF
Donkey anti-rat 488 (1:500)	Invitrogen, #A-21208	IF
Donkey anti-rat 555 (1:500)	Invitrogen, #A-48270	IF
Donkey anti-rat 647 (1:500)	Invitrogen, #A-48272	IF
Fc block (1:100)	BD Biosciences, #553142	IF
Fab block (1:40)	Jackson Immunoresearch, #715-007-003	IF
Cleaved Caspase 3 (1:100)	Cell Signaling, #9664S	IHC
B220 (1:4000)	Pharmigen, #553084	IHC
CD3 (1:300)	ThermoFischer, #MA1-90582	IHC
Ly6G (1:600)	Pharmigen, #551459	IHC
F4/80 (1:50)	Biomedicals AG, #T-2006	IHC
pSTAT3 (1:1000)	Cell Signaling, #9145	WB
STAT3 (1:1000)	Cell Signaling, #4904	WB
pERK 1/2 (1:1000)	Cell Signaling, #4370	WB
ERK 2 (1:1000)	Santa Cruz, #154	WB
Zeb1 (1:1000)	Santa Cruz, #25388	WB
E-Cadherin (1:1000)	Cell signaling, #14472	WB

RelB	Cell signaling, #10544	WB / IF
p65	Santa Cruz, #sc-372	WB
Histone H3 (1:5000)	Abcam, #ab1791	WB
GAPDH (1:1000)	Santa Cruz, #25778	WB
Anti-rabbit HRP (1:5000)	Santa Cruz, #2004	WB

Supplementary Table S3: List of primers for qPCR

Gene	Forward primer sequence	Reverse primer sequence
Amy2a1	gga gaa att gat aac tac aat gat gc	acc ctg cta ctc caa tgt caa
Krt19	agt ccc agc tca gca tga a	taa cgg gcc tcc gtc tct
Fn1	gat gcc gat cag aag ttt gg	ggt tgt gca gat ctc ctc gt
Col1a1	cat gtt cag ctt tgt gga cct	gca gct gac ttc agg gat gt
Col3a1	tcc cct gga atc tgt gaa tc	tga gtc gaa ttg ggg aga at
Acta2	cac cac tga acc cta agg cc	cac ata cat ggc ggg gac at
Il6	gct acc aaa ctg gat ata atc agg a	cca ggt agc tat ggt act cca gaa
Il11	tct ttg cag ctt cct ggt gt	gga gta gcc gtt cca gtc g
Cxcl1	cag agc ctc taa cca gtt cca	tgg gat cat ggt gct gtg
Cxcl2	agg caa ggc taa ctg acc tg	ttc tct ttg gtt ctt ccg ttg
Emr1	gga gga ctt ctc caa gcc tat t	agg cct ctc aga ctt ctg ctt
Tnf	tgc cta tgt ctc agc ctc ttc	gag gcc attt ggg aac ttc t
Ccl2	cat cca cgt gtt ggc tca	gat cat ctt gct ggt gaa tga gt
Ccl8	ttc ttt gcc tgc tgc tca ta	gca ggt gac tgg agc ctt at
Il1a	ttg gtt aaa tga cct gca aca	gag cgc tca cga aca gtt g
Il1b	agt tga cgg acc cca aaa g	agc tgg atg ctc tca tca gg
Mmp3	tgc agc tct act ttg ttc tttg a	aga gat ttg cgc caa aag tg
Mmp10	tgg att ctg cca ttg aga aag	ggt aaa agt ctc cgt gtt ctc c
Cdkn1a	aac atc tca ggg ccg aaa	tgc gct tgg agt gat aga aa
Trp53	cag tct ggg aca gcc aag tc	cag ctg gca gaa tag ctt att ga
Rpl13	cct gct gct ctc aag gtt gt	ggt act tcc acc cga cct c
