# Supplementary material for: NF-κB inducing kinase (NIK) deletion accelerates KRAS-driven pancreatic cancer in association with tumor microenvironment remodeling
Source: Cell Death Dis. 2026 May 27;17(1):513. doi: 10.1038/s41419-026-08877-w (PMC13216544; doi:10.1038/s41419-026-08877-w)

Figure S1A

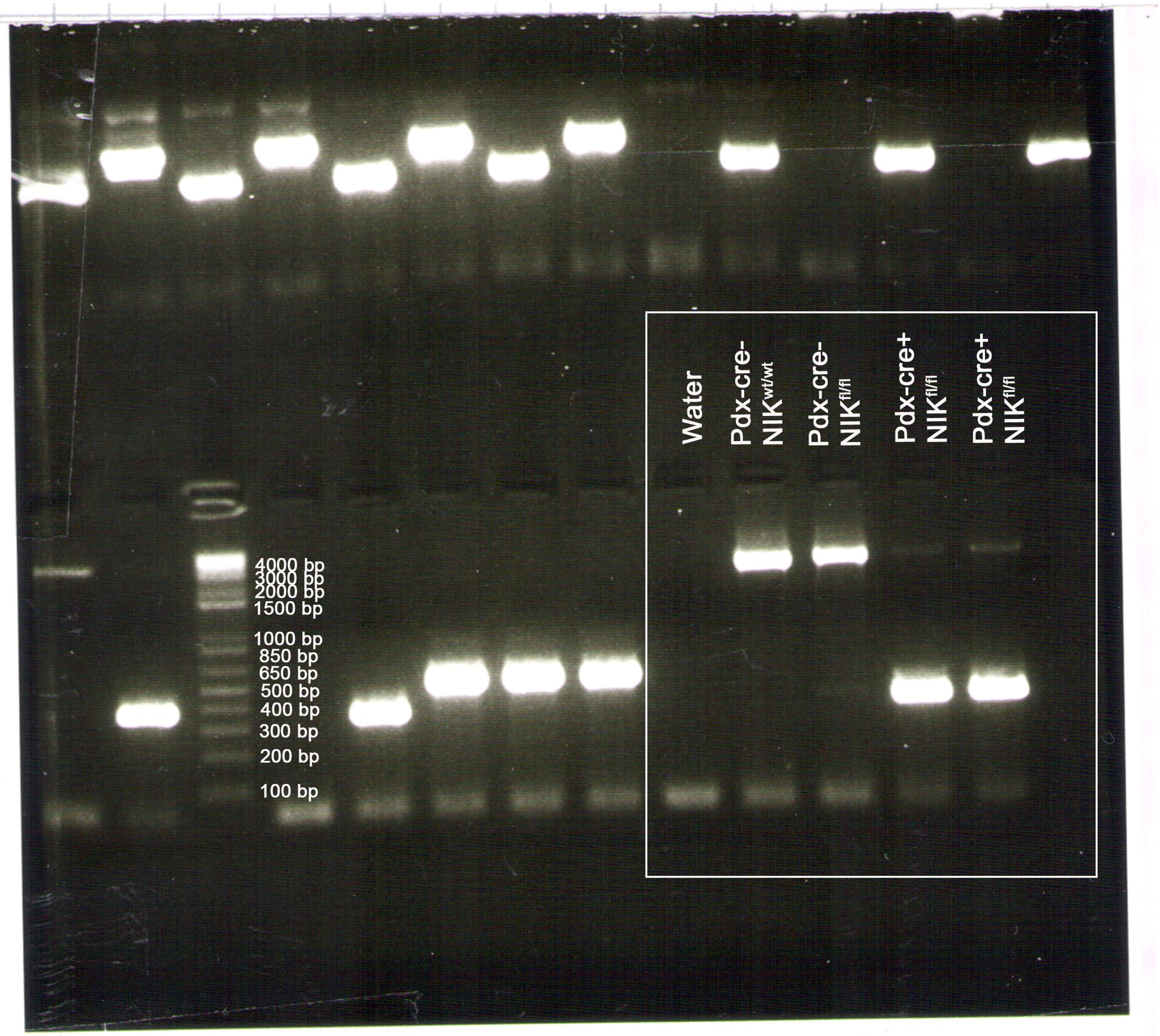

DNA ladder:1 kb Plus DNA-ladder(Invitrogen, #10787018)

Figure 2E

ZEB1

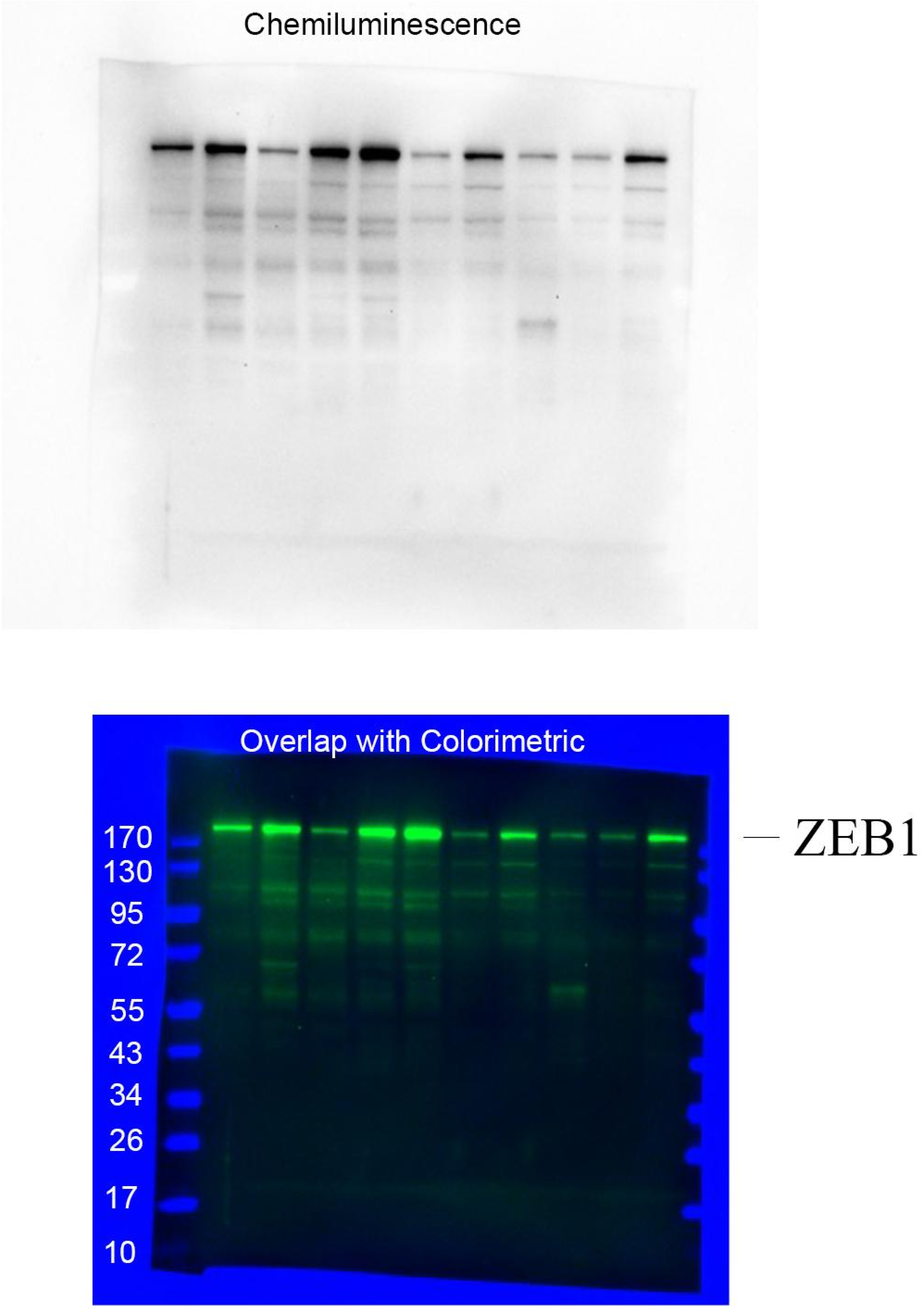

GAPDH

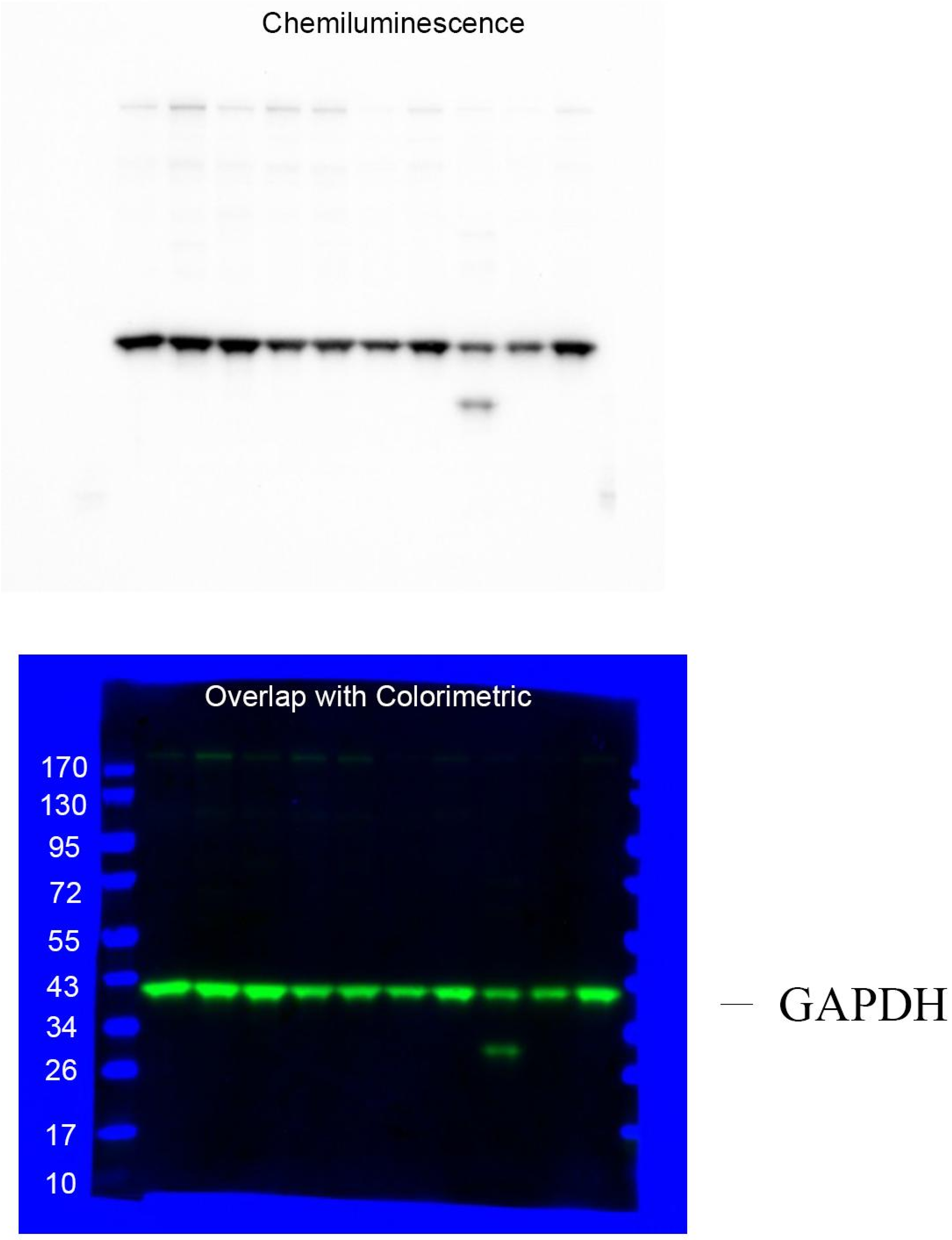

E-Cadherin

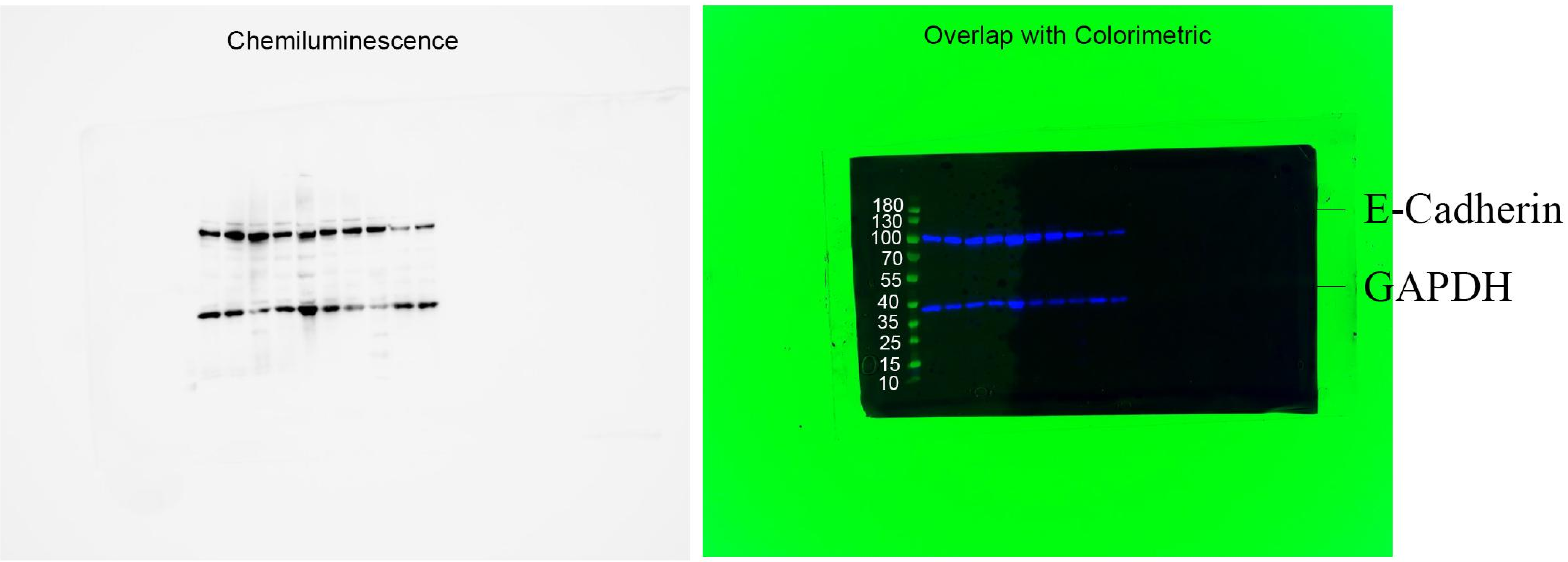

GAPDH for E-cadherin Quantification

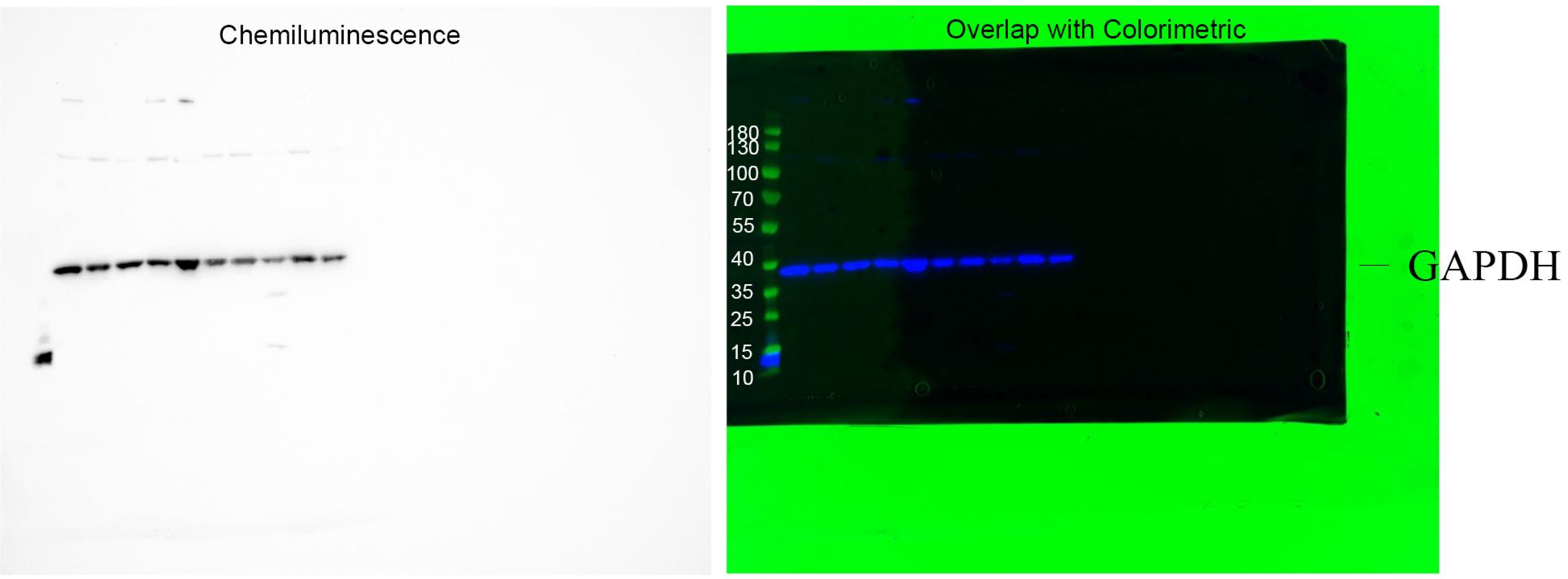

Figure 4C

pERK

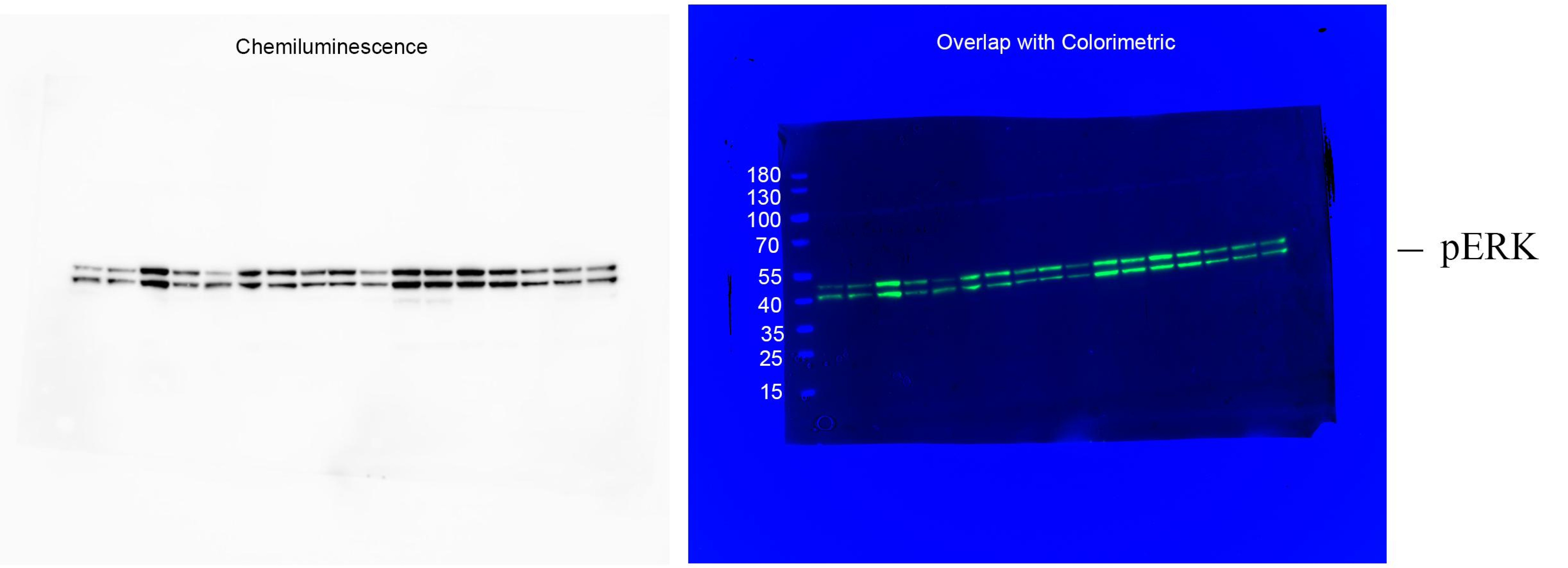

ERK

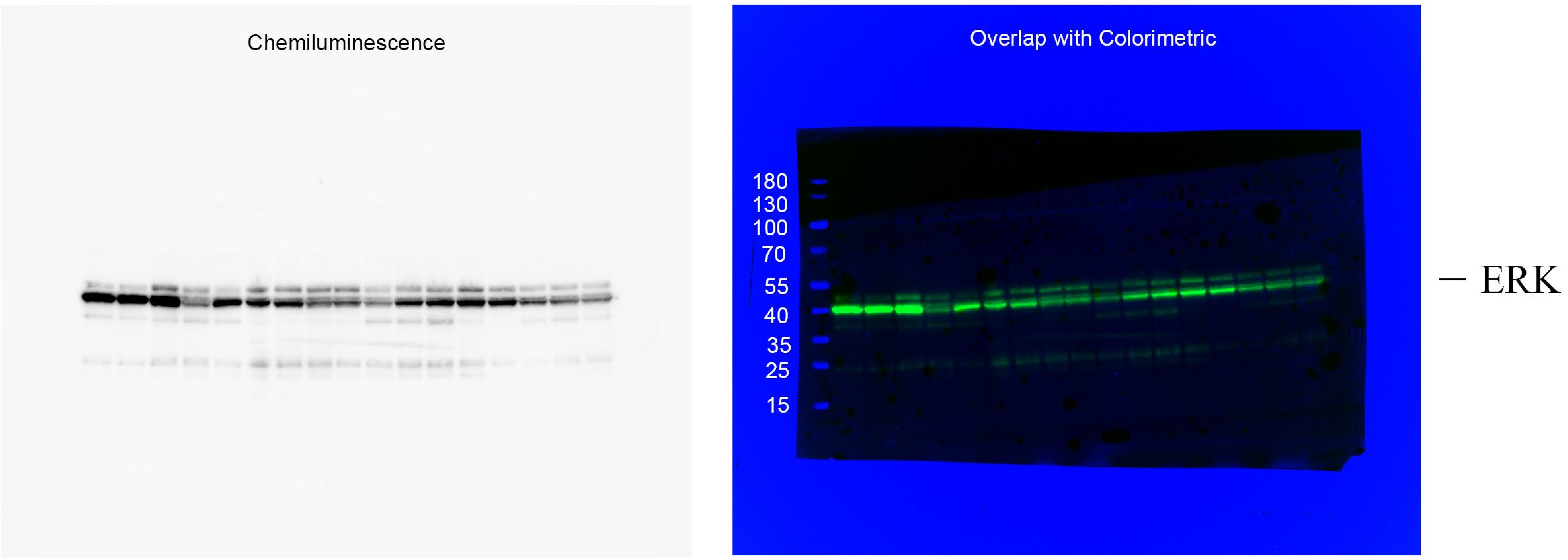

GAPDH

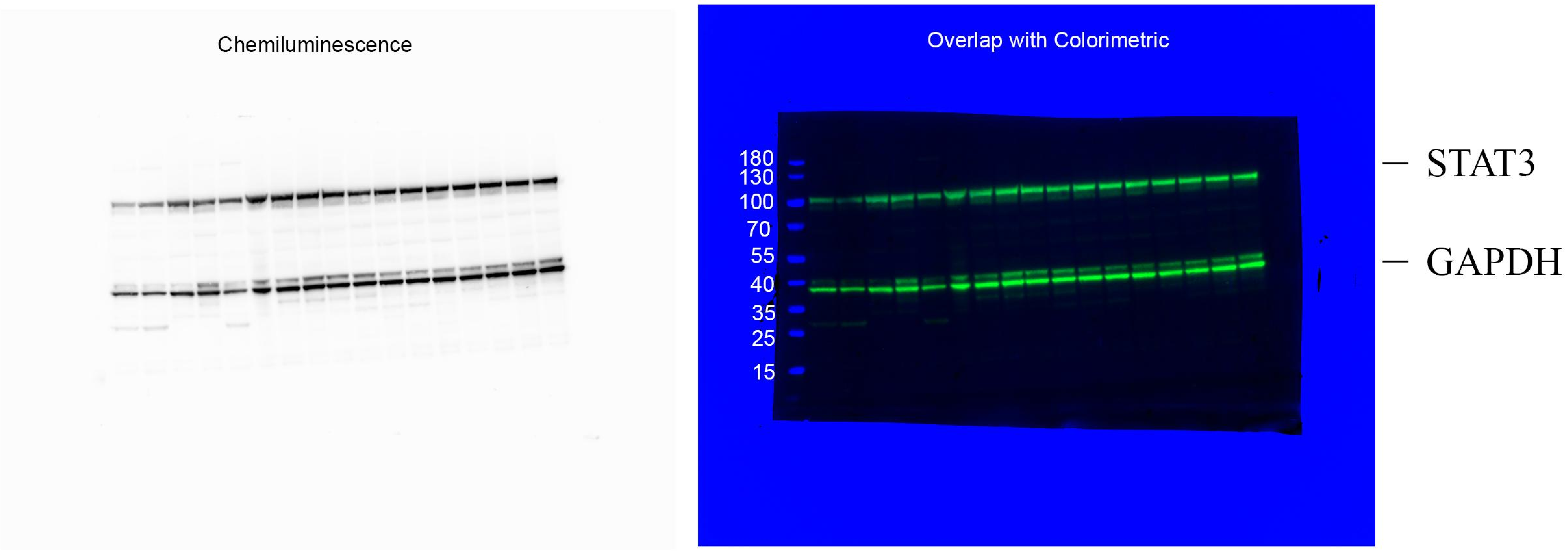

Figure 5J

pSTAT3

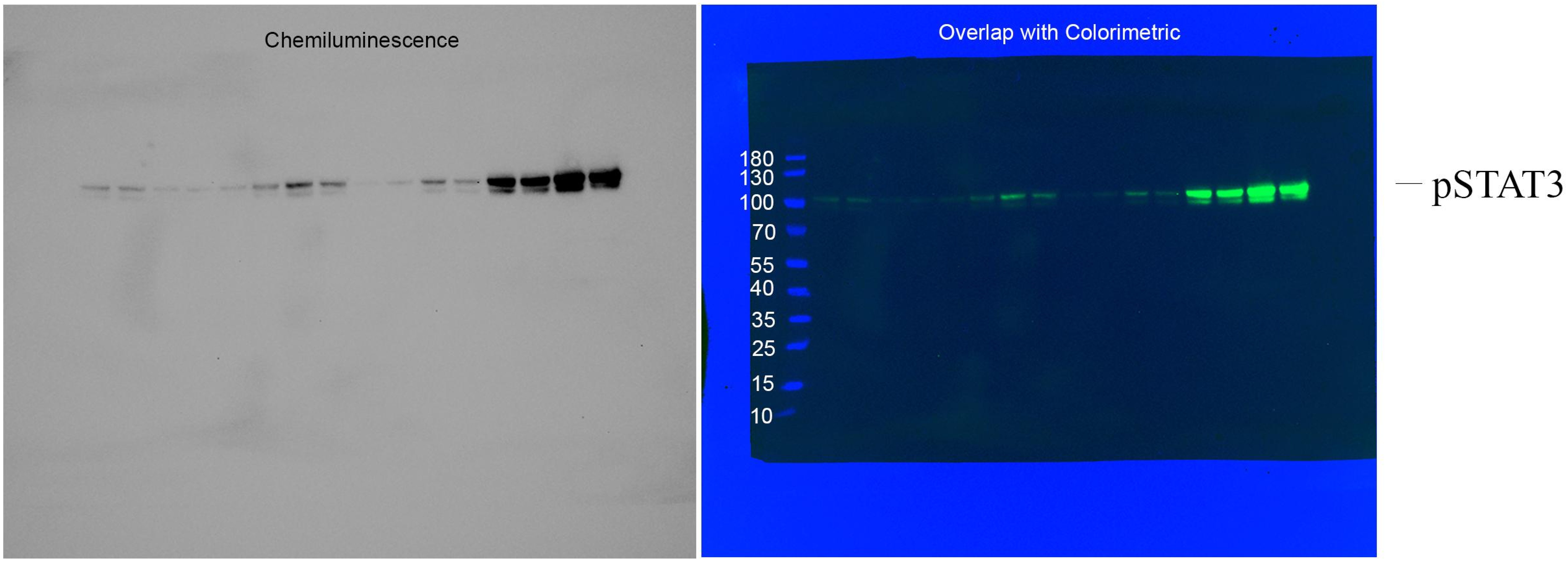

STAT3

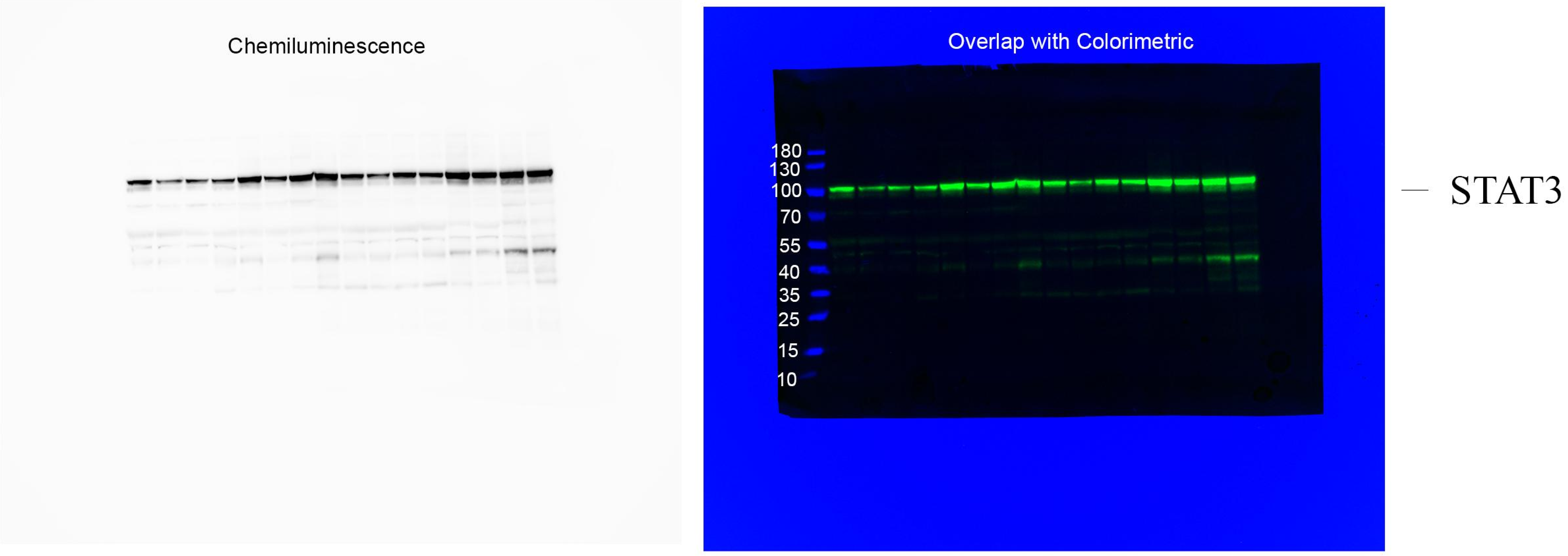

GAPDH

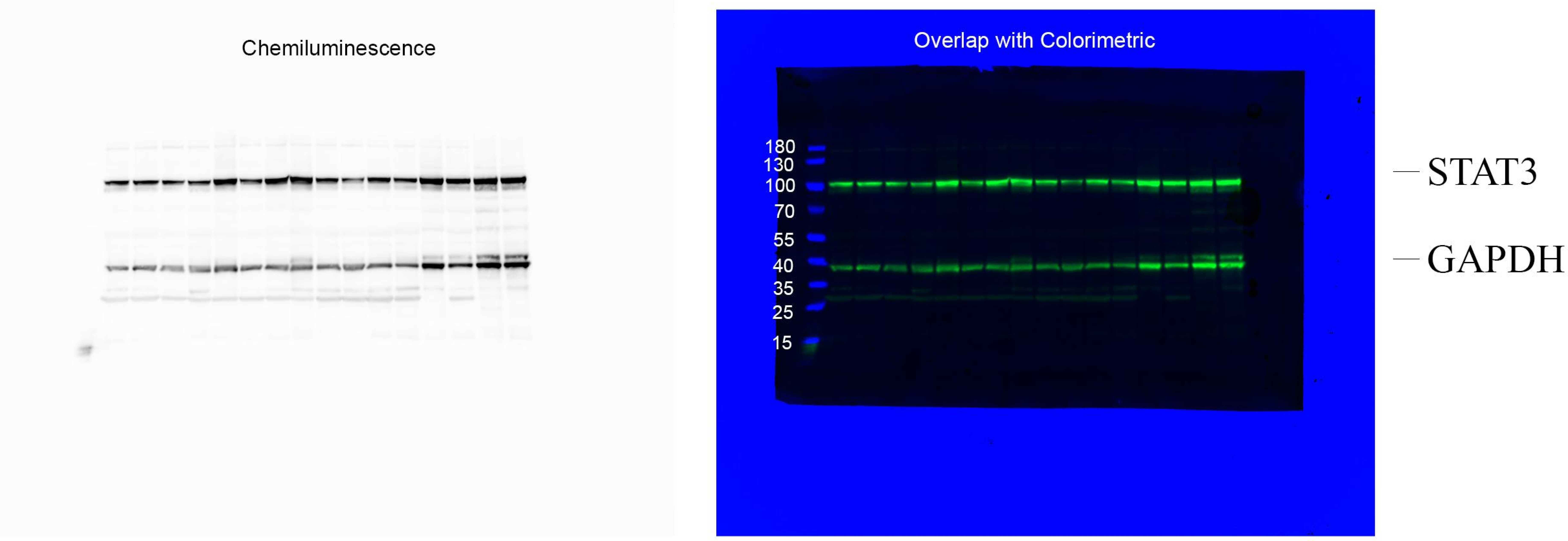

Figure 5K -- WT vs NiC Quantificaton

pSTAT3

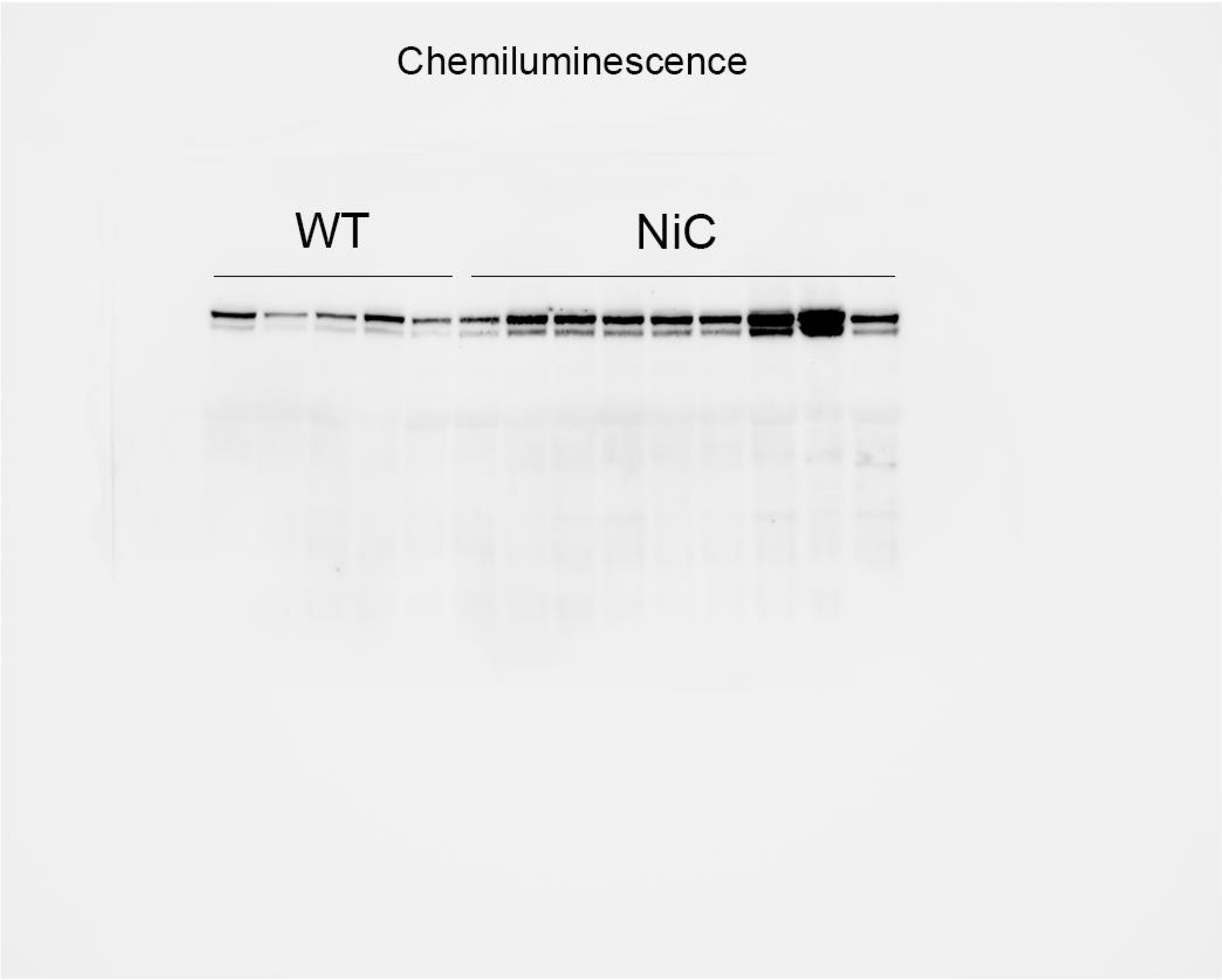

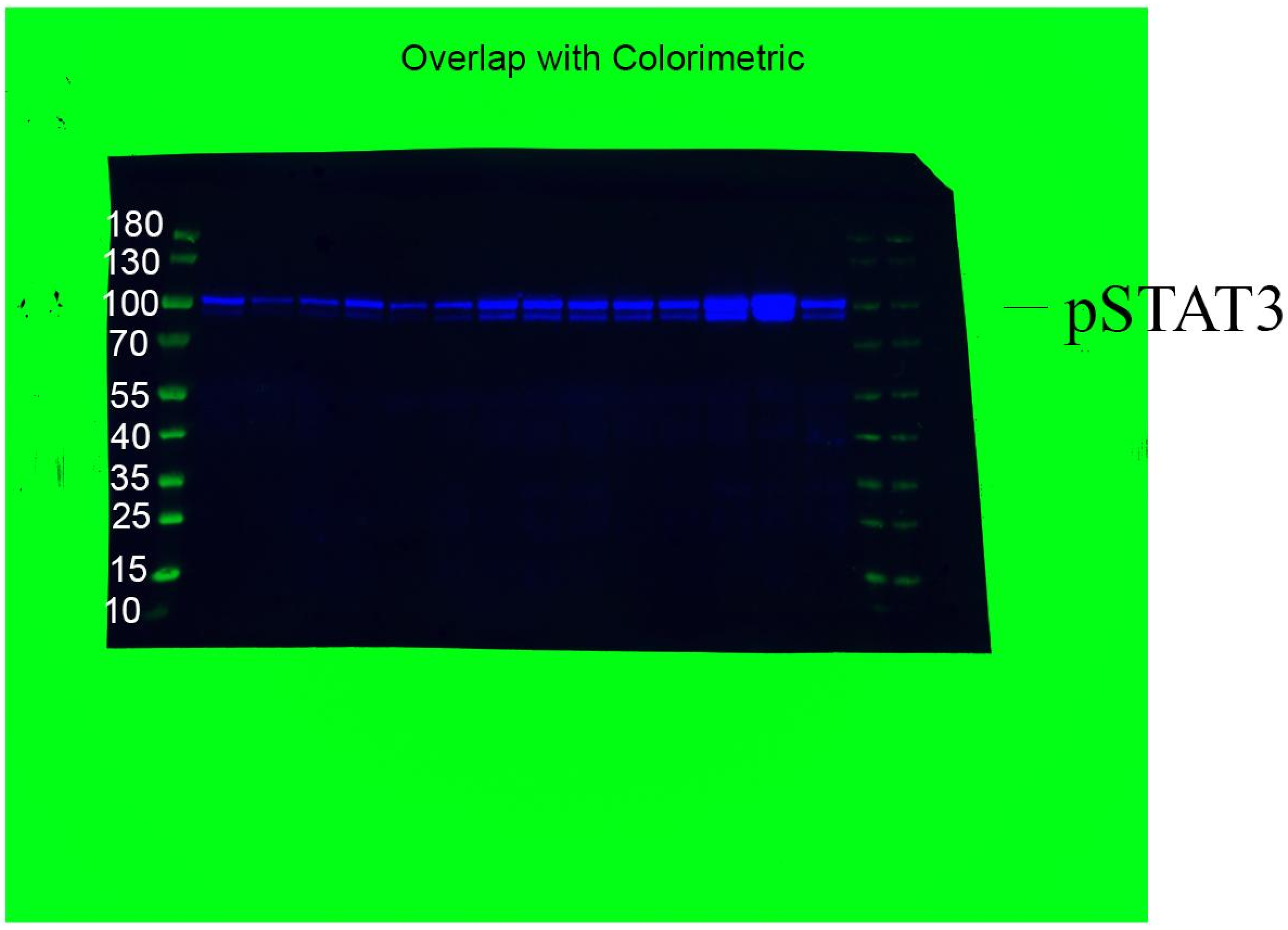

STAT3

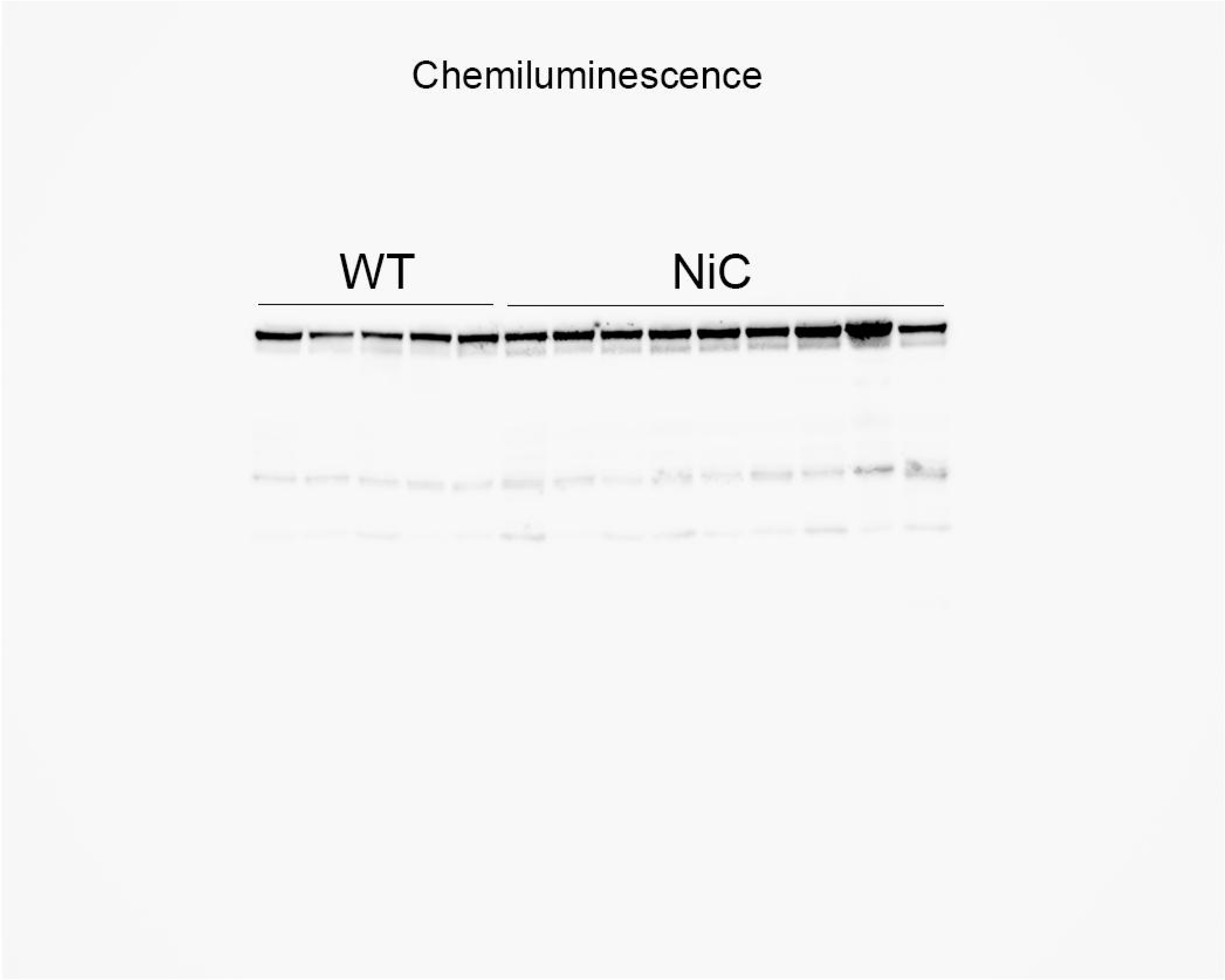

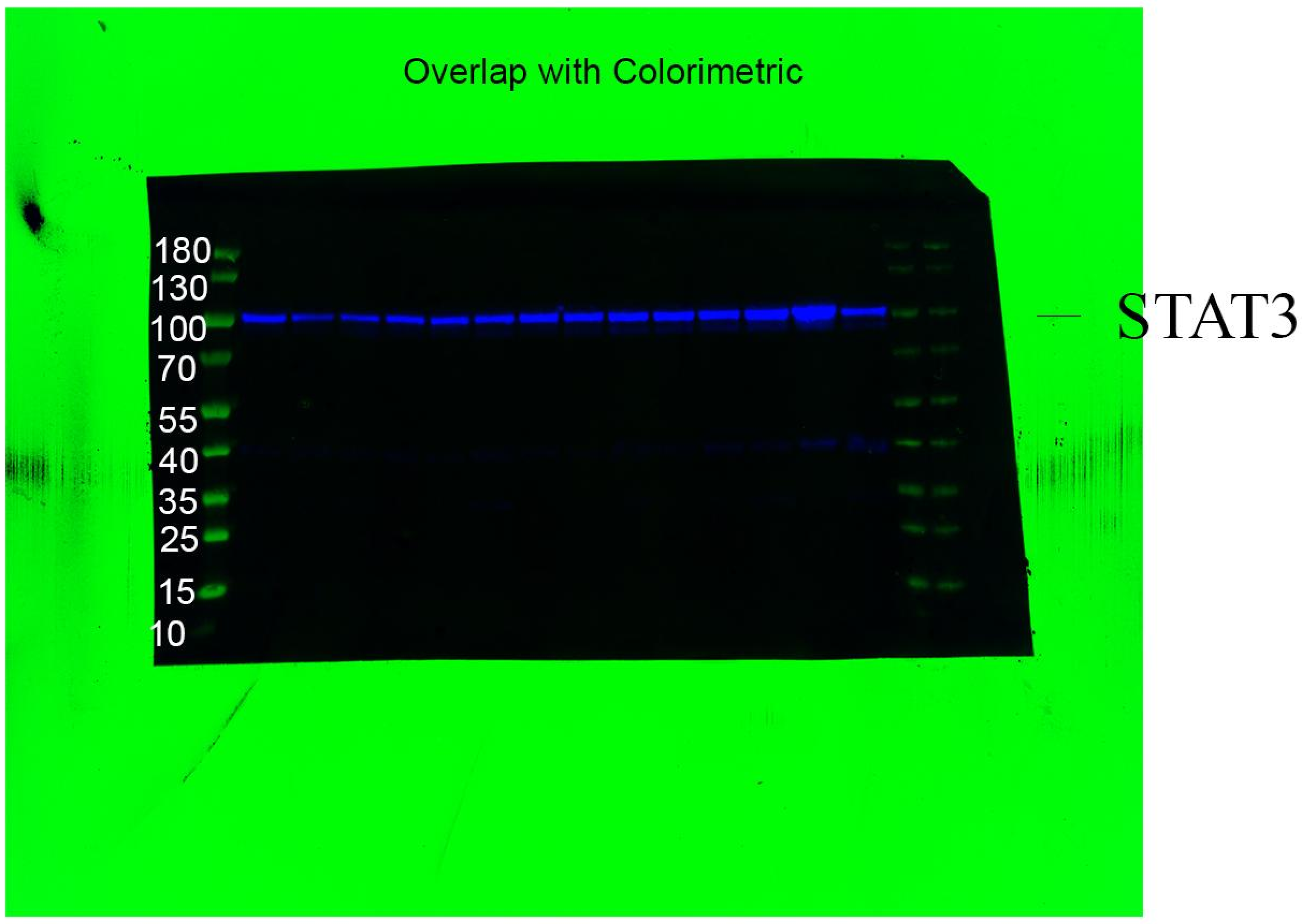

GAPDH

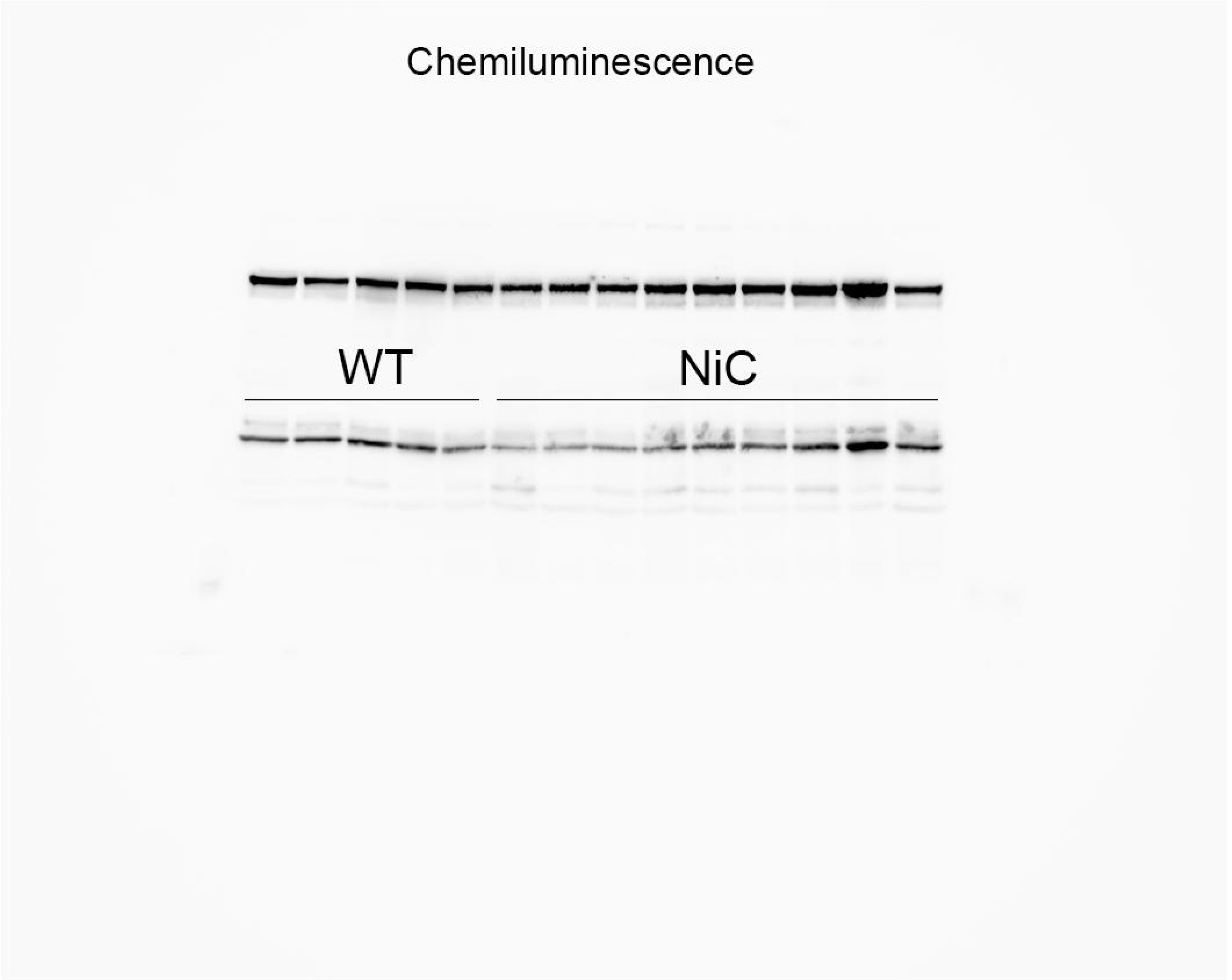

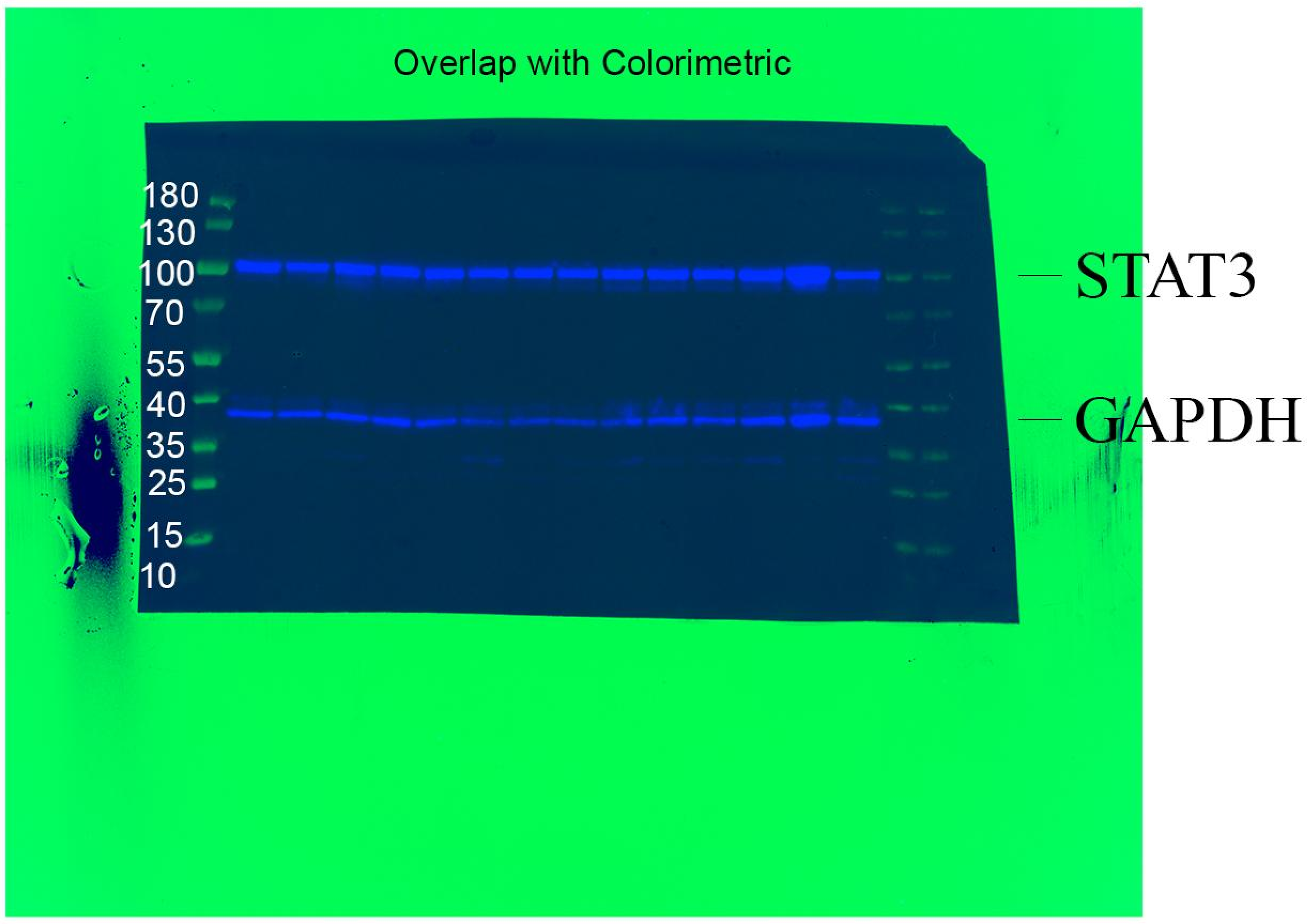

Figure 5K -- KC vs KNiC Quantificaton

pSTAT3

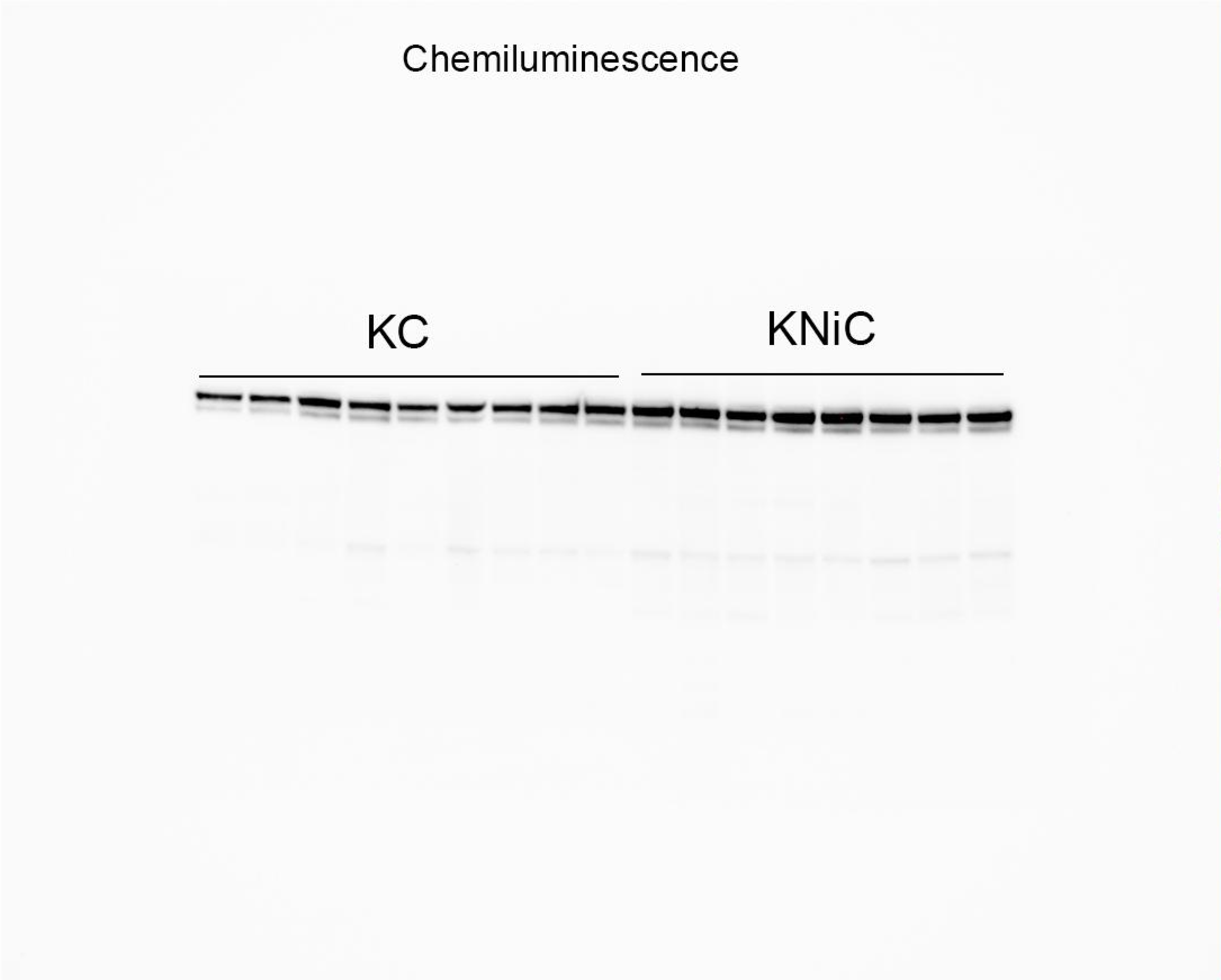

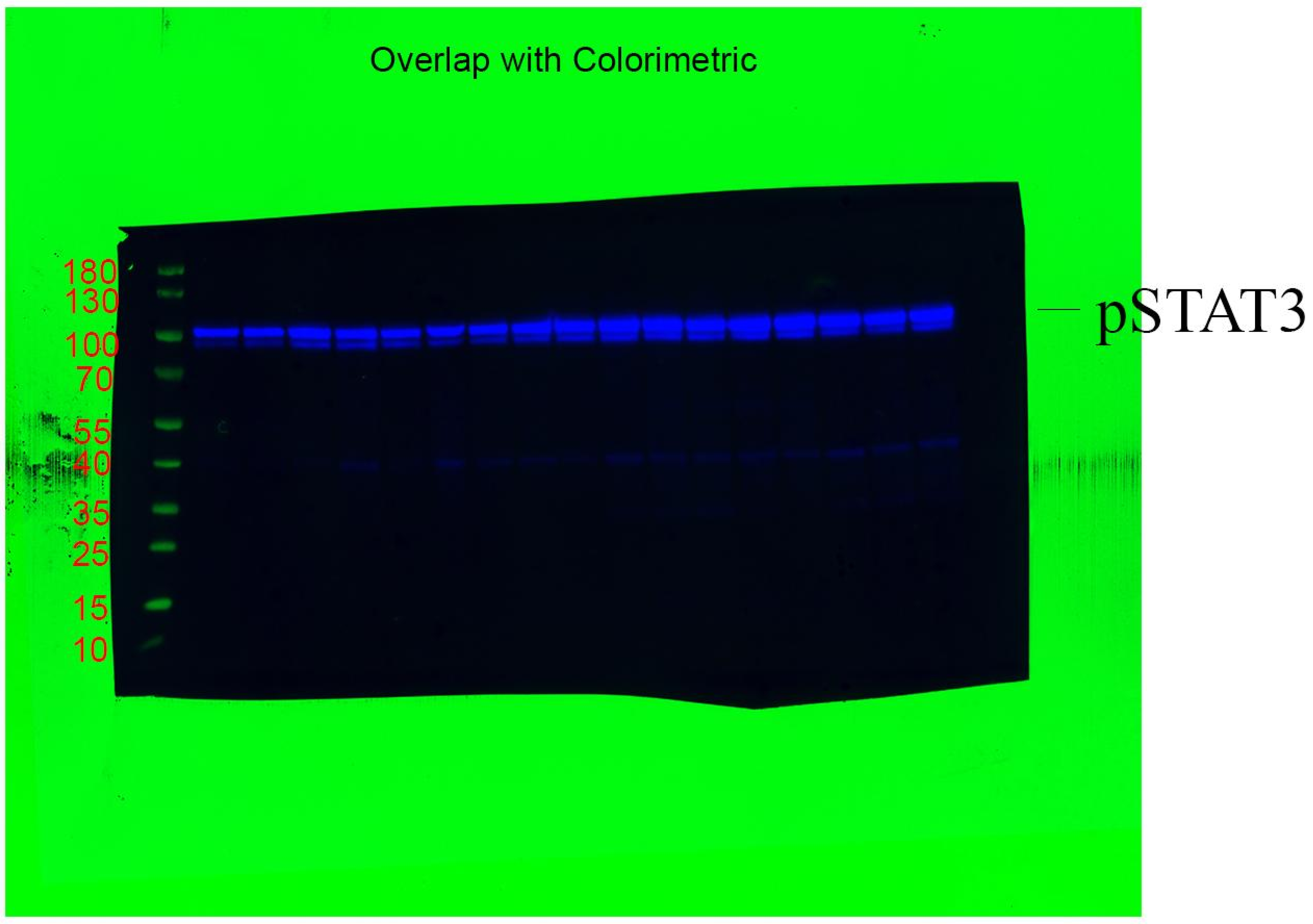

STAT3

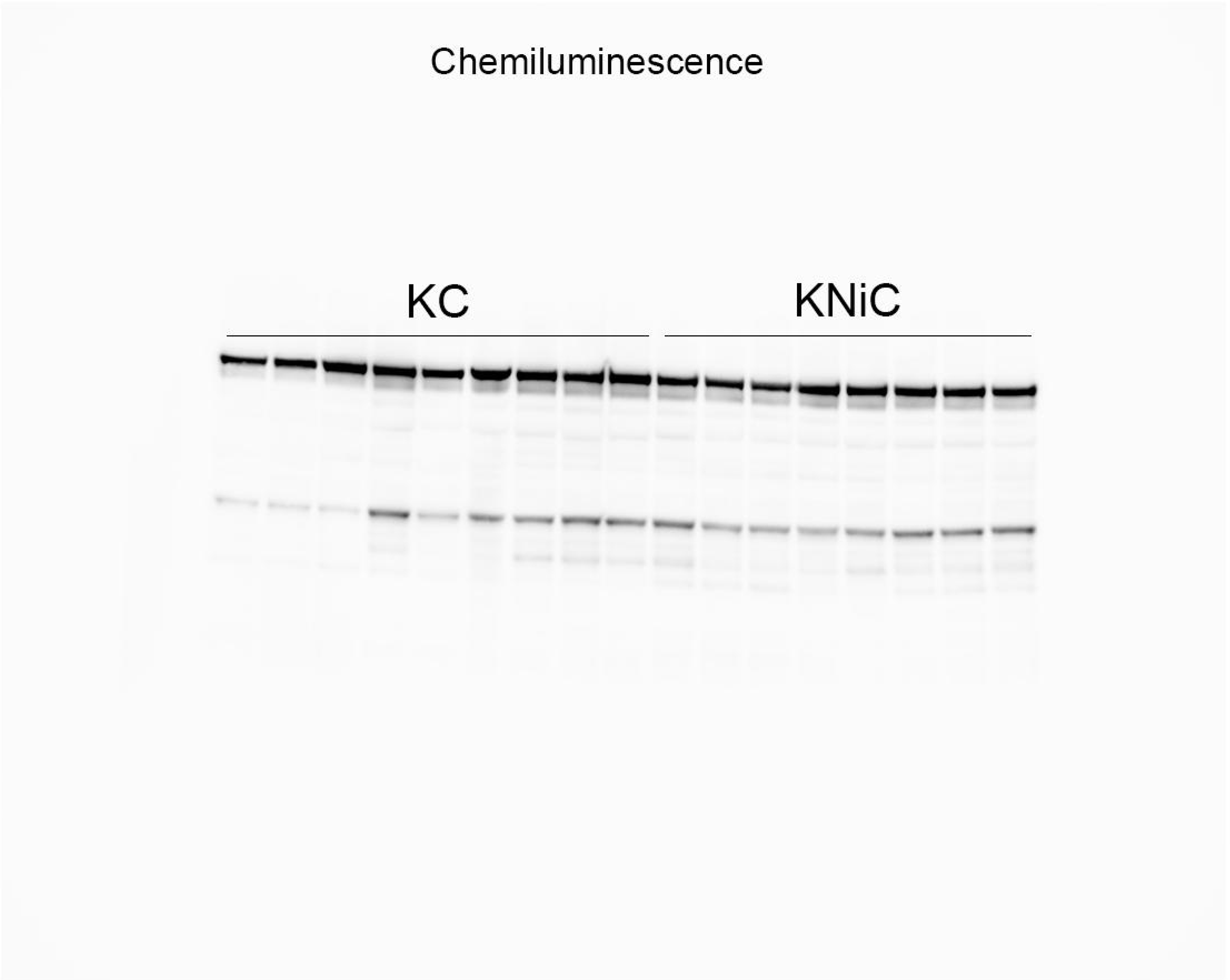

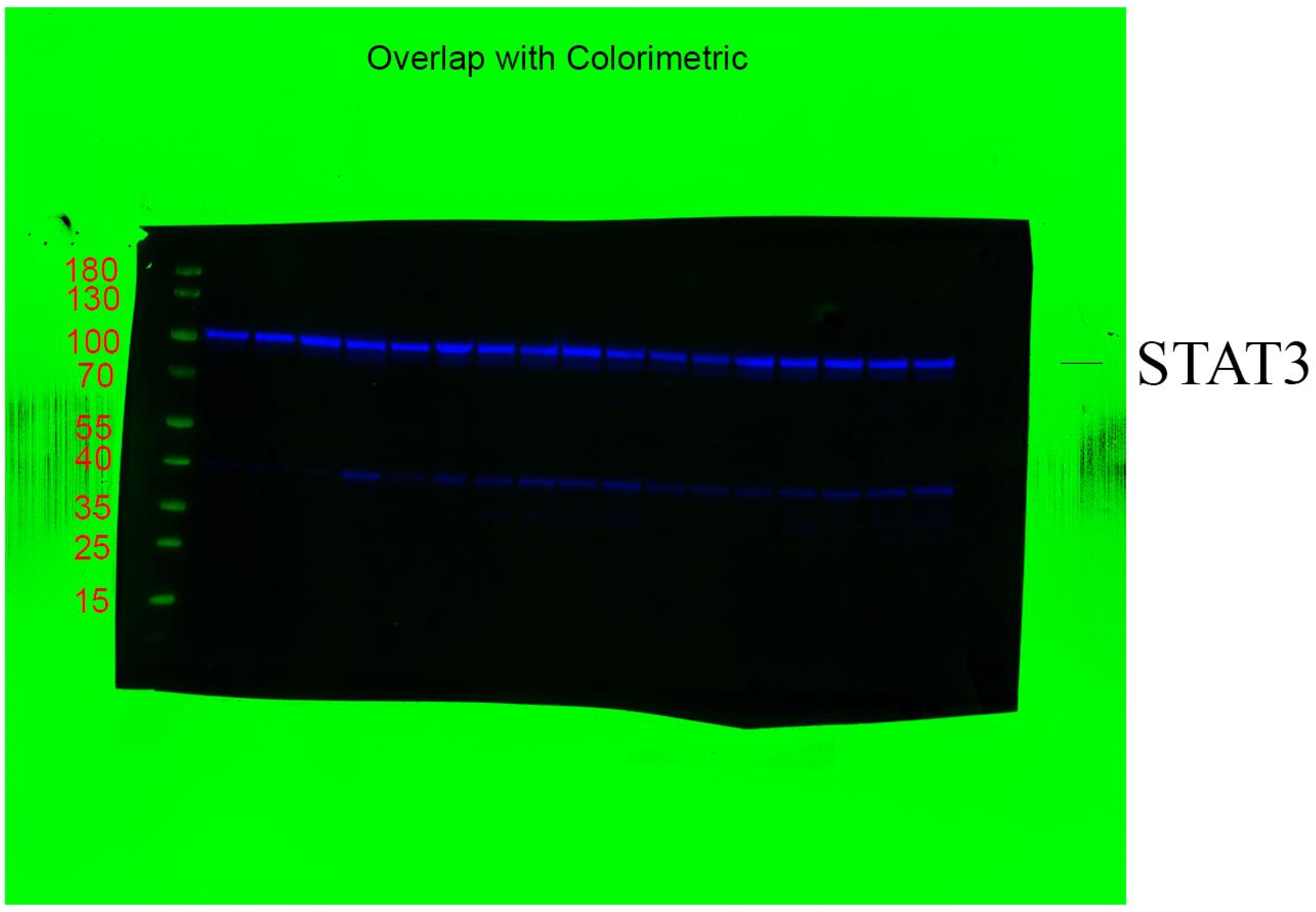

GAPDH

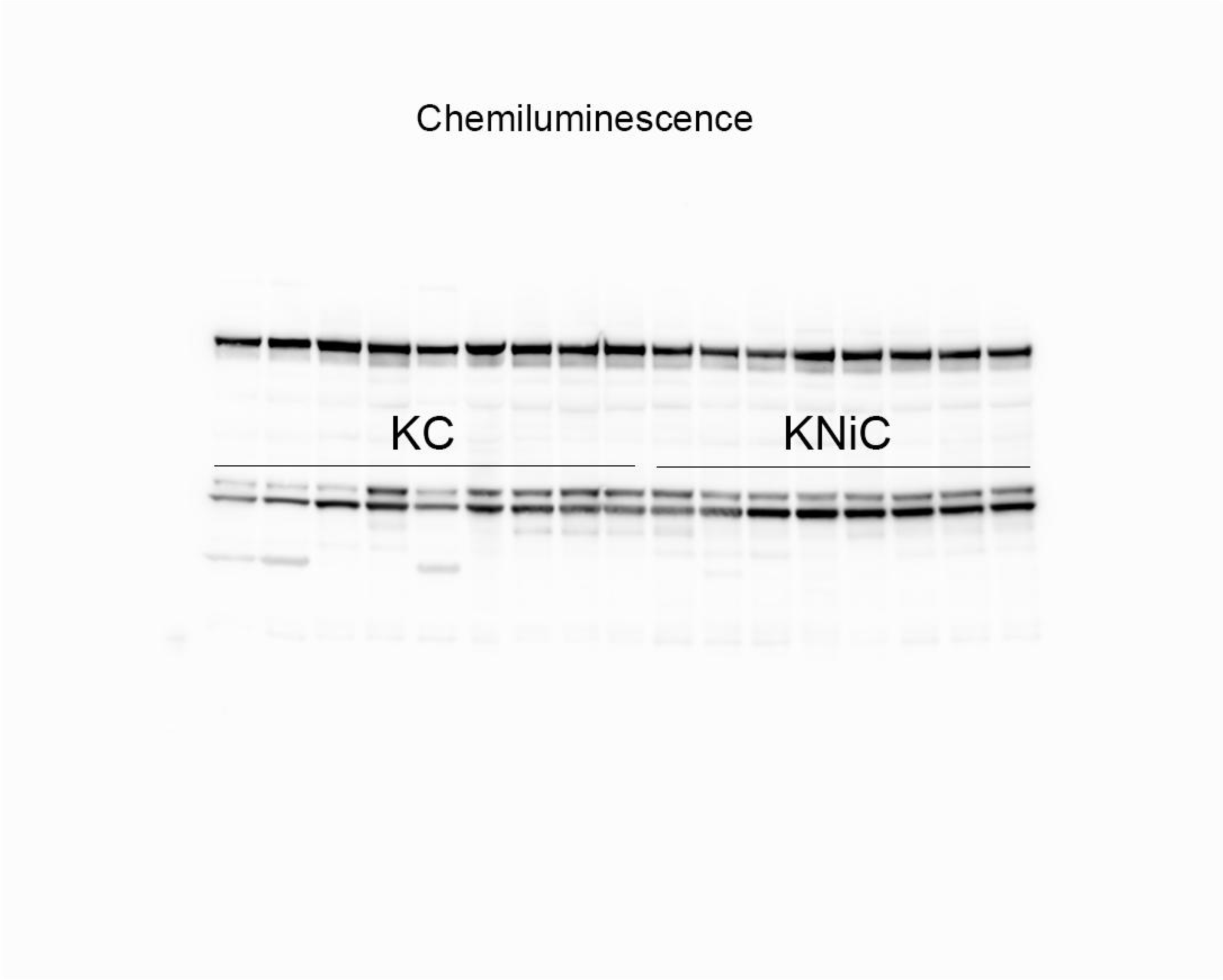

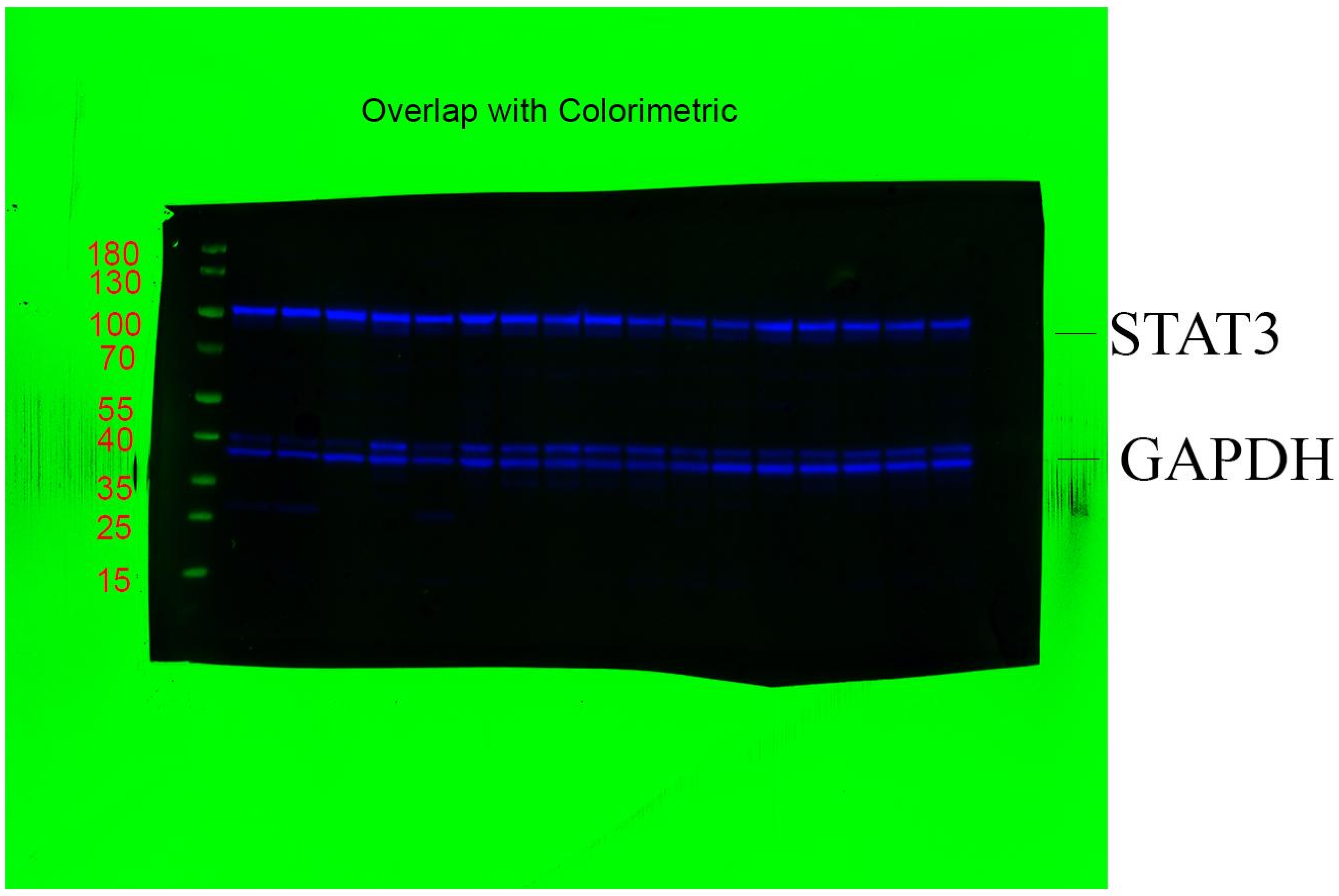

Figure S1B&S3B

RelB

Chemiluminescence

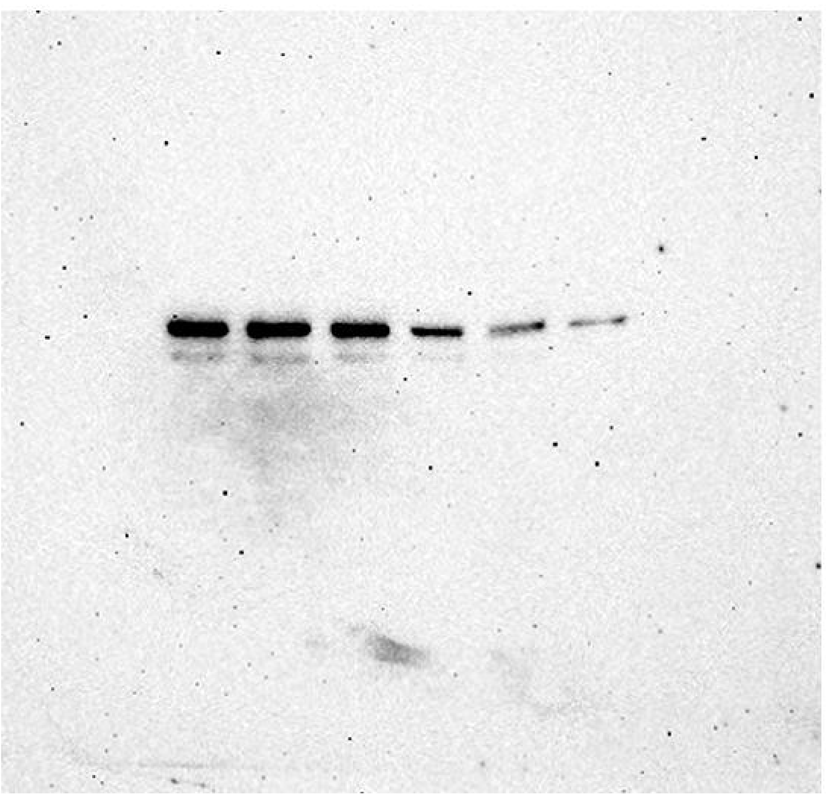

Overlap with Colorimetric

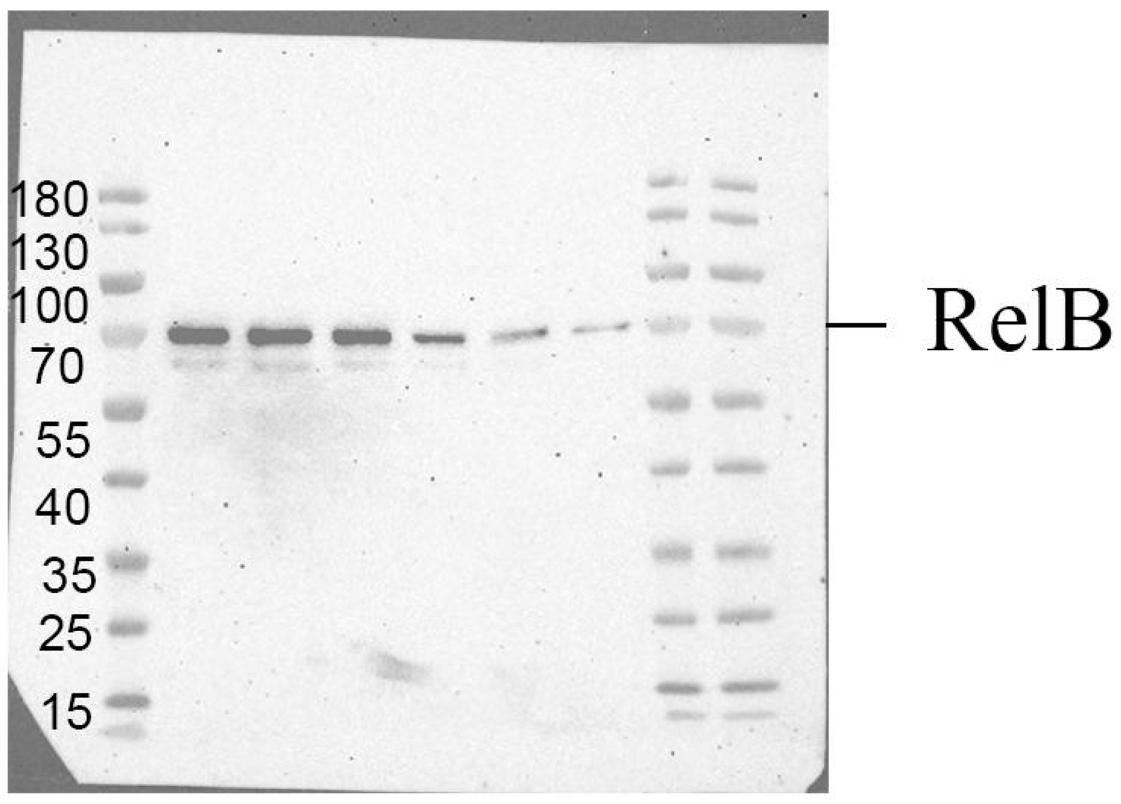

p65

Chemiluminescence

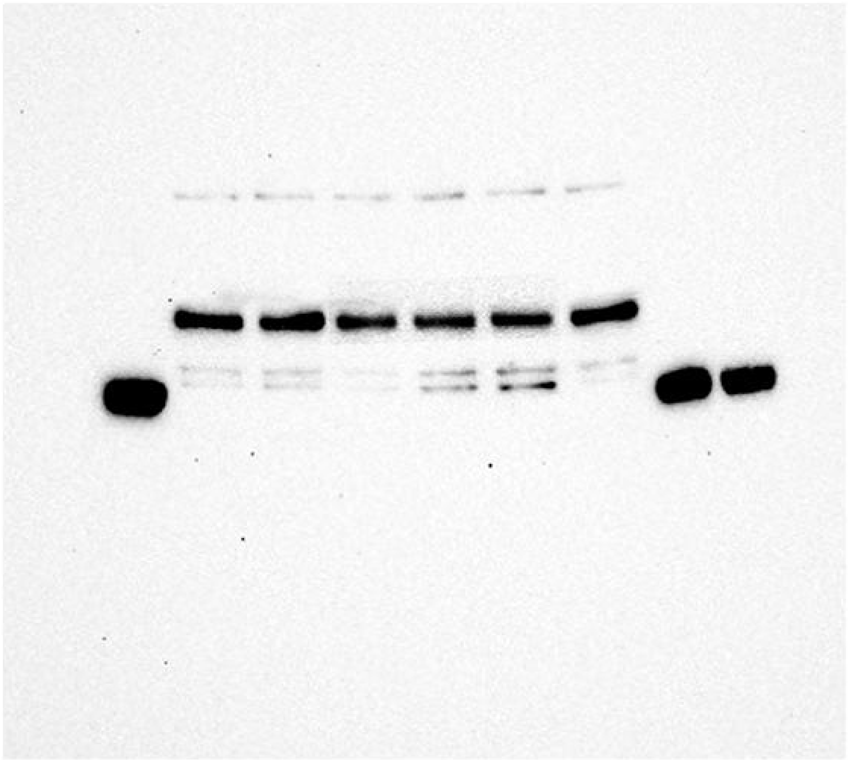

Overlap with Colorimetric

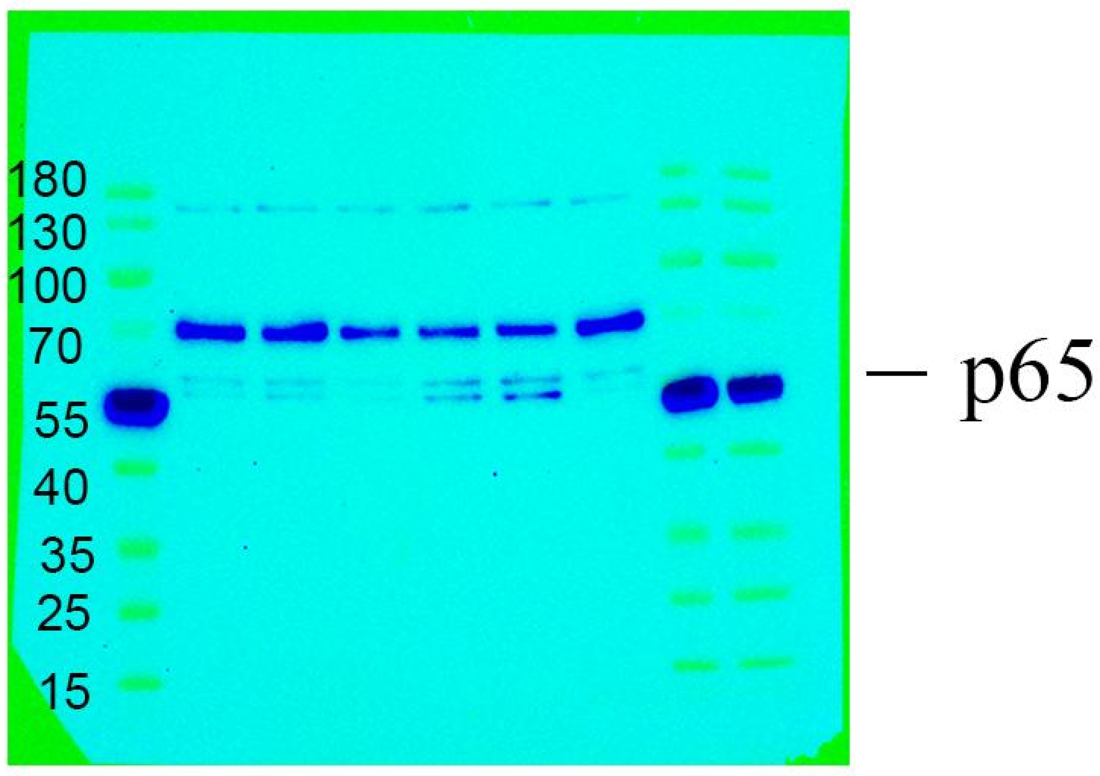

Histone H3

Chemiluminescence

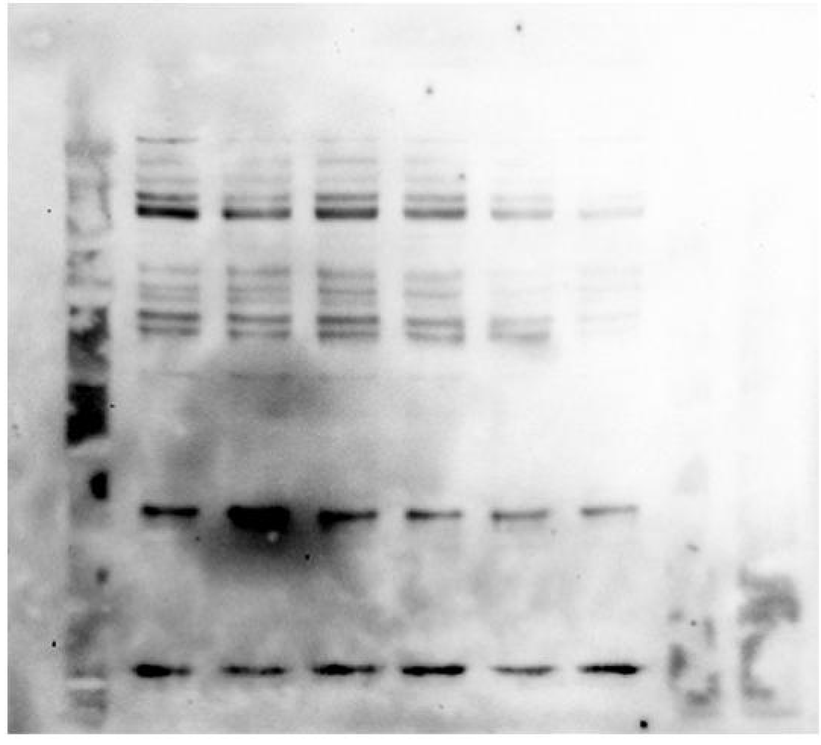

Overlap with Colorimetric

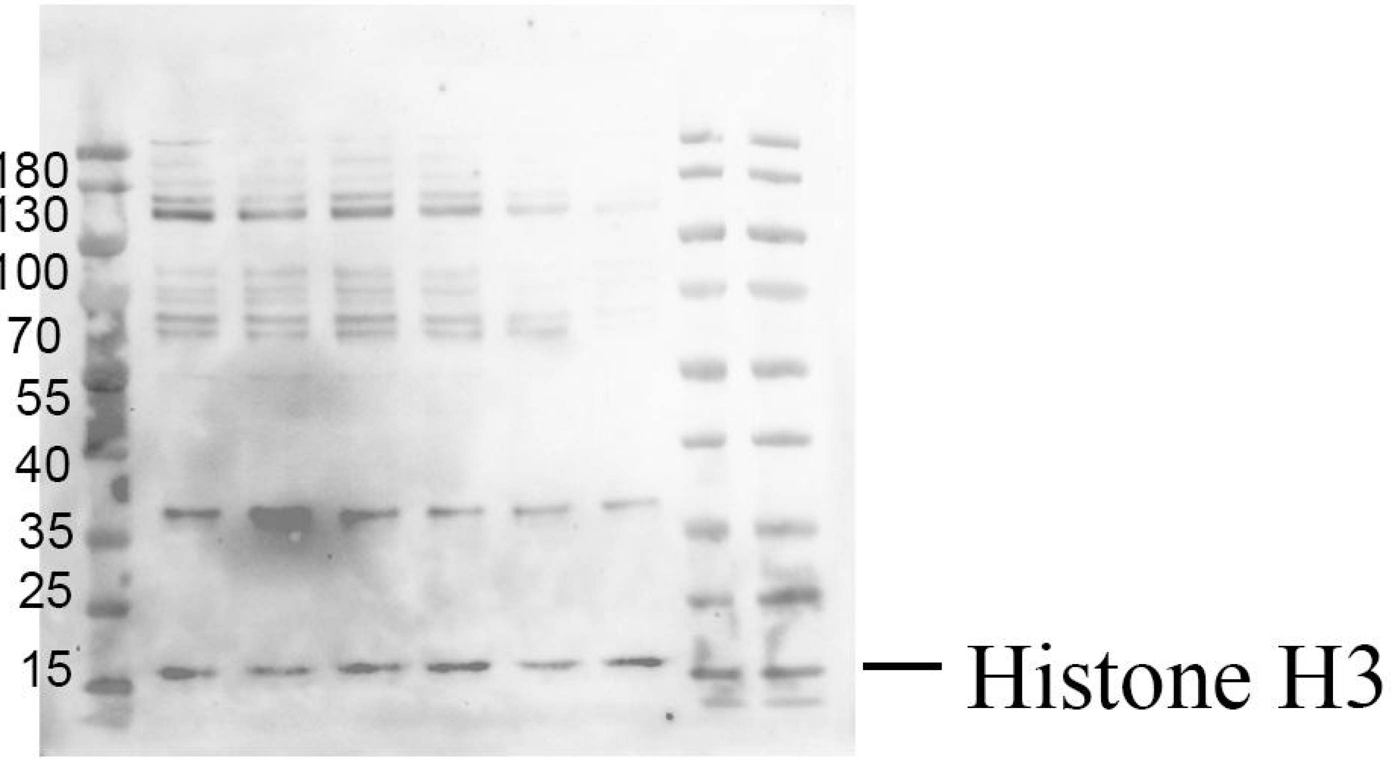

Supplement: Supplementary file 2 — uncropped gels and blots [file 41419_2026_8877_MOESM2_ESM.pdf]
